# Supplementary material for: Chemical evolution of an autonomous DNAzyme with allele-specific gene silencing activity
Source: Nat Commun. 2023 Apr 27;14:2413. doi: 10.1038/s41467-023-38100-9 (PMC10140269; doi:10.1038/s41467-023-38100-9)
Supplement: Supplementary file 1 — Supplementary Information [file 41467_2023_38100_MOESM1_ESM.pdf]

Supplementary Information

Chemical Evolution of an Autonomous DNAzyme with Allele-Specific Gene Silencing Activity

Kim Nguyen[1†], Turnee N. Malik[1†], and John C. Chaput[1-4*]

[1]Department of Pharmaceutical Sciences, University of California, Irvine, CA 92697-3958 USA
[2]Department of Chemistry, University of California, Irvine, CA 92697-3958 USA
[3]Department of Molecular Biology and Biochemistry, University of California, CA 92697-3958 USA
[4]Department of Chemical and Biomolecular Engineering, University of California, Irvine, CA 92697-3958 USA

Table of contents

Supplementary Figures

Figure S1. Structural views of DNAzyme 10-23.
Figure S2. Mechanism of DNAzyme-mediated RNA cleavage
Figure S3. Structure activity relationship analysis of position 14 in the catalytic loop of DNAzyme 10-23
Figure S4. Structure activity relationship analysis of 10-23 variants prepared with modified binding arms, G14 modification and a phosphodiester linkage at position 0-1 of the catalytic loop
Figure S5. 2'-Methoxy walk of the catalytic loop for double modifications that enhance activity
Figure S6. Structure-activity analysis of positions 7, 8, and 14 in the catalytic loop with 2'-methoxy residues
Figure S7. Structure-activity analysis of positions 7, 8, and 14 in the catalytic loop with 2'-methoxyethoxy residues
Figure S8. Structure-activity analysis of positions 7, 8, and 14 with a phosphorothioate linkage at position 0-1 in the catalytic loop.
Figure S9. Chemical optimization of 10-23 variant 13
Figure S10. Chemical optimization of 10-23 variant 42 measured at 100:1 (S:E)
Figure S11. Summary of 10-23 Designs Evaluated in Kinetic Cleavage Assays
Figure S12. Multiple turnover kinetic analysis of 10-23 variants measured at 10:1 substrate to enzyme concentration
Figure S13. Transferability of Dz 46 chemistry to other targets
Figure S14. Multiple turnover kinetic analysis comparing variant 46 to known methoxy derivatives at 100:1 substrate to enzyme concentration
Figure S15. Magnesium dependency of engineered 10-23 variants measured at 100:1 substrate to enzyme concentration
Figure S16. Activity of Dz 46 under reduced concentrations of magnesium
Figure S17. Nearest neighbor analysis of unmodified Dz 1
Figure S18. Analysis of Dz 46 cleavage motif preferences in long RNA substrates
Figure S19. Analysis of Dz 1 cleavage preferences in long RNA substrates
Figure S20. Evaluation of allele-specific cleavage by Dz 46 under multiple turnover conditions in the presence of RNase H
Figure S21. Absolute values observed for allele-specific knock-down

Supplementary Tables

Table S1. G12V KRAS targeting DNAzymes used in chemical evolution screening
Table S2. Control oligos (active, inactive, and non-binding DNAzymes, ASO)
Table S3. DNAzymes used to define the preferred cleavage motif
Table S4. RNA substrates
Table S5. *Hs*RNAse H1 level in human cell lines measured by ELISA

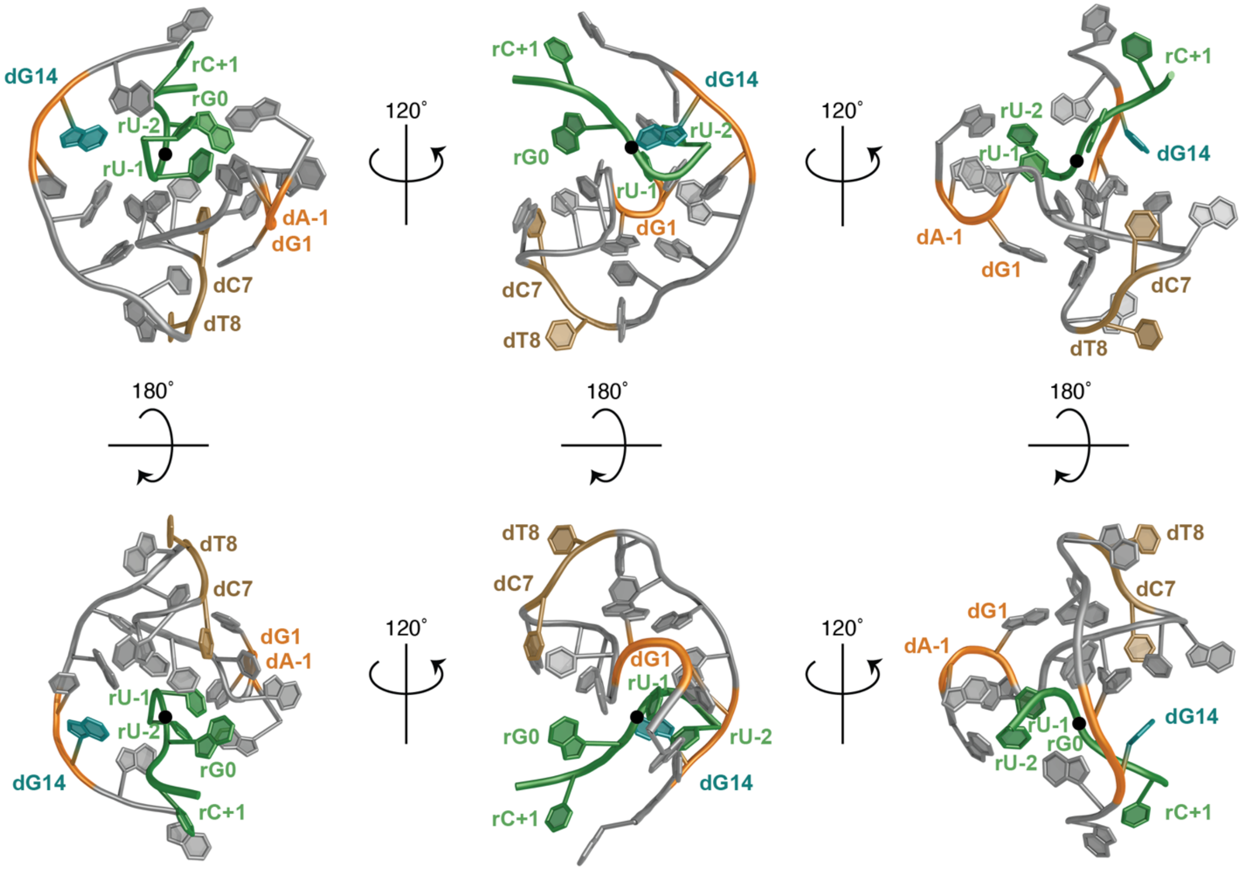

Supplementary Figure 1. Structural views of DNAzyme 10-23. Cartoon representation of an NMR-averaged precatalytic structure of 10-23 bound to an RNA substrate (green) encoding a prion protein (PDB: 7PDU). Structural views observed at 120° and 240° horizonal rotations and vertical rotations of 180°.

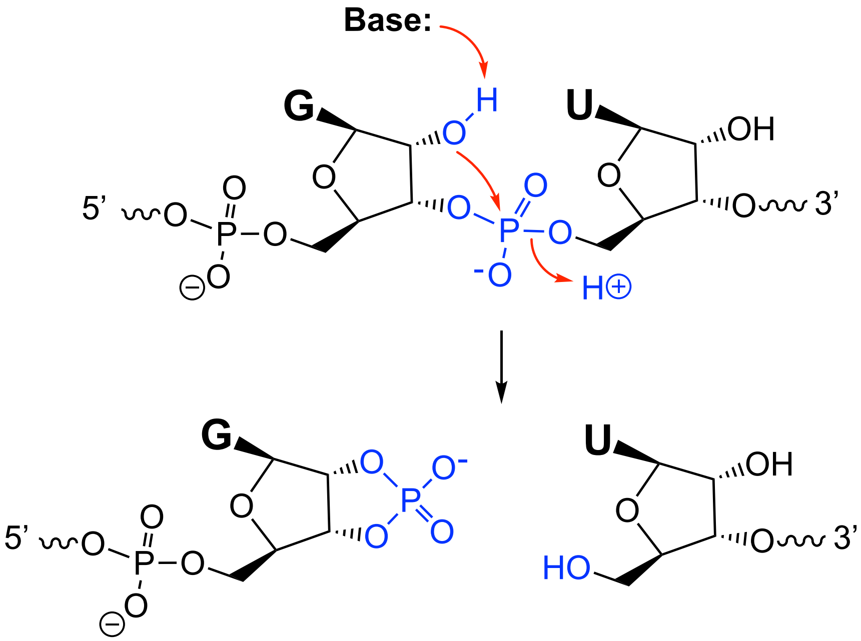

Supplementary Figure 2. Mechanism of DNAzyme-mediated RNA cleavage. Site-specific RNA cleavage occurs by deprotonation of the 2' hydroxyl group of the scissile residue with simultaneous protonation of the 5' hydroxyl group of the cleaved bond. In-line attack of the resulting 2' oxyanion on the adjacent phosphate results in the formation of an upstream cleavage product carrying cyclic phosphate and downstream cleavage product with a hydroxyl group.

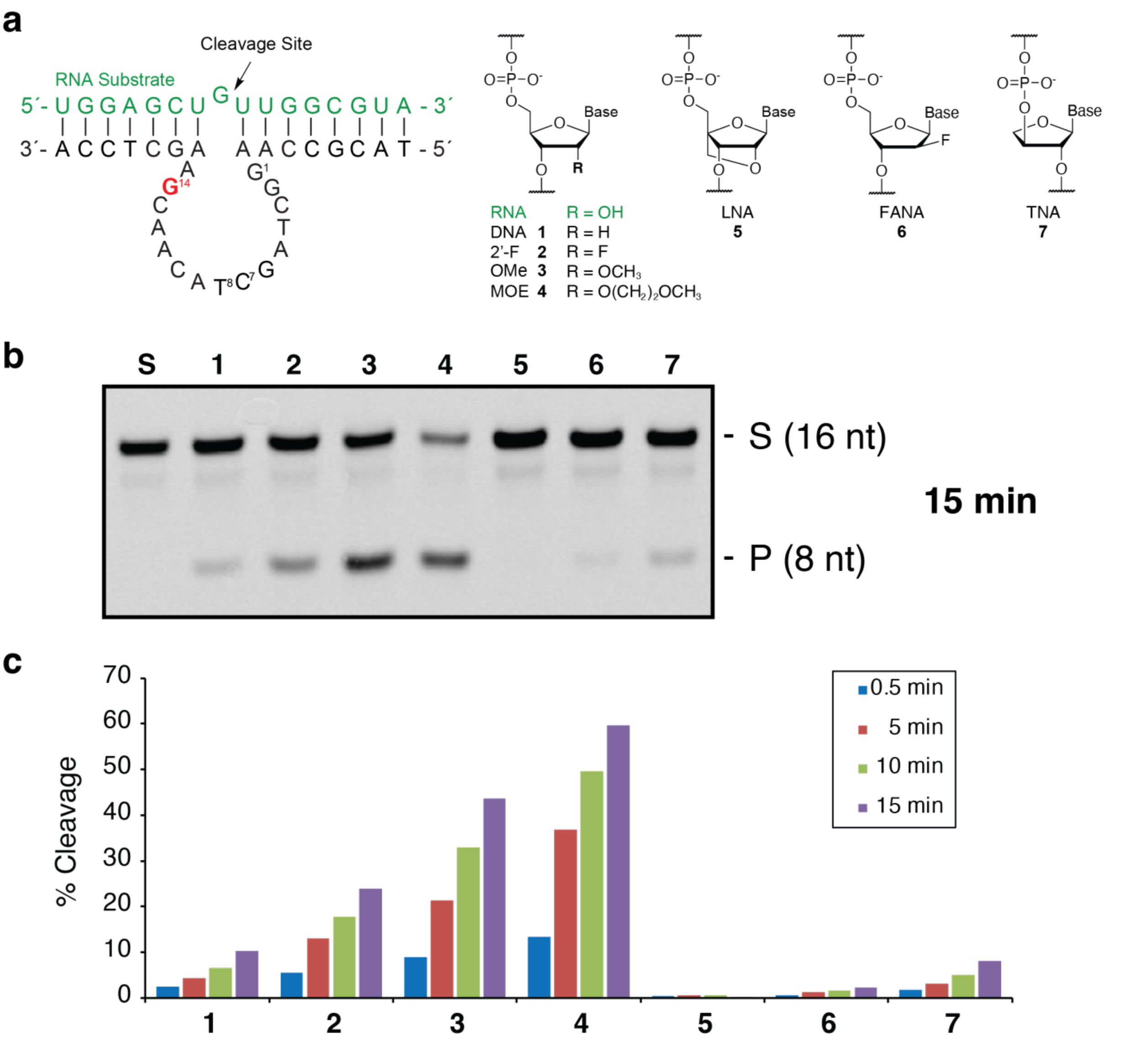

Supplementary Figure 3. Structure activity relationship analysis of position 14 in the catalytic loop of DNAzyme 10-23. a. Nucleic acid sequence of DNAzyme 10-23 targeting a segment of KRAS G12V mRNA and chemical structures of sugar modified analogs. Abbreviations of nucleotide analogs: 2'-F (2'-deoxy-2'-fluororibonucleic acid), OMe (2'-deoxy-2'-methoxyribonucleic acid), MOE (2'-deoxy-2'-methoxyethoxyribonucleic acid), LNA (locked nucleic acid), FANA (2'-fluoroarabinonucleic acid), and TNA (threose nucleic acid). **b-c.** Representative gel and bar graph showing the cleavage activity of each analog (n = 1). All reactions were performed in simulated physiological buffer containing 1 mM $MgCl_2$, 50 mM Tris (pH 7.5), 10 mM NaCl, and 140 mM KCl at 37°C with 500 nM substrate and 50 nM enzyme (10:1, S:E). S: 5'-Cy5 labeled full-length substrate, P: 5'-Cy5 labeled cleavage product. Molecular weight markers indicated to the right of the gel.

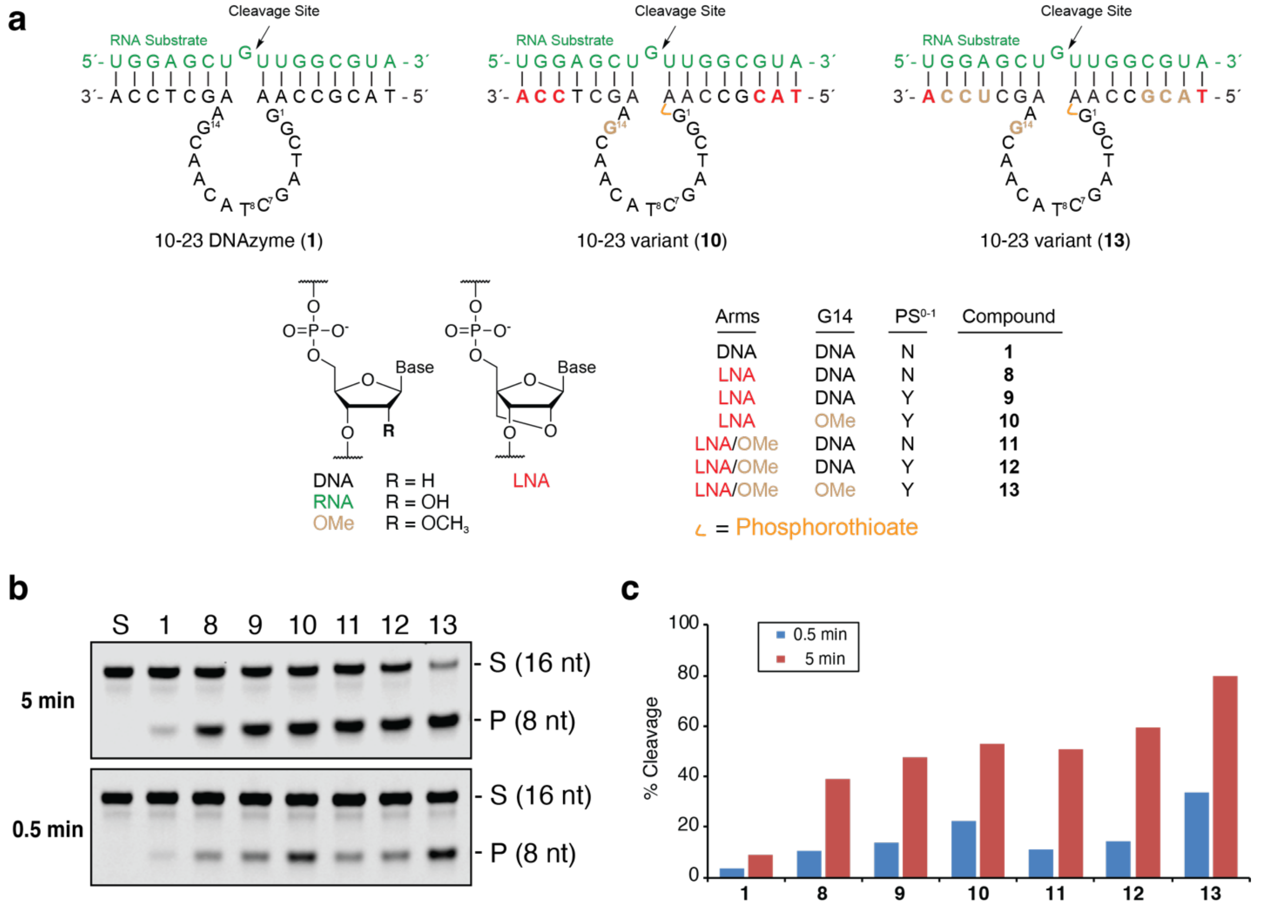

Supplementary Figure 4. Structure activity relationship analysis of 10-23 variants prepared with modified binding arms, G14 modification and a phosphodiester linkage at position 0-1 of the catalytic loop. a. Sequences of DNAzyme 10-23 (wild-type and engineered variants) targeting a segment of KRAS G12V mRNA and chemical structures of sugar modified analogs. Abbreviations of nucleotide analogs: LNA (locked nucleic acid), OMe (2'-deoxy-2'-methoxyribonucleic acid), and phosphorothioate. **b-c.** Representative gels and bar graph showing the cleavage activity of each analog (n = 1). All reactions were performed in simulated physiological buffer containing 1 mM $MgCl_2$, 50 mM Tris (pH 7.5), 10 mM NaCl, and 140 mM KCl at 37°C with 500 nM substrate and 50 nM enzyme (10:1, S:E). S: 5'-Cy5 labeled full-length substrate, P: 5'-Cy5 labeled cleavage product. Molecular weight markers indicated to the right of the gel.

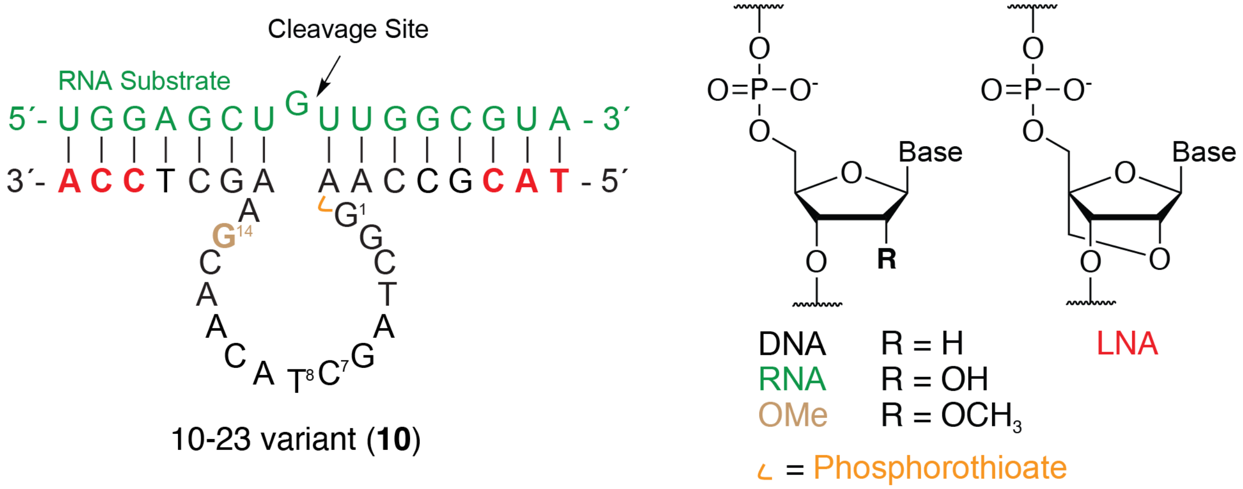

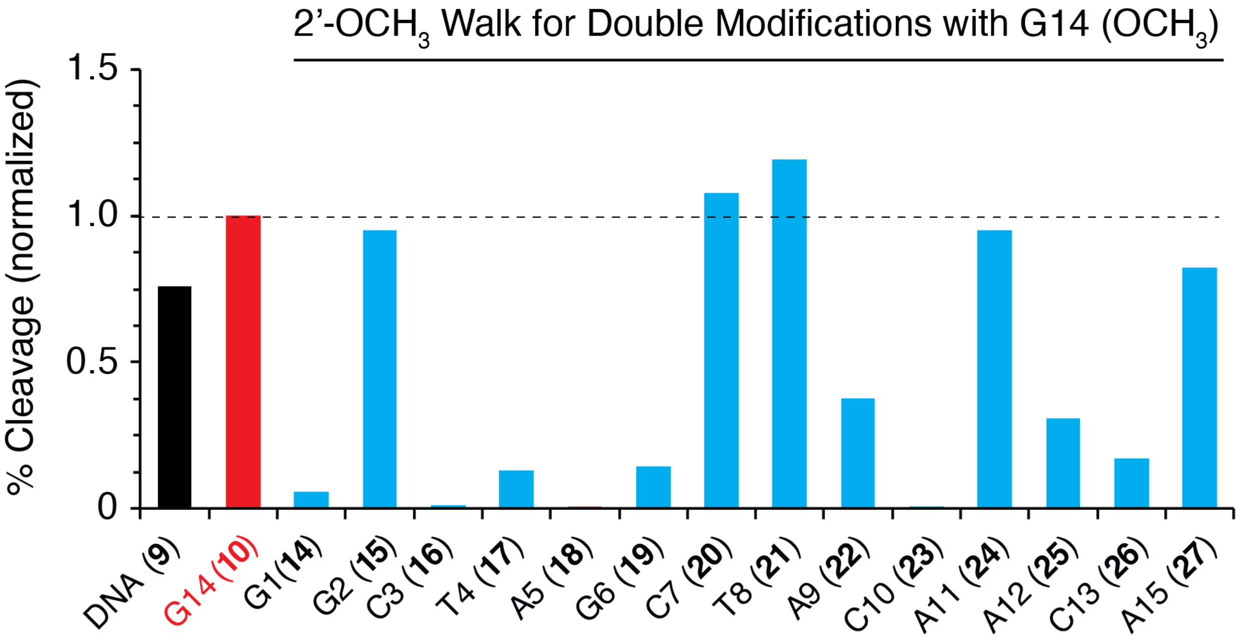

Supplementary Figure 5. 2'-Methoxy walk of the catalytic loop for double modifications that enhance activity. Sequence of DNAzyme 10-23 (variant 10) targeting a segment of KRAS G12V mRNA and chemical structures of sugar modified analogs. Abbreviations of nucleotide analogs: LNA (locked nucleic acid), OMe (2'-deoxy-2'-methoxyribonucleic acid), and phosphorothioate. Bar graph denotes the cleavage activity of each 10-23 variant after a 5 minute incubation at 37°C (n = 1). Colors scheme: 10-23 variant 9 (black), 10-23 variant 10 (red), and 10-23 variant 10 modified with a second OMe residue in the catalytic loop (blue). All reactions were performed in simulated physiological buffer containing 1 mM $MgCl_2$, 50 mM Tris (pH 7.5), 10 mM NaCl, and 140 mM KCl with 500 nM substrate and 50 nM enzyme (10:1, S:E). S: 5'-Cy5 labeled full-length substrate, P: 5'-Cy5 labeled cleavage product.

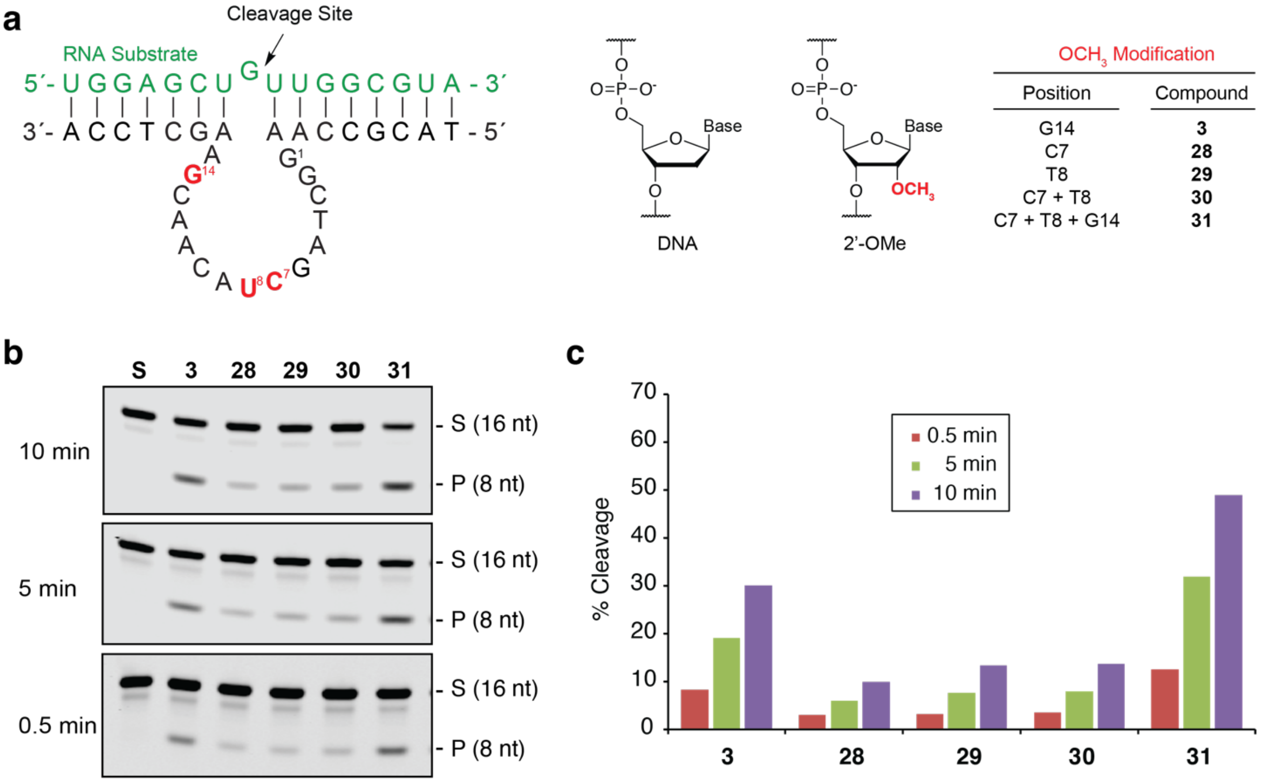

Supplementary Figure 6. Structure-activity analysis of positions 7, 8, and 14 in the catalytic loop with 2'-methoxy residues. a. Sequence of DNAzyme 10-23 targeting a KRAS G12V mRNA segment and chemical structures of sugar modified analogs. Abbreviation: OMe (2'-deoxy-2'-methoxyribonucleic acid). **b-c.** Representative gels and bar graph showing the cleavage activity of each analog (n = 1). All reactions were performed in simulated physiological buffer containing 1 mM $MgCl_2$, 50 mM Tris (pH 7.5), 10 mM NaCl, and 140 mM KCl at 37°C with 500 nM substrate and 50 nM enzyme (10:1, S:E). S: 5'-Cy5 labeled full-length substrate, P: 5'-Cy5 labeled cleavage product. Molecular weight markers indicated to the right of the gel.

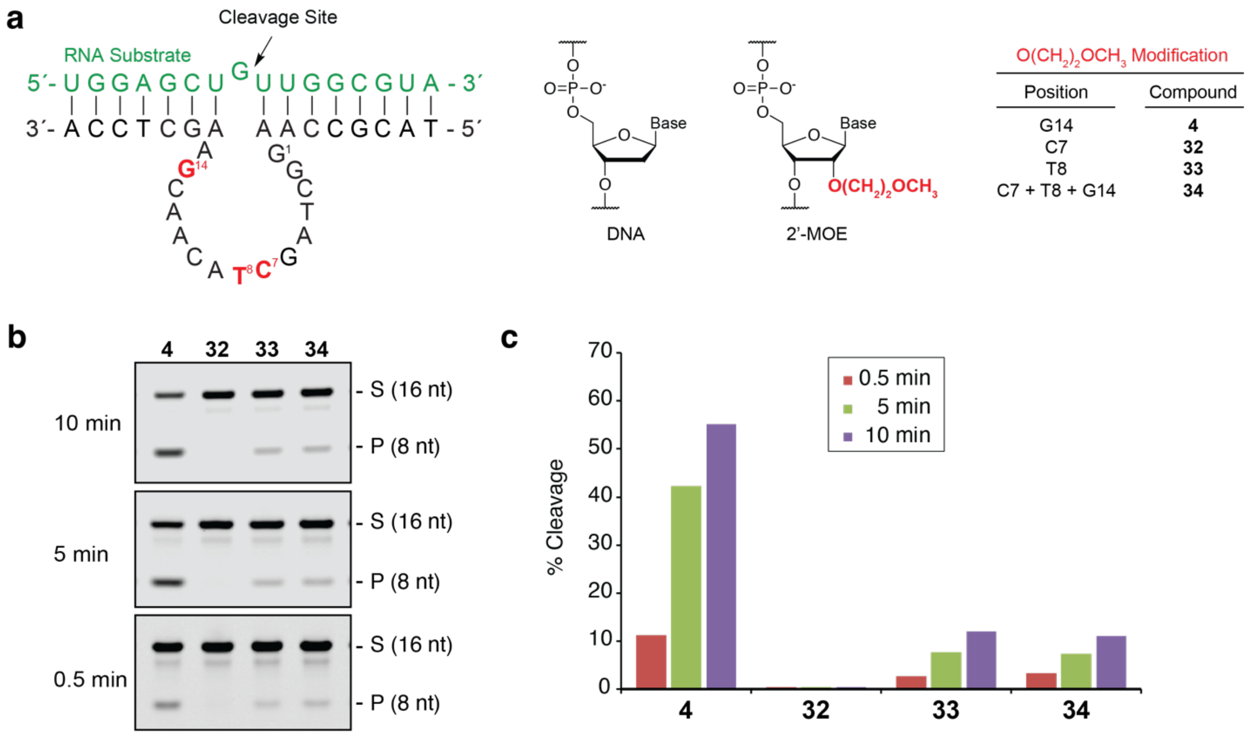

Supplementary Figure 7. Structure-activity analysis of positions 7, 8, and 14 in the catalytic loop with 2'-methoxyethoxy residues. a. Sequence of DNAzyme 10-23 targeting a KRAS G12V mRNA segment and chemical structures of sugar modified analogs. Abbreviation: MOE (2'-deoxy-2'-methoxyethoxyribonucleic acid). **b-c.** Representative gels and bar graphs showing the cleavage activity of each analog (n = 1). All reactions were performed in simulated physiological buffer containing 1 mM $MgCl_2$, 50 mM Tris (pH 7.5), 10 mM NaCl, and 140 mM KCl at 37°C with 500 nM substrate and 50 nM enzyme (10:1, S:E). S: 5'-Cy5 labeled full-length substrate, P: 5'-Cy5 labeled cleavage product. Molecular weight markers indicated to the right of the gel.

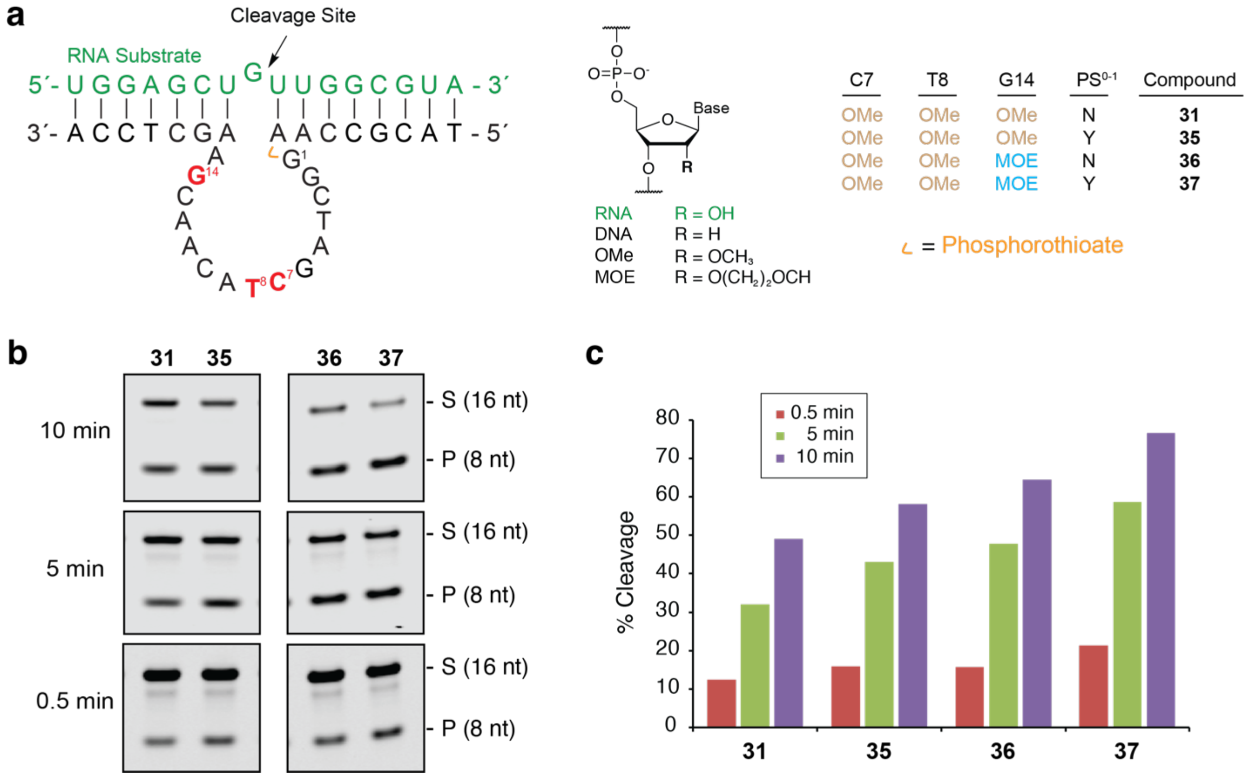

Supplementary Figure 8. Structure-activity analysis of positions 7, 8, and 14 with a phosphorothioate linkage at position 0-1 in the catalytic loop. a. Sequence of DNAzyme 10-15 targeting a KRAS G12V mRNA segment and chemical structures of sugar modified analogs. Abbreviations of nucleotide analogs: OMe (2'-deoxy-2'-methoxyribonucleic acid), MOE (2'-deoxy-2'-methoxyethoxyribonucleic acid), and phosphorothioate. **b-c.** Representative gels and bar graph showing the cleavage activity of each analog (n = 1). All reactions were performed in simulated physiological buffer containing 1 mM $MgCl_2$, 50 mM Tris (pH 7.5), 10 mM NaCl, and 140 mM KCl at 37°C with 500 nM substrate and 50 nM enzyme (10:1, S:E). S: 5'-Cy5 labeled full-length substrate, P: 5'-Cy5 labeled cleavage product. Molecular weight markers indicated to the right of the gel.

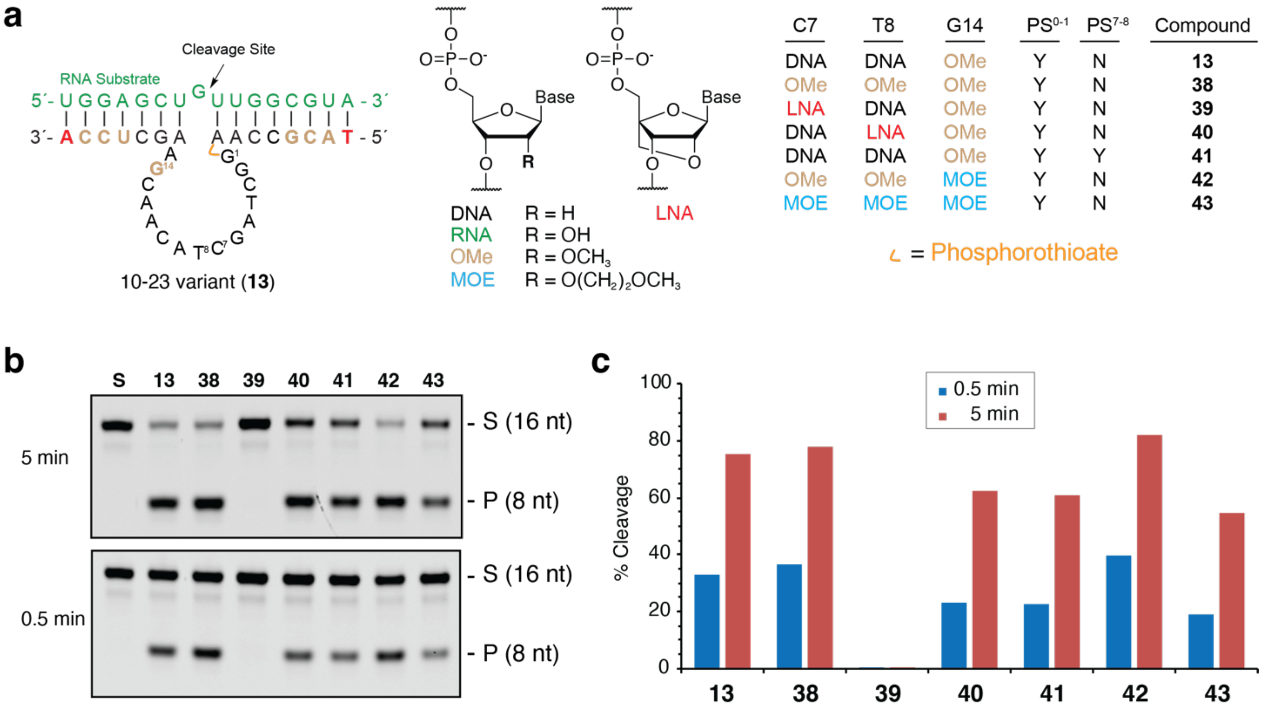

Supplementary Figure 9. Chemical optimization of 10-23 variant 13. a. Sequence of DNAzyme 10-23 targeting a segment of KRAS G12V mRNA and chemical structures of sugar modified analogs. Abbreviations of nucleotide analogs: OMe (2'-deoxy-2'-methoxyribonucleic acid), MOE (2'-deoxy-2'-methoxyethoxyribonucleic acid), LNA (locked nucleic acid), and phosphorothioate. **b-c.** Representative gel and bar graph showing the cleavage activity of each analog (n = 1). All reactions were performed in simulated physiological buffer containing 1 mM $MgCl_2$, 50 mM Tris (pH 7.5), 10 mM NaCl, and 140 mM KCl at 37°C with 500 nM substrate and 50 nM enzyme (10:1, S:E). S: 5'-Cy5 labeled full-length substrate, P: 5'-Cy5 labeled cleavage product. Molecular weight markers indicated to the right of the gel.

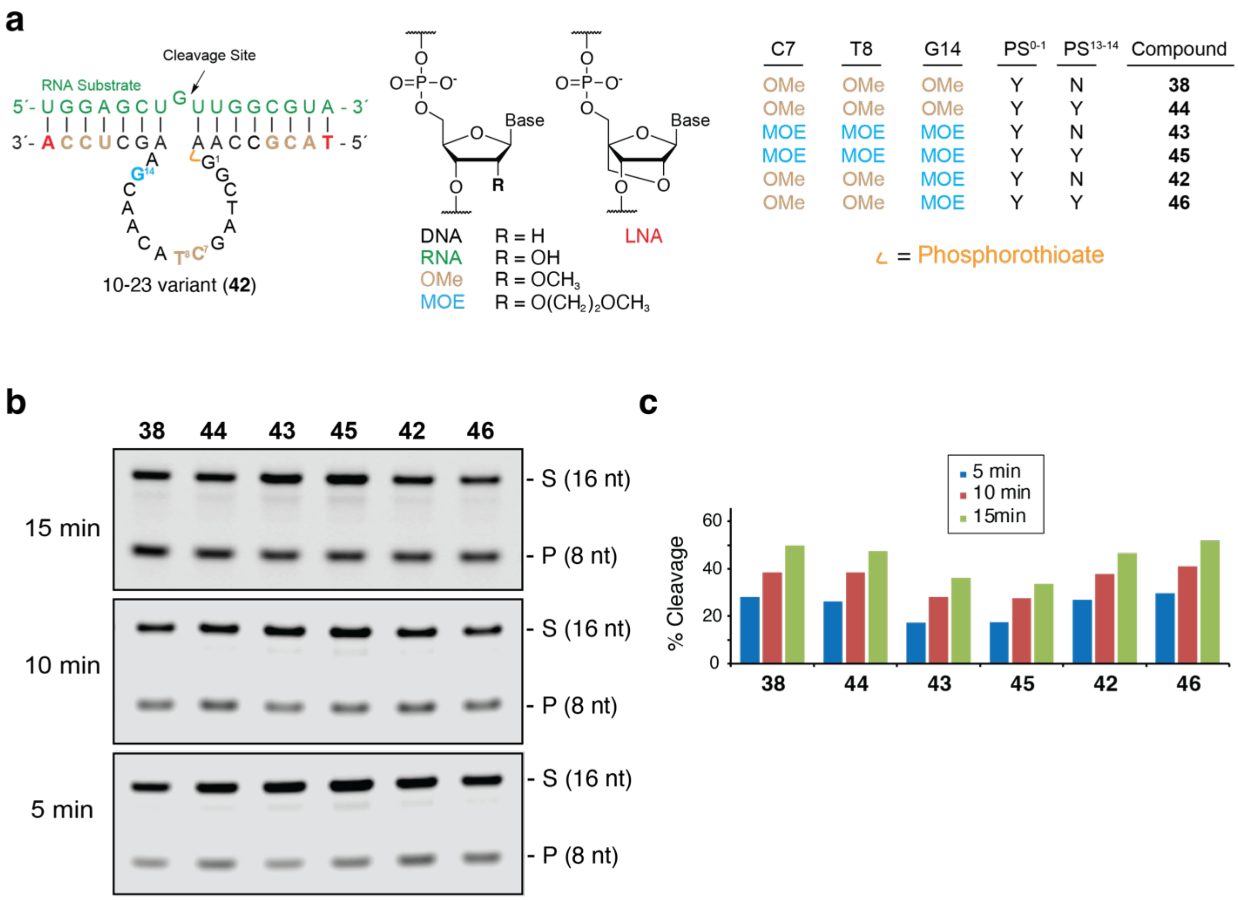

Supplementary Figure 10. Chemical optimization of 10-23 variant 42 measured at 100:1 (S:E). **a.** Sequence of DNAzyme 10-23 targeting a segment of KRAS G12V mRNA and chemical structures of sugar modified analogs. Abbreviations of nucleotide analogs: OMe (2'-deoxy-2'-methoxyribonucleic acid), MOE (2'-deoxy-2'-methoxyethoxyribonucleic acid), LNA (locked nucleic acid), and phosphorothioate. **b-c.** Representative gels and bar graphs showing the cleavage activity of each analog (n = 1). All reactions were performed in simulated physiological buffer containing 1 mM $MgCl_2$, 50 mM Tris (pH 7.5), 10 mM NaCl, and 140 mM KCl at 37°C with 1000 nM substrate and 10 nM enzyme (100:1, S:E). S: 5'-Cy5 labeled full-length substrate, P: 5'-Cy5 labeled cleavage product. Molecular weight markers indicated to the right of the gel.

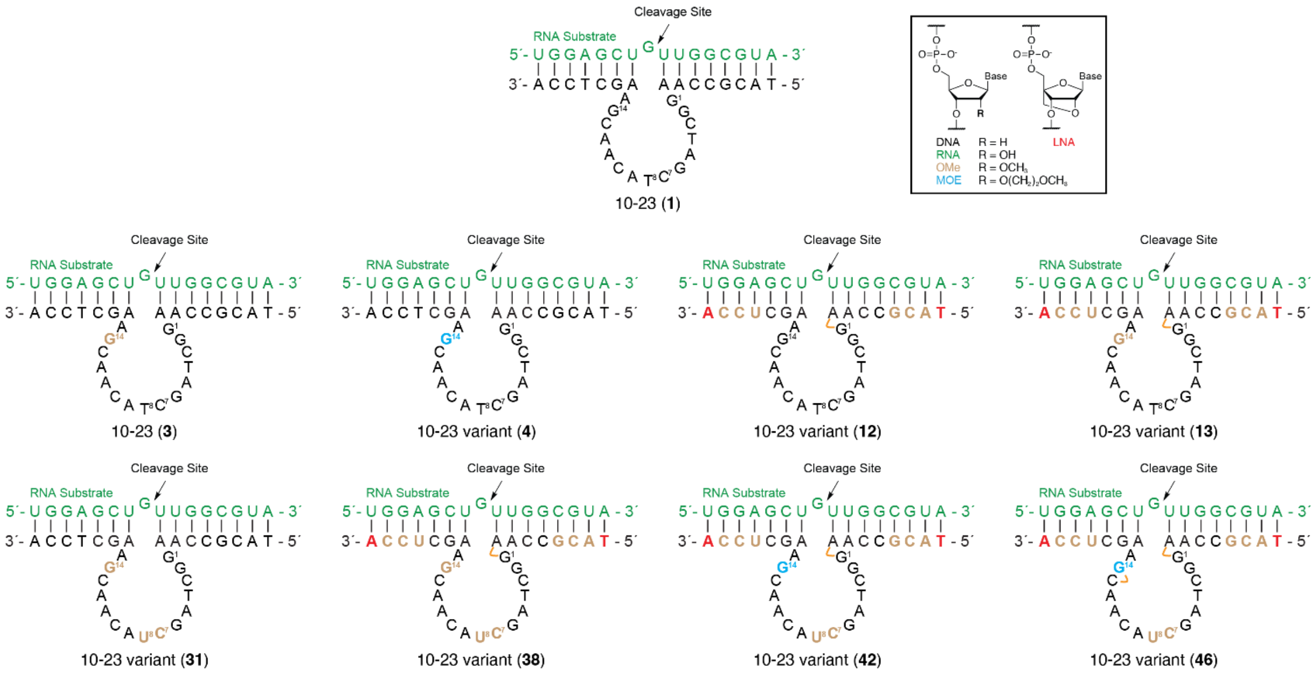

Supplementary Figure 11. Summary of 10-23 Designs Evaluated in Kinetic Cleavage Assays. Sequence of 10-23 DNAzyme variants targeting a size-matched segment of KRAS G12V mRNA and chemical structures of sugar modified analogs. Abbreviations of nucleotide analogs: OMe (2'-deoxy-2'-methoxyribonucleic acid), MOE (2'-deoxy-2'-methoxyethoxyribonucleic acid), LNA (locked nucleic acid), and phosphorothioate.

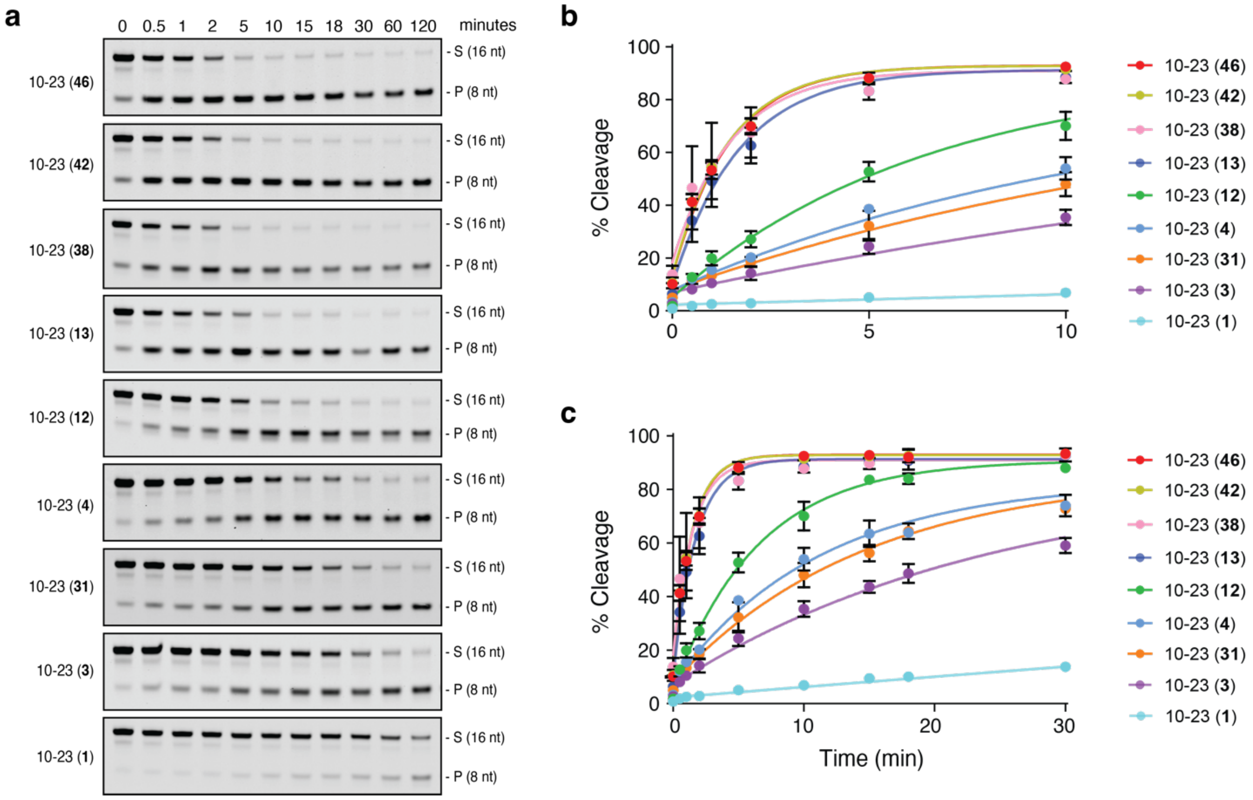

Supplementary Figure 12. Multiple turnover kinetic analysis of 10-23 variants measured at 10:1 substrate to enzyme concentration. a. Representative denaturing PAGE gels of time-dependent RNA cleavage assays. **b-c.** Kinetic curves plotted after 10 (B) and 30 (C) minutes. All reactions were performed in simulated physiological buffer containing 1 mM $MgCl_2$, 50 mM Tris (pH 7.5), 10 mM NaCl, and 140 mM KCl at 37°C with 500 nM substrate and 50 nM enzyme (10:1, S:E). S: 5'-Cy5 labeled full-length substrate, P: 5'-Cy5 labeled cleavage product. Molecular weight markers indicated to the right of the gel. Error bars denote ± standard deviation from the mean of 3 independent replicates.

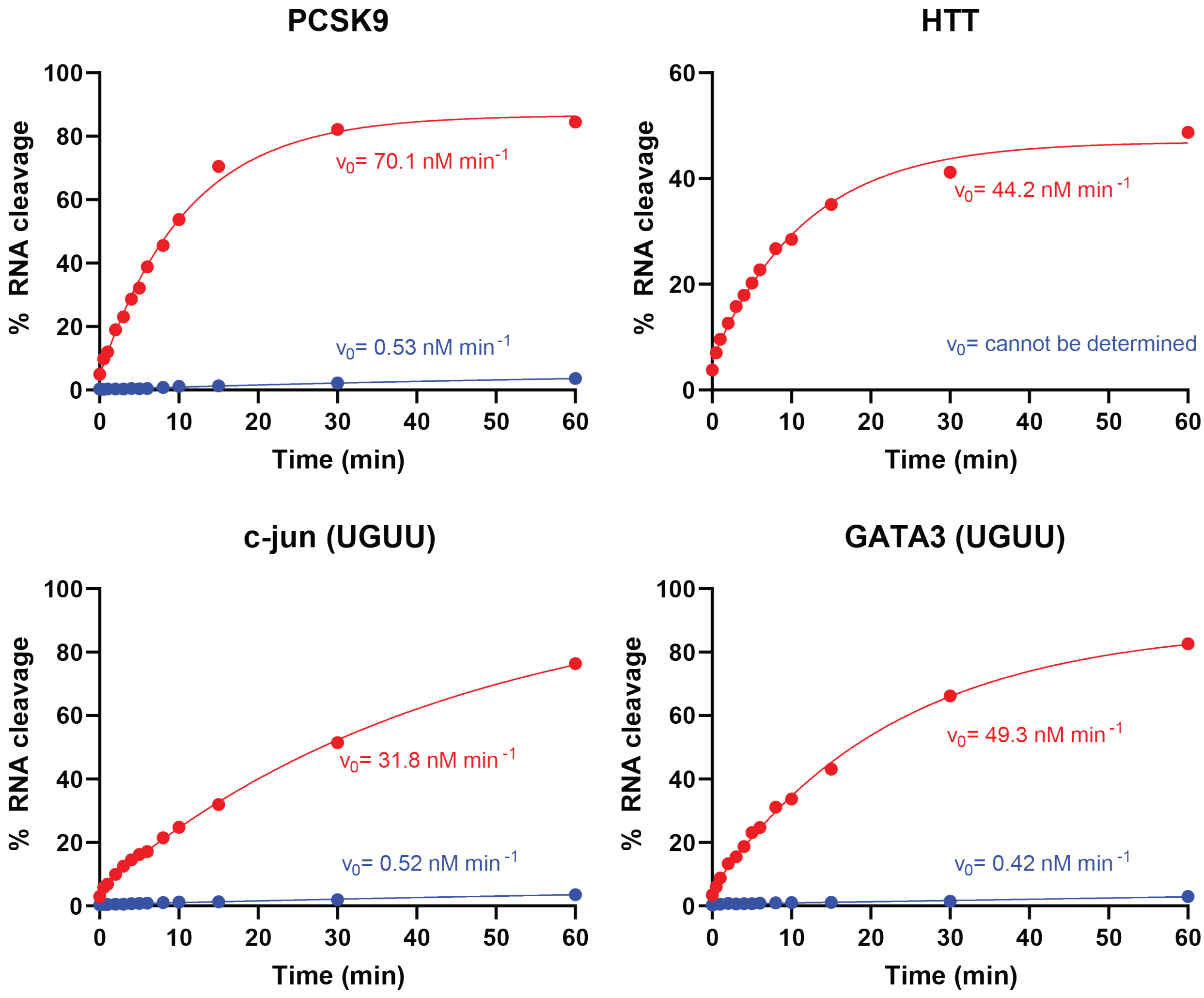

Supplementary Figure 13. Transferability of Dz 46 chemistry to other targets. Initial velocities (v_0) and kinetic curves measured for Dz 1 (blue) and Dz 46 (red) versions of PCSK9, HTT, GATA3, and c-jun sequences. All four targets utilize the UGUU cleavage motif evaluated in Figure 2c. Reported values represent the mean for 2 independent replicates. The initial velocity for Dz 1 targeting HTT could not be determined due to a lack of cleavage activity observed under the 60 min timeframe. All reactions were performed under simulated physiological conditions in buffer containing 1 mM $MgCl_2$, 50 mM Tris (pH 7.5), 10 mM NaCl, and 140 mM KCl at 37°C with 1000 nM substrate and 10 nM enzyme (100:1, S:E).

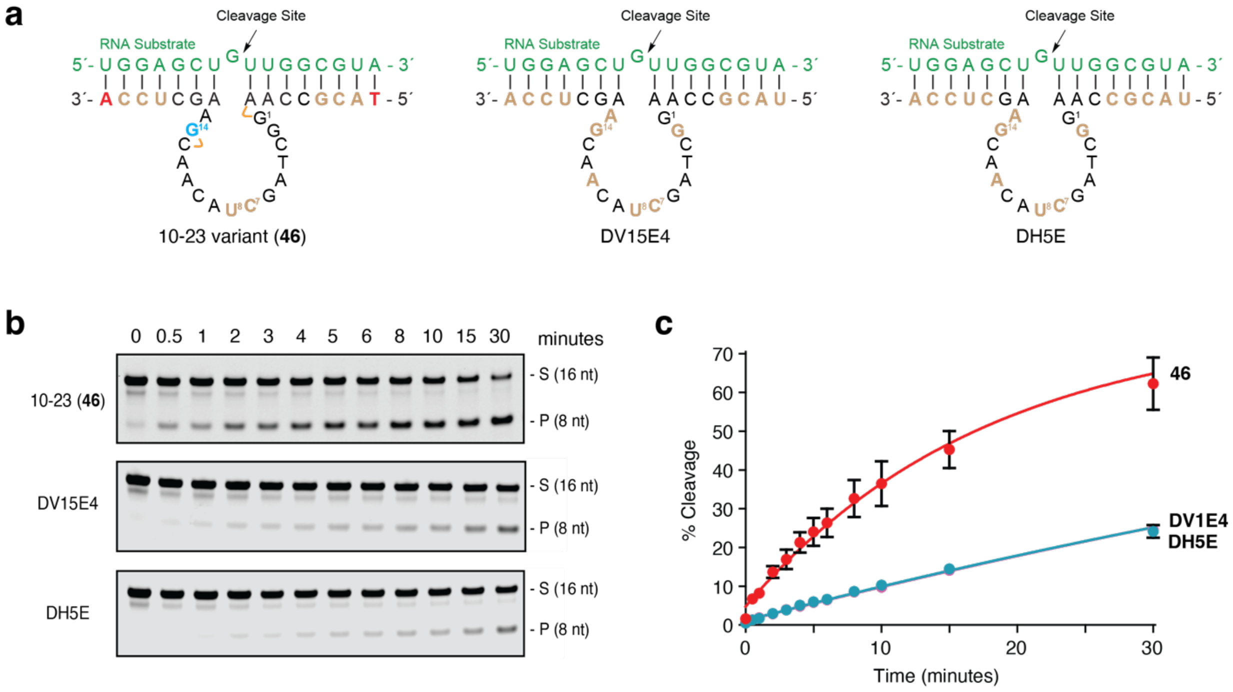

Supplementary Figure 14. Multiple turnover kinetic analysis comparing variant 46 to known methoxy derivatives at 100:1 substrate to enzyme concentration. a. Nucleic acid sequence of chemically engineered 10-23 variants targeting a segment of KRAS G12V RNA. **b.** Representative denaturing PAGE gels of time-dependent RNA cleavage assays. **c.** Kinetic curves plotted over a reaction time of 30 minutes. All reactions were performed in simulated physiological buffer containing 1 mM $MgCl_2$, 50 mM Tris (pH 7.5), 10 mM NaCl, and 140 mM KCl at 37°C with 1000 nM substrate and 10 nM enzyme (100:1, S:E). S: 5'-Cy5 labeled full-length substrate, P: 5'-Cy5 labeled cleavage product. Molecular weight markers indicated to the right of the gel. Error bars denote ± standard deviation from the mean of 3 independent replicates.

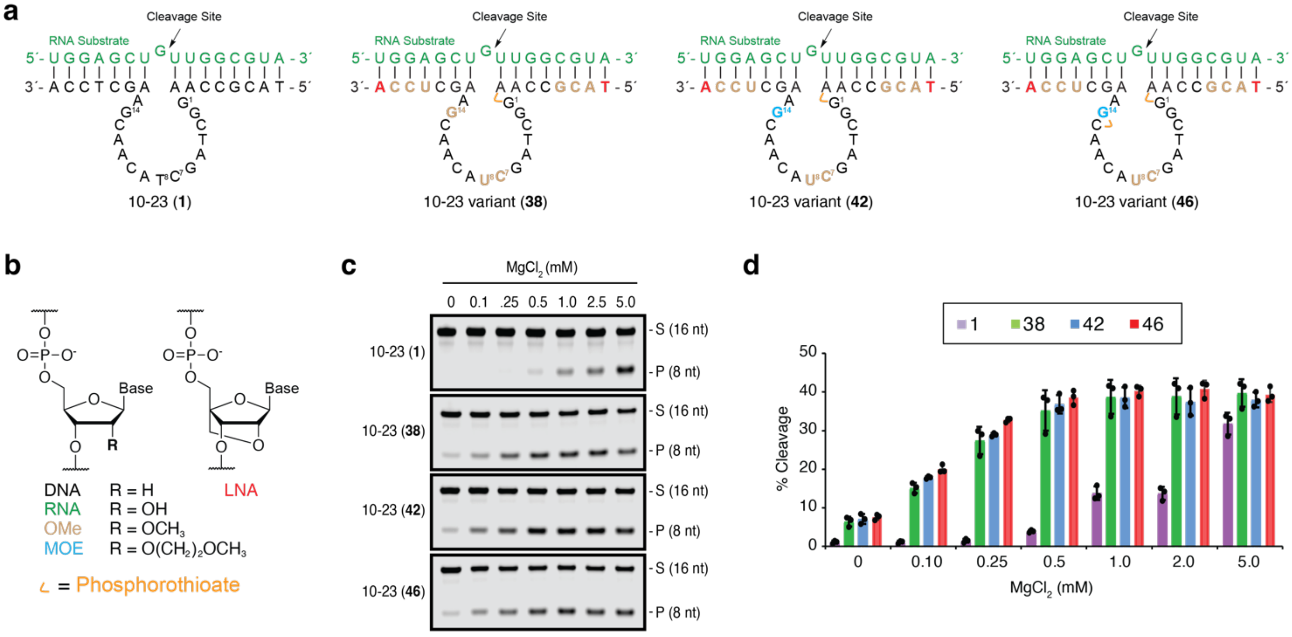

Supplementary Figure 15. Magnesium dependency of engineered 10-23 variants measured at 100:1 substrate to enzyme concentration. a. Nucleic acid sequence of 10-23 and chemically engineered 10-23 variants targeting a segment of KRAS G12V RNA substrate. **b.** Chemical structures of sugar modified analogs. Abbreviations of nucleotide analogs: OCH_3 (Ome: 2'-deoxy-2'-methoxyribonucleic acid), MOE (2'-deoxy-2'-methoxyethoxyribonucleic acid), LNA (locked nucleic acid), and phosphorothioate. **c-d.** Representative gels and bar graphs showing the cleavage activity of 10-23 variants after 20 minutes of incubation at defined concentrations of $MgCl_2$. All reactions were performed in simulated physiological buffer containing 50 mM Tris (pH 7.5), 10 mM NaCl, and 140 mM KCl at 37°C with 1000 nM substrate and 10 nM enzyme (100:1, S:E). S: 5'-Cy5 labeled full-length substrate, P: 5'-Cy5 labeled cleavage product. Molecular weight markers indicated to the right of the gel. Error bars denote ± standard deviation from the mean of 3 independent replicates.

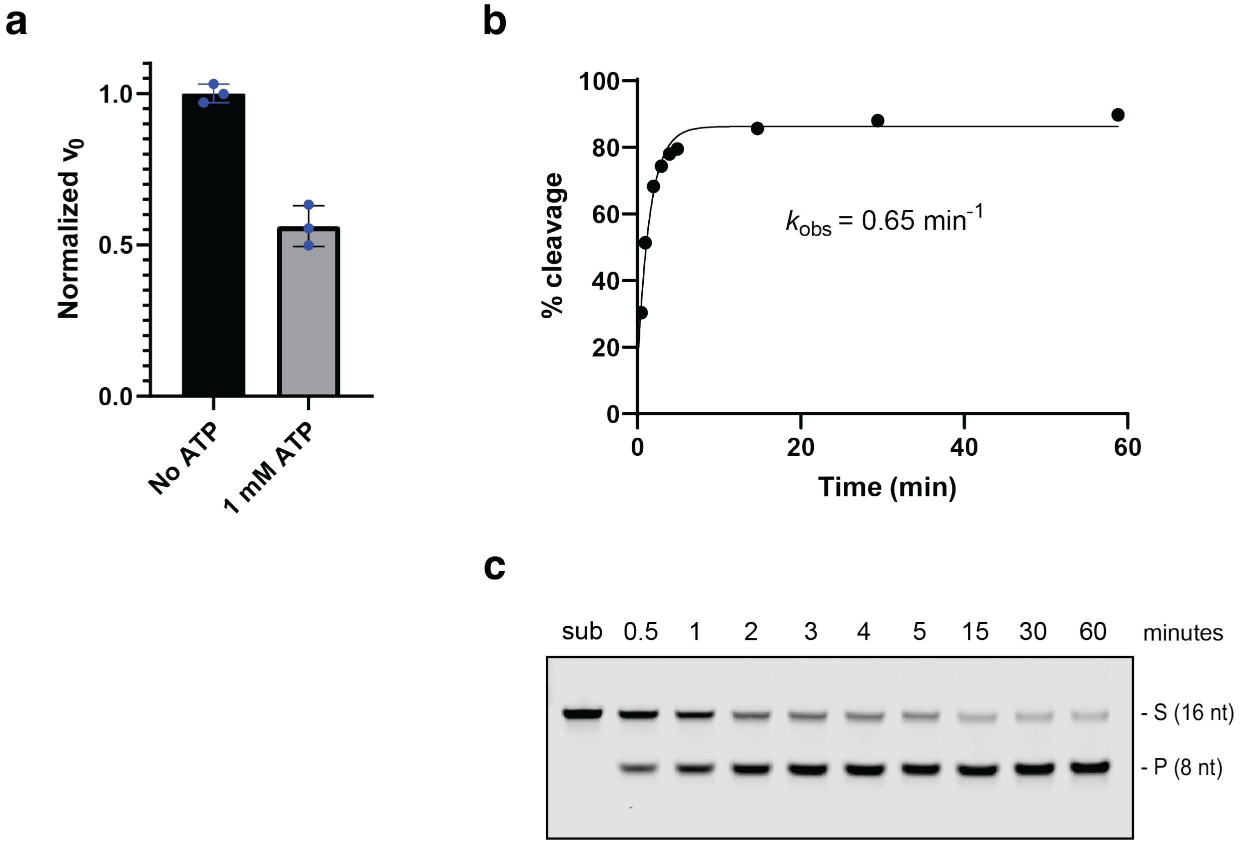

Supplementary Figure 16. Activity of Dz 46 under reduced concentrations of magnesium. a. Bar graph showing normalized initial rates for Dz 46 in the presence or absence of 1 mM ATP. Reactions were performed in simulated physiological buffer containing 1 mM $MgCl_2$, 50 mM Tris (pH 7.5), 10 mM NaCl, and 140 mM KCl at 37°C with 1000 nM substrate and 10 nM enzyme (100:1, S:E). Error bars denote ± standard deviation of the mean for 3 independent replicates (blue circles) . **b-c.** Kinetic curve and representative gel of Dz 46 activity under pseudo first-order reaction conditions. The observed rate constant (k_{obs}) is shown. Reported value represents the mean for 2 independent replicates. Reactions were performed in simulated physiological buffer containing 0.25 mM $MgCl_2$, 50 mM Tris (pH 7.5), 10 mM NaCl, and 140 mM KCl at 37°C with 0.5 μM substrate and 2.5 μM enzyme (1:5, S:E). Molecular weight markers indicated to the right of the gel. S: 5'-Cy5 labeled full-length substrate, P: 5'-Cy5 labeled cleavage product.

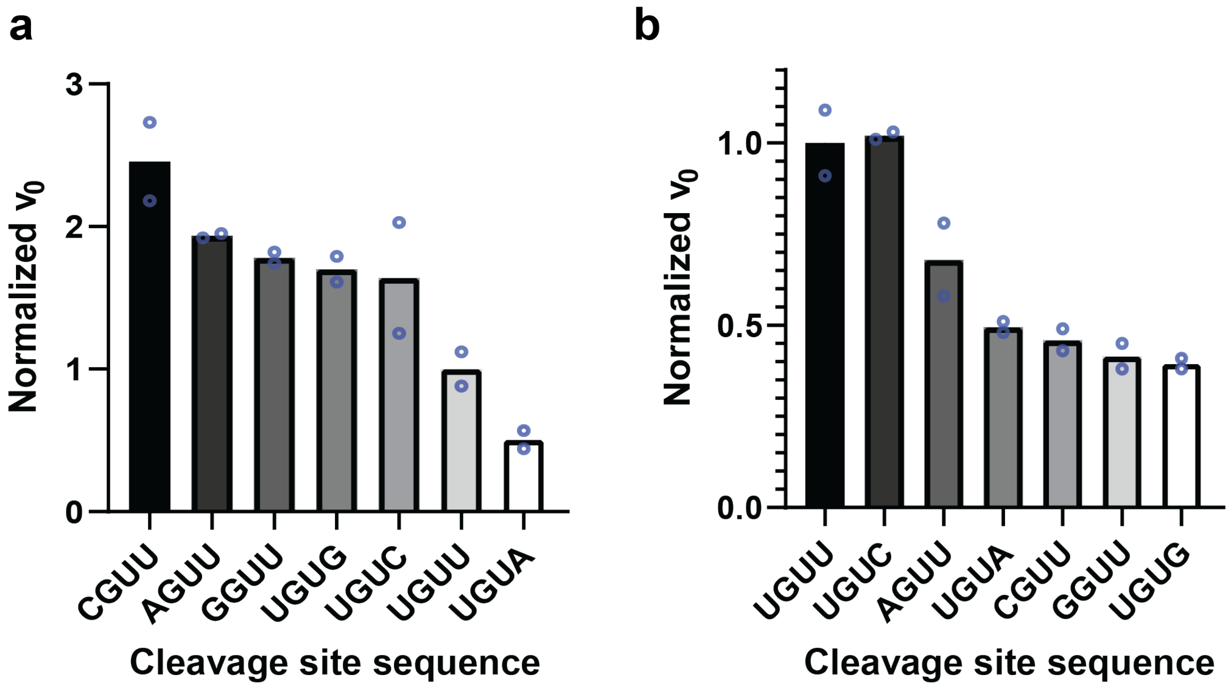

Supplementary Figure 17. Nearest neighbor analysis of unmodified Dz 1. a. Profiles observed for reactions performed in the presence of 1 mM $MgCl_2$ and **b.** 5 mM $MgCl_2$. Normalized initial rates observed for each DNAzyme-substrate pair using 1000 nM substrate and 10 nM enzyme (100:1, S:E) at 37°C in buffer containing 50 mM Tris (pH 7.5), 10 mM NaCl, 140 mM KCl. Reported values are the mean of 2 independent replicates (blue circles).

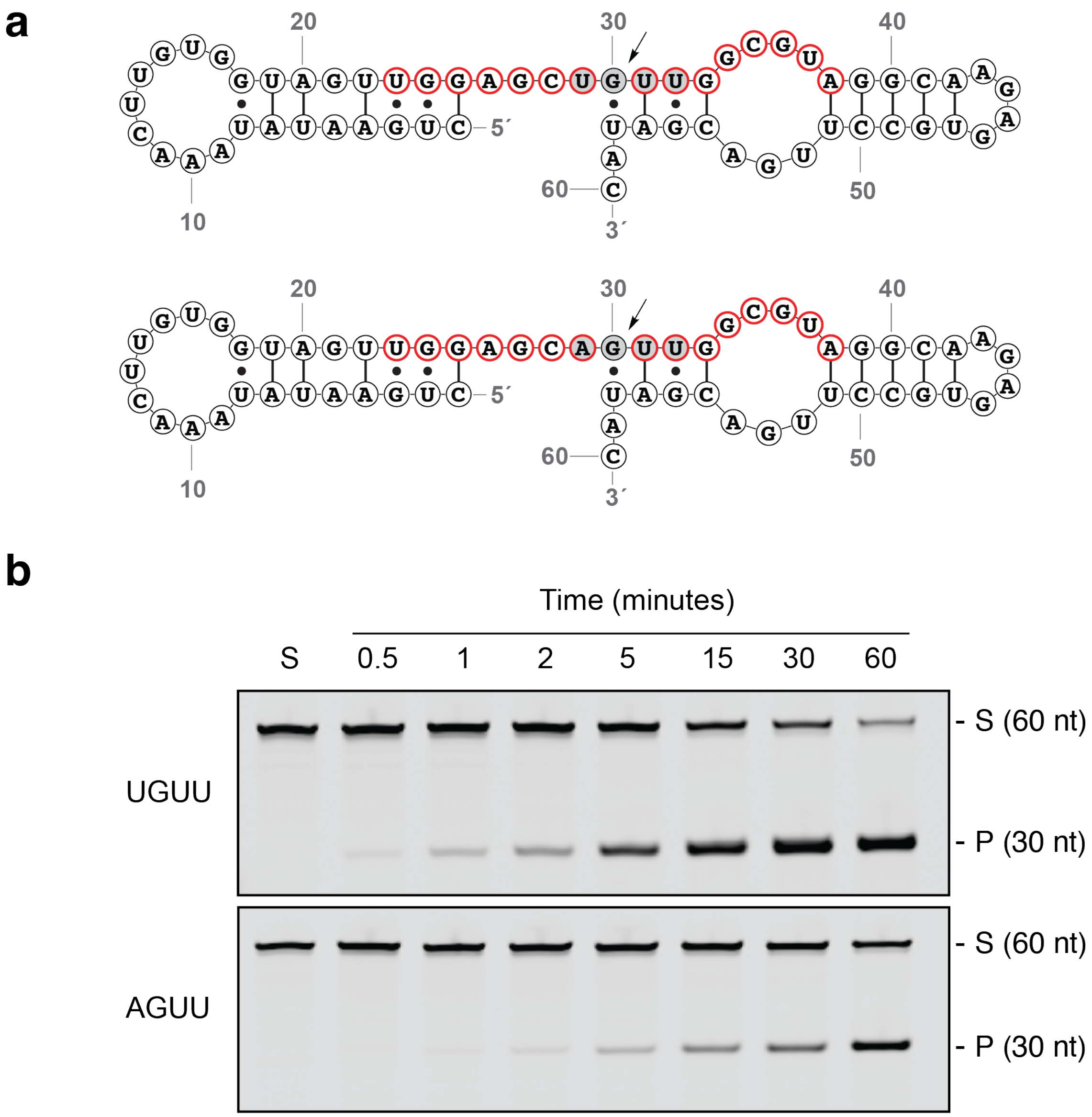

Supplementary Figure 18. Analysis of Dz 46 cleavage motif preferences in long RNA substrates. a. Predicted secondary structures of 60 nt. substrates carrying UGUU (top) or AGUU (bottom) cleavage motifs. Nucleotides complementary to the DNAzyme binding sites are outlined in red; cleavage motifs are highlighted in grey; cleavage sites are indicated by arrows. **b.** Representative PAGE gels showing time-dependent cleavage of the substrates shown above. Reactions were performed in simulated physiological buffer containing 1 mM $MgCl_2$, 50 mM Tris (pH 7.5), 10 mM NaCl, and 140 mM KCl at 37°C with 500 nM substrate and 50 nM enzyme (10:1, S:E). Molecular weight markers indicated to the right of the gel. S: 5'-Cy5 labeled full-length substrate, P: 5'-Cy5 labeled cleavage product. For each cleavage motif, n = 1.

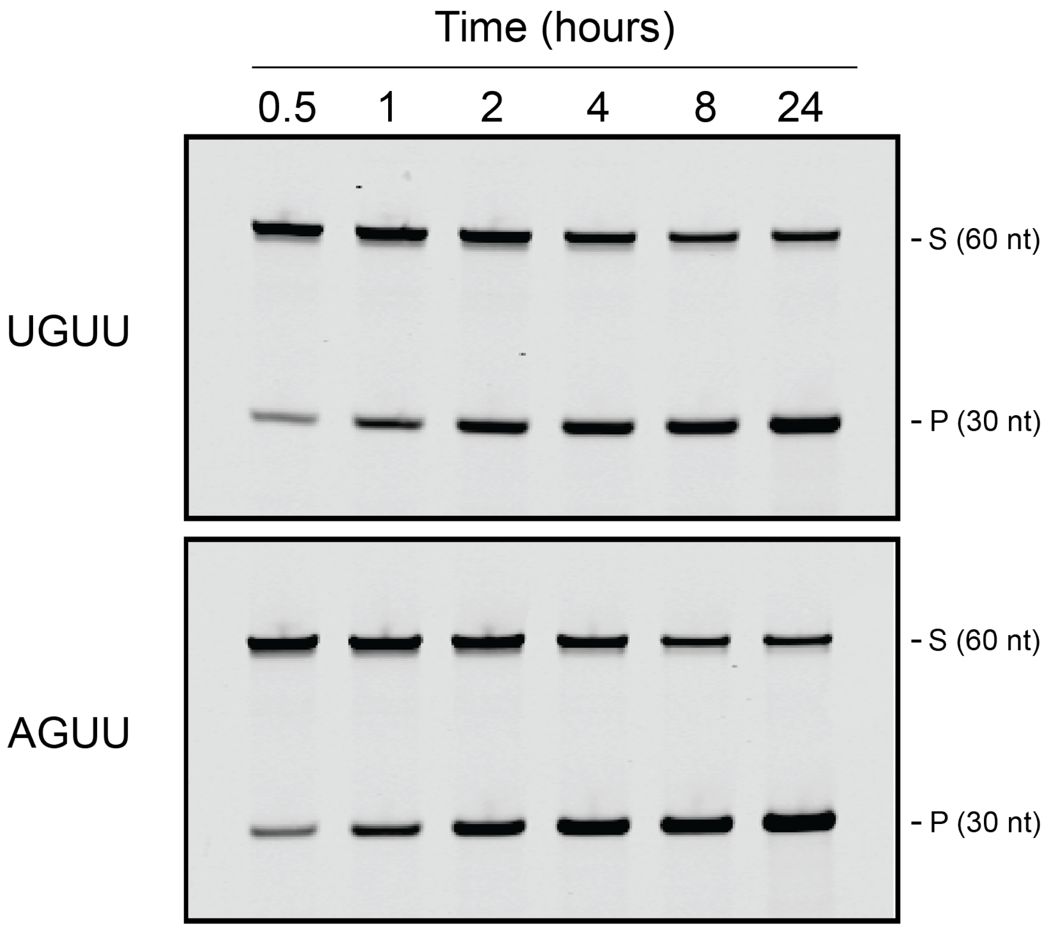

Supplementary Figure 19. Analysis of Dz 1 cleavage preferences in long RNA substrates. a. Representative PAGE gels showing time-dependent cleavage of the substrates shown in Supplementary Figure 18a. Reactions were performed in simulated physiological buffer containing 5 mM $MgCl_2$, 50 mM Tris (pH 7.5), 10 mM NaCl, and 140 mM KCl at 37°C with 500 nM substrate and 50 nM enzyme (10:1, S:E). Molecular weight markers indicated to the right of the gel. S: 5'-Cy5 labeled full-length substrate, P: 5'-Cy5 labeled cleavage product. For each cleavage motif, n = 1.

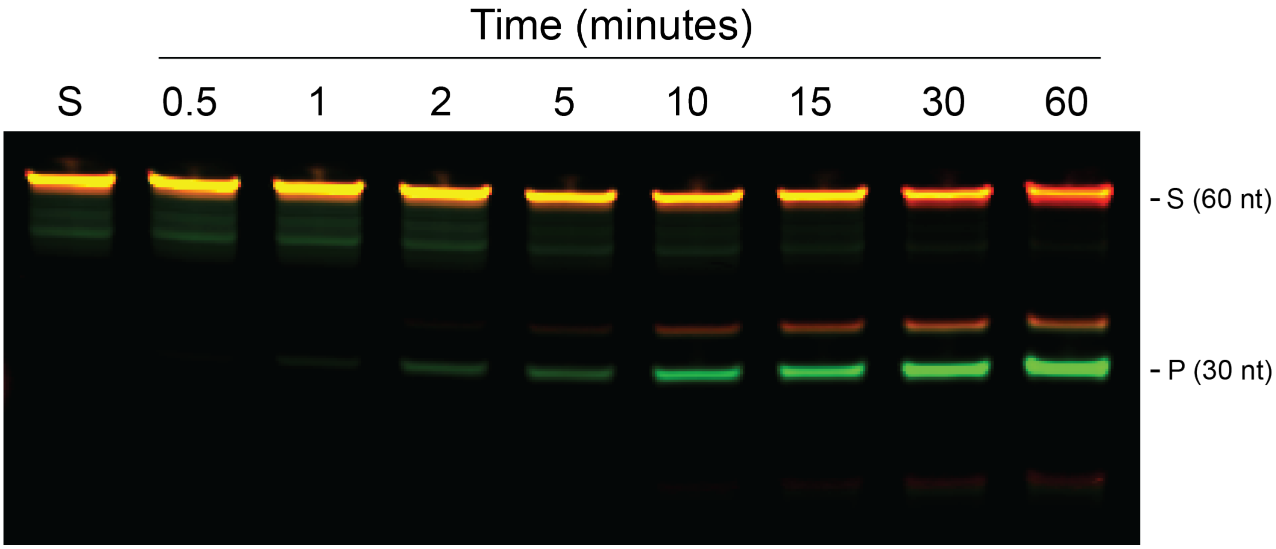

Supplementary Figure 20. Evaluation of allele-specific cleavage by Dz 46 under multiple turnover conditions in the presence of RNase H. Denaturing PAGE gels showing time-dependent RNA cleavage profiles in reactions containing both the wild-type (red) and G12V (green) substrates (1:1) in the presence of 5 ng/µL human RNase H1. Reaction was performed in a buffer containing 1 mM $MgCl_2$, 50 mM Tris (pH 7.5), 10 mM NaCl, and 140 mM KCl at 37°C with 500 nM total substrate and 50 nM enzyme (n = 1). Molecular weight markers indicated to the right of the gel. S: 5'-Cy5 labeled full-length substrate, P: 5'-Cy5 labeled cleavage product.

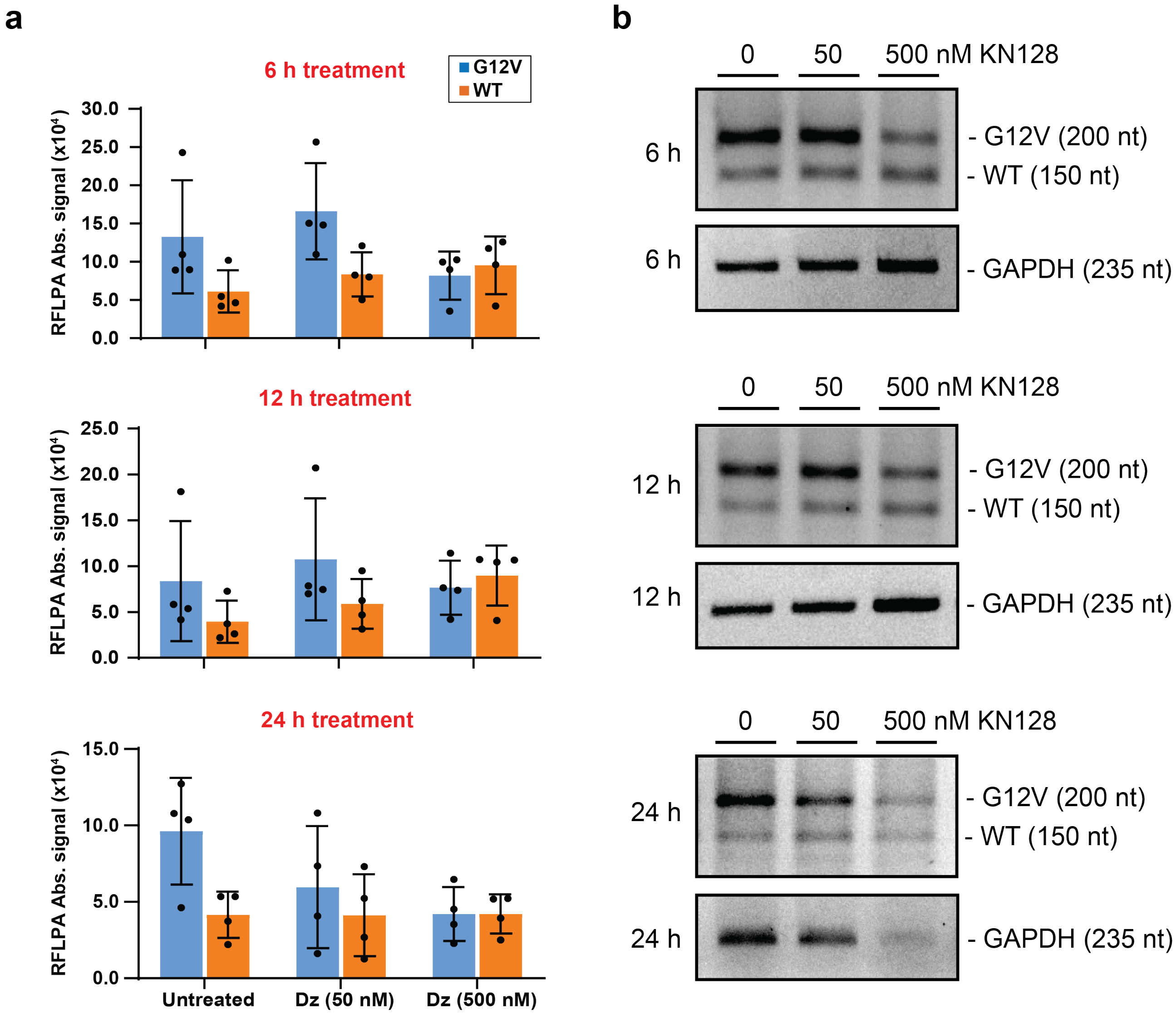

Supplementary Figure 21. Absolute values observed for allele-specific knock-down. a. Absolute signals of G12V and WT KRAS mRNA levels observed for cells treated with Dz 46 in a time course of 6h, 12h and 24h**.** Error bars denote ± standard deviation from the mean of 4 replicates (2 biological and 2 technical). **b.** Representative of agarose gel images observed following RFLPA analysis shown in Figure 5c along with a GAPDH loading control from the same cDNA (2 technical replicates). Molecular weight markers indicated to the right of the gel.

Table S1. G12V KRAS targeting DNAzymes used in chemical evolution screening

DNAzyme ID	Sequence (5' → 3'; IDT nomenclature)
1	TACGCCAA GGCTAGCTACAACGA AGCTCCA
2	TACGCCAA GGCTAGCTACAAC/i2FG/A AGCTCCA
3	TACGCCAA GGCTAGCTACAACmGA AGCTCCA
4	TACGCCAA GGCTAGCTACAAC/i2MOErG/A AGCTCCA
5	TACGCCAA GGCTAGCTACAAC+GA AGCTCCA
6	TACGCCAA GGCTAGCTACAACfGA AGCTCCA
7	TACGCCAA GGCTAGCTACAACtGA AGCTCCA
8	+T+A+CGCCAAGGCTAGCUACAACGA AGCT+C+C+A
9	+T+A+CGCCAA*GGCTAGCTACAACGA AGCT+C+C+A
10	+T+A+CGCCAA*GGCTAGCTACAACmGA AGCT+C+C+A
11	+TmAmCmGCCAA GGCTAGCTACAACGA AGCmUmCmC+A
12	+TmAmCmGCCAA*GGCTAGCTACAACGA AGCmUmCmC+A
13	+TmAmCmGCCAA*GGCTAGCTACAACmGA AGCmUmCmC+A
14	+T+A+CGCCAA*mGGCTAGCTACAACmGA AGCT+C+C+A
15	+T+A+CGCCAA*GmGCTAGCTACAACmGA AGCT+C+C+A
16	+T+A+CGCCAA*GGmCTAGCTACAACmGA AGCT+C+C+A
17	+T+A+CGCCAA*GGCmUAGCTACAACmGA AGCT+C+C+A
18	+T+A+CGCCAA*GGCTmAGCTACAACmGA AGCT+C+C+A
19	+T+A+CGCCAA*GGCTAmGCTACAACmGA AGCT+C+C+A
20	+T+A+CGCCAA*GGCTAGmCTACAACmGA AGCT+C+C+A
21	+T+A+CGCCAA*GGCTAGCmUACAACmGA AGCT+C+C+A
22	+T+A+CGCCAA*GGCTAGCTmACAACmGA AGCT+C+C+A
23	+T+A+CGCCAA*GGCTAGCTAmCAACmGA AGCT+C+C+A
24	+T+A+CGCCAA*GGCTAGCTACmAACmGA AGCT+C+C+A
25	+T+A+CGCCAA*GGCTAGCTACAmACmGA AGCT+C+C+A
26	+T+A+CGCCAA*GGCTAGCTACAAmCmGA AGCT+C+C+A
27	+T+A+CGCCAA*GGCTAGCTACAACmGmA AGCT+C+C+A

DNAzyme ID	Sequence (5' → 3'; IDT nomenclature)
28	TACGCCAA GGCTAGmCTACAACGA AGCTCCA
29	TACGCCAA GGCTAGCmUACAACGA AGCTCCA
30	TACGCCAA GGCTAGmCmUACAACGA AGCTCCA
31	TACGCCAA GGCTAGmCmUACAACmGA AGCTCCA
32	TACGCCAA GGCTAG/i2MOErC/TACAACGA AGCTCCA
33	TACGCCAA GGCTAGC/i2MOErT/ACAACGA AGCTCCA
34	TACGCCAA GGCTAG/i2MOErC//i2MOErT/ACAAC/i2MOErG/A AGCUCCA
35	TACGCCAA*GGCTAGmCmUACAACmGA AGCTCCA
36	TACGCCAA GGCTAGmCmUACAAC/i2MOErG/A AGCTCCA
37	TACGCCAA*GGCTAGmCmUACAAC/i2MOErG/A AGCTCCA
38	+TmAmCmGCCAA*GGCTAGmCmUACAACmGA AGCmUmCmC+A
39	+TmAmCmGCCAA*GGCTAG+CTACAACmGA AGCmUmCmC+A
40	+TmAmCmGCCAA*GGCTAGC+TACAACmGA AGCmUmCmC+A
41	+TmAmCmGCCAA*GGCTAGC*TACAACmGA AGCmUmCmC+A
42	+TmAmCmGCCAA*GGCTAGmCmUACAAC/i2MOErG/A AGCmUmCmC+A
43	+TmAmCmGCCAA*GGCTAG/i2MOErC//i2MOErT/ACAAC/i2MOErG/A AGCmUmCmC+A
44	+TmAmCmGCCAA*GGCTAGmCmUACAAC*mGA AGCmUmCmC+A
45	+TmAmCmGCCAA*GGCTAG/i2MOErC//i2MOErT/ACAAC*/i2MOErG/A AGCmUmCmC+A
46	+TmAmCmGCCAA*GGCTAGmCmUACAAC*/i2MOErG/A AGCmUmCmC+A

Note: LNA (+); 2’-O-methoxyribonucleic acid (m); phosphorothioate linkage (*); 2’-O-methoxyethylribonucleic acid (/i2MOE/); FANA (f); TNA (t); 2’-fluororibonucleic acid (/i2F/)

Table S2. Control oligos (active, inactive, and non-binding DNAzymes, ASO)

DNAzyme ID	Sequnce (5' → 3'; IDT nomenclature)	DNAzyme description
47	+TmAmCmGCCAA*GGC**C**A**T**mCmUAC AAC*/i2MOErG/A AGCmUmCmC+A	Dz 46 with **T4C** and **G6T** mutations in the core →**INACTIVE**
48	GCCTACGCCAACAGCTCCAACT	ASO: 22 nt DNA complementary to KRAS G12V RNA
49	+CmUmAmCGCCA*GGCTAGmCmUA CAAC*/i2MOErG/A CAGmCmUmC+C	WT KRAS-targeting DNAzyme
50	+AmGmGmUGACA*GGCTAGmCmUA CAAC*/i2MOErG/A TATmAmGmA+A	GFP-targeting DNAzyme (non-binding Dz)
DV15E4-KRAS	mUmAmCmGCCAA GmGCTAGmCmUACmAACmGmA AGCmUmCmCmA	Known motif for benchmarking
DH5E-KRAS	mUmAmCmGmCCAA GmGCTAGmCmUACmAACmGmA AGmCmUmCmCmA	Known motif for benchmarking

Note: LNA (+); 2'-O-methoxyribonucleic acid (m); phosphorothioate linkage (*); 2'-O-methoxyethylribonucleic acid (/i2MOE/)

Table S3. DNAzymes used to define the preferred cleavage motif

**DNAzyme**	**Sequence (5' → 3'; IDT nomenclature)**
DNAzyme 46 variant targeting UGU**C** RNA motif	+TmAmCmGCCGA*GGCTAGmCmUACAAC*/i2 MOErG/A AGCmUmCmC+A
DNAzyme 46 variant targeting UGU**A** RNA motif	+TmAmCmGCCTA*GGCTAGmCmUACAAC*/i2 MOErG/A AGCmUmCmC+A
DNAzyme 46 variant targeting UGU**G** RNA motif	+TmAmCmGCCCA*GGCTAGmCmUACAAC*/i2 MOErG/A AGCmUmCmC+A
DNAzyme 46 variant targeting **C**GUU RNA motif	+TmAmCmGCCAA*GGCTAGmCmUACAAC*/i2 MOErG/A GGCmUmCmC+A
DNAzyme 46 variant targeting **A**GUU RNA motif	+TmAmCmGCCAA*GGCTAGmCmUACAAC*/i2 MOErG/A TGCmUmCmC+A
DNAzyme 46 variant targeting **G**GUU RNA motif	+TmAmCmGCCAA*GGCTAGmCmUACAAC*/i2 MOErG/A CGCmUmCmC+A
GATA3-targeting Dz 46 (based on Ref 26)	+TmGmGmATGGA*GGCTAGmCmUACAAC*/i2 MOErG/A GTCmUmUmG+G
GATA3 Dz 46 variant targeting UGUU RNA motif	+TmGmGmATGAA*GGCTAGmCmUACAAC*/i2 MOErG/A ATCmUmUmG+G
c-jun-targeting Dz 46 (based on Ref 27)	+GmGmGmAGGAA*GGCTAGmCmUACAAC*/i 2MOErG/A GAGmGmCmG+T
c-jun Dz 46 variant targeting UGUU RNA motif	+GmGmGmAGGAA*GGCTAGmCmUACAAC*/i 2MOErG/A AAGmGmCmG+T
HTT-targeting Dz 46	+TmGmCmUGCAA*GGCTAGmCmUACAAC*/i2 MOErG/A AGCmUmCmC+A
HTT Dz 46 variant targeting CGUU RNA motif	+TmGmCmUGCAA*GGCTAGmCmUACAAC*/i2 MOErG/A GGCmUmCmC+A
PCSK9-targeting Dz 46	+GmCmAmCGGAA*GGCTAGmCmUACAAC*/i2 MOErG/A AAGmAmGmC+T
PCSK9 Dz 46 variant targeting AGUU RNA motif	+GmCmAmCGGAA*GGCTAGmCmUACAAC*/i2 MOErG/A TAGmAmGmC+T
PCSK9 Dz 1 variant targeting UGUU motif	GCACGGAA GGCTAGCTACAACGA AAGAGCT
HTT Dz 1 variant targeting UGUU motif	TGCTGCAA GGCTAGCTACAACGA AGCTCCA
GATA3 Dz 1 variant targeting UGUU motif	TGGATGAA GGCTAGCTACAACGA ATCTTGG

DNAzyme	Sequence (5' $\rightarrow$ 3'; IDT nomenclature)
c-jun Dz 1 variant targeting UGUU motif	GGGAGGAA GGCTAGCTACAACGA AAGGCGT

Note: LNA (+); 2’-O-methoxyribonucleic acid (m); phosphorothioate linkage (*); 2’-O-methoxyethylribonucleic acid (/i2MOE/)

Table S4. RNA substrates

RNA Substrates	Sequence (5'->3'; IDT nomenclature)
16 nt –G12V KRAS<br>(NCBI: NM_ 004985, human GTPase KRAS mRNA variant b, (positions 217-232: G225U mutation, codon 12 GGU to GUU)	/5Cy5/rUrGrGrArGrCrU**rGrU**rUrGrGrCrGrUrA
60 nt –G12V KRAS<br>(NCBI: NM_ 004985, human GTPase KRAS mRNA variant b, (positions 195-254: G225U mutation, codon 12 GGU to GUU)	/5Cy5/ rCrUrGrArArUrArUrArArArCrUrUrGrU<br>rGrGrUrArGrUrUrGrGrArGrCrU**rGrU**rUrGrGr<br>CrGrUrArGrGrCrArArGrArGrUrGrCrCrUrUrGr<br>ArCrG rArUrArC
16 nt –WT KRAS<br>(NCBI: NM_ 004985, human GTPase KRAS mRNA variant b, (positions 218-233)	/5Cy5/rGrGrArGrCrUrG**rGrU**rGrGrCrGrUrArG
60 nt –WT KRAS<br>(NCBI: NM_ 004985, human GTPase KRAS mRNA variant b, (positions 195-254)	/5Cy5/ rCrUrGrArArUrArUrArArArCrUrUrGrU<br>rGrGrUrArGrUrUrGrGrArGrCrUrG**rGrU**rGrGr<br>CrGrUrArGrGrCrArArGrArGrUrGrCrCrUrUrGr<br>ArCrG rArUrArC
60 nt- G12V KRAS (5'Alexa Fluor 750)	/5Alex750N/rCrUrGrArArUrArUrArArArCrUrUr<br>GrUrGrGrUrArGrUrUrGrGrArGrCrU**rGrU**rUrG<br>rGrCrGrUrArGrGrCrArArGrArGrUrGrCrCrUrU<br>rGrArCrGrArUrArC
16 nt - G12V KRAS substrate variant with UGUC motif	/5Cy5/rUrGrGrArGrCrU**rGrU**rCrGrGrCrGrUrA
16 nt - G12V KRAS substrate variant with UGUA motif	/5Cy5/rUrGrGrArGrCrU**rGrU**rArGrGrCrGrUrA
16 nt - G12V KRAS substrate variant with UGUG motif	/5Cy5/rUrGrGrArGrCrU**rGrU**rGrGrGrCrGrUrA
16 nt - G12V KRAS substrate variant with CGUU motif	/5Cy5/rUrGrGrArGrCrC**rGrU**rUrGrGrCrGrUrA
16 nt - G12V KRAS substrate variant with AGUU motif	5Cy5/rUrGrGrArGrCrA**rGrU**rUrGrGrCrGrUrA
16 nt - G12V KRAS substrate variant with GGUU motif	/5Cy5/rUrGrGrArGrCrG**rGrU**rUrGrGrCrGrUrA
60 nt - G12V KRAS substrate variant with AGUU motif	/5Cy5/rCrUrGrArArUrArUrArArArCrUrUrGrUr<br>GrGrUrArGrUrUrGrGrArGrCrA**rGrU**rUrGrGrC<br>rGrUrArGrGrCrArArGrArGrUrGrCrCrUrUrGrA<br>rCrGrArUrArC
16 nt - GATA3<br>NCBI: NM_001002295.2 (positions 926-941)	/5Cy5/rCrCrArArGrArC**rGrU**rCrCrArUrCrCrA

RNA Substrates	Sequence (5'->3'; IDT nomenclature)
16 nt - GATA3 substrate variant with UGUU motif	/5Cy5/rCrCrArArGrArU**rGrU**rUrCrArUrCrCrA
16 nt - c-jun<br>NCBI: NM_002228.4 (positions 1021-1036)	/5Cy5/rArCrGrCrCrUrC**rGrU**rUrCrCrUrCrCrC
16 nt - c-jun substrate variant with UGUU motif	/5Cy5/rArCrGrCrCrUrU**rGrU**rUrCrCrUrCrCrC
16 nt - HTT<br>NCBI: NM_001388492.1 (positions 1275-1290)	/5Cy5/rUrGrGrArGrCrU**rGrU**rUrGrCrArGrCrA
16 nt - HTT substrate variant with CGUU motif	/5Cy5/rUrGrGrArGrCrC**rGrU**rUrGrCrArGrCrA
16 nt - PCSK9<br>NCBI: NM_174936.4 (positions 2703-2718)	/5Cy5/rArGrCrUrCrUrU**rGrU**rUrCrCrGrUrGrC
16 nt - PCSK9 substrate variant with AGUU motif	/5Cy5/rArGrCrUrCrUrA**rGrU**rUrCrCrGrUrGrC

Note: r denotes RNA; /5Cy5/: Cy5 tag at 5’ end; /5Alex750N/: Alexa Fluor 750 tag at 5’ end; GU cleavage sites are bolded; 4 nt. cleavage motifs are underlined.

Table S5. *Hs*RNAse H1 level in human cell lines measured by ELISA

	**HEK cells**	**K562 cells**
Diameter (µm)	13.9 ± 0.13[a]	20.94 ± 1.08[b]
Cell volume (μm^3)	1405.47	4805.17
Cell volume (pL)	1.40547	4.80517
Amt *Hs*Rnase H1 / cell by ELISA (fg)	2.98	1.07
[Rnase H1] / cell (fg/pL)	2.12	0.22
[Rnase H1] / cell (ng/µL)	2.12	0.22
*Hs*RNase H1 MW (Kg/mol)	37.60	37.60
[*Hs*RNaseH1] (nM)	56.37	5.93

[a] Mateus, A, et al., (2013) Mol. Pharmaceutics, 10:2467-2478; BNID:108893
[b] Jiang, Xiaoyu et al., (2016) Magnetic Resonance in Medicine, 75:2076-1085
