# Supplementary material for: Chemical evolution of an autonomous DNAzyme with allele-specific gene silencing activity
Source: Nat Commun. 2023 Apr 27;14:2413. doi: 10.1038/s41467-023-38100-9 (PMC10140269; doi:10.1038/s41467-023-38100-9)
Supplement: Supplementary file 2 — Reporting Summary [file 41467_2023_38100_MOESM2_ESM.pdf]

nature portfolio

Corresponding author(s): John C. Chaput

Last updated by author(s): Mar 24, 2023

Reporting Summary

Nature Portfolio wishes to improve the reproducibility of the work that we publish. This form provides structure for consistency and transparency in reporting. For further information on Nature Portfolio policies, see our Editorial Policies and the Editorial Policy Checklist.

Statistics

For all statistical analyses, confirm that the following items are present in the figure legend, table legend, main text, or Methods section.

n/a	Confirmed	
☐	☒	The exact sample size (*n*) for each experimental group/condition, given as a discrete number and unit of measurement
☐	☒	A statement on whether measurements were taken from distinct samples or whether the same sample was measured repeatedly
☒	☐	The statistical test(s) used AND whether they are one- or two-sided<br>*Only common tests should be described solely by name; describe more complex techniques in the Methods section.*
☐	☒	A description of all covariates tested
☒	☐	A description of any assumptions or corrections, such as tests of normality and adjustment for multiple comparisons
☐	☒	A full description of the statistical parameters including central tendency (e.g. means) or other basic estimates (e.g. regression coefficient) AND variation (e.g. standard deviation) or associated estimates of uncertainty (e.g. confidence intervals)
☒	☐	For null hypothesis testing, the test statistic (e.g. *F*, *t*, *r*) with confidence intervals, effect sizes, degrees of freedom and *P* value noted<br>*Give P values as exact values whenever suitable.*
☒	☐	For Bayesian analysis, information on the choice of priors and Markov chain Monte Carlo settings
☒	☐	For hierarchical and complex designs, identification of the appropriate level for tests and full reporting of outcomes
☒	☐	Estimates of effect sizes (e.g. Cohen's *d*, Pearson's *r*), indicating how they were calculated

Our web collection on statistics for biologists contains articles on many of the points above.

Software and code

Policy information about availability of computer code

Data collection	No electronic data collection was performed. PAGE gels were quantified using Image Studio Lite (LI-COR) and agarose gels were quantified using GelDoc MP imaging system (Bio-Rad). ELISA assay was quantified using Clariostar Plus microplate reader (BMG Labtech).
Data analysis	Observed rate constants were obtained by fitting the percentage of cleaved substrate over the reaction time to the one-phase association equation 2 using Prism 9 (Graphpad, USA). Microsoft Excel Pro 2010 ver 14.9.4760.1000 (32-bit) and Graphpad Prism ver 9.5.0 (730) were used to generate bar graphs and analysis data from in vitro and cellular assays.

For manuscripts utilizing custom algorithms or software that are central to the research but not yet described in published literature, software must be made available to editors and reviewers. We strongly encourage code deposition in a community repository (e.g. GitHub). See the Nature Portfolio guidelines for submitting code & software for further information.

Data

Policy information about availability of data

All manuscripts must include a data availability statement. This statement should provide the following information, where applicable:

- Accession codes, unique identifiers, or web links for publicly available datasets
- A description of any restrictions on data availability
- For clinical datasets or third party data, please ensure that the statement adheres to our policy

The authors declare that the data supporting the findings of this study are available within the article and its supplementary information file. Structural information

referenced here can be found in the Protein Data Bank under accession code 7PDU. The raw data generated in this study are provided in the Supplementary Information/Source Data file

Human research participants

Policy information about studies involving human research participants and Sex and Gender in Research.

Reporting on sex and gender	N/A
Population characteristics	N/A
Recruitment	N/A
Ethics oversight	N/A

Note that full information on the approval of the study protocol must also be provided in the manuscript.

Field-specific reporting

Please select the one below that is the best fit for your research. If you are not sure, read the appropriate sections before making your selection.

- [x] Life sciences
- [ ] Behavioural & social sciences
- [ ] Ecological, evolutionary & environmental sciences

For a reference copy of the document with all sections, see nature.com/documents/nr-reporting-summary-flat.pdf

Life sciences study design

All studies must disclose on these points even when the disclosure is negative.

Sample size	No sample size calculations were performed. Samples sizes were determined using sample sizes standard in DNAzyme research (duplicate or triplicate
Data exclusions	During the revision, the data shown Figures 2c and supplementary Figures 16A (previously supplementary Figure 15A) were replaced in their entirety for consistency.
Replication	All quantitative measures presented in the main text figures were replicated in duplicate or triplicate. All attempts at replication were successful. Screening of DNAzymes performed in SI figures were not replicated, but the findings in these figures are supported by replicate data presented in the main text figures.
Randomization	Randomization was not performed. Cells transfected with or without DNAzyme were seeded at the same time using a single batch of cells of the same passage number and underwent experimentation in parallel. In each experimental replicate, the same incubator, culture plate, and batch of transfection reagents were used.
Blinding	Blinding was not used in these studies. Blinding was not necessary as all quantitative measurements were carried out unbiasedly using Gel Quantification software.

Reporting for specific materials, systems and methods

We require information from authors about some types of materials, experimental systems and methods used in many studies. Here, indicate whether each material, system or method listed is relevant to your study. If you are not sure if a list item applies to your research, read the appropriate section before selecting a response.

Materials & experimental systems

n/a	Involved in the study	
x		Antibodies
	x	Eukaryotic cell lines
x		Palaeontology and archaeology
x		Animals and other organisms
x		Clinical data
x		Dual use research of concern

Methods

n/a	Involved in the study	
x		ChIP-seq
x		Flow cytometry
x		MRI-based neuroimaging

Eukaryotic cell lines

Policy information about cell lines and Sex and Gender in Research

Cell line source(s)	HEK293, K562, and NCI-H441 cell lines were purchased from ATCC.
Authentication	The morphology of each cell lines was similar to the corresponding reference cell line at ATCC
Mycoplasma contamination	All cell lines tested negative for mycoplasma contamination
Commonly misidentified lines (See ICLAC register)	None of the cell lines used were commonly misidentified
