# Supplementary material for: Chemical evolution of an autonomous DNAzyme with allele-specific gene silencing activity
Source: Nat Commun. 2023 Apr 27;14:2413. doi: 10.1038/s41467-023-38100-9 (PMC10140269; doi:10.1038/s41467-023-38100-9)
Supplement: Supplementary file 4 — Source Data [file 41467_2023_38100_MOESM4_ESM.zip › Source Data copy/Source Data_Main Text.pdf]

Source Data for Figures 1 to 5

(Uncropped gels and tables)

Chemical Evolution of an Autonomous DNAzyme with Allele-Specific Gene Silencing Activity

Kim Nguyen1†, Turnee N. Malik1†, and John C. Chaput1-4*

1Department of Pharmaceutical Sciences, University of California, Irvine, CA 92697-3958 USA
2Department of Chemistry, University of California, Irvine, CA 92697-3958 USA
3Department of Molecular Biology and Biochemistry, University of California, CA 92697-3958 USA
4Department of Chemical and Biomolecular Engineering, University of California, Irvine, CA 92697-3958 USA

† Signifies co-first author status
* To whom correspondence should be addressed

Un-cut gels for samples from MTO (100S:1E)

Figure 1C:

Dz-1

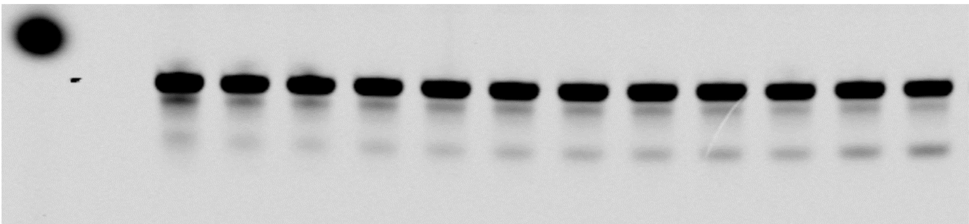

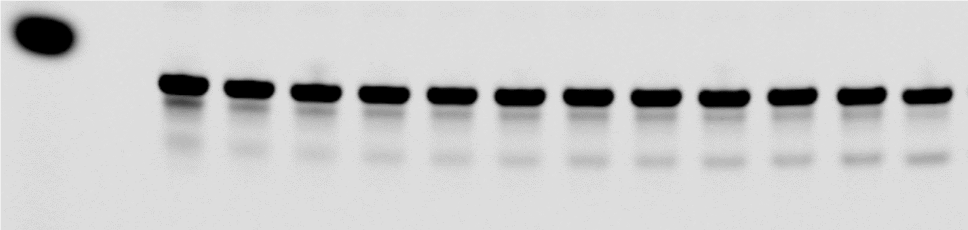

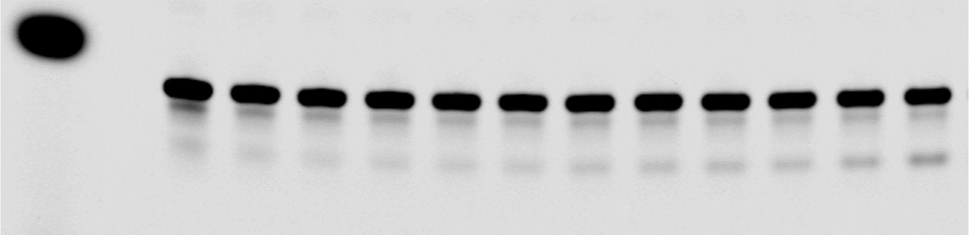

Dz-3

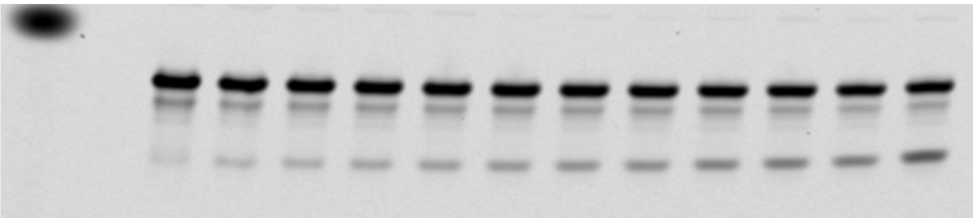

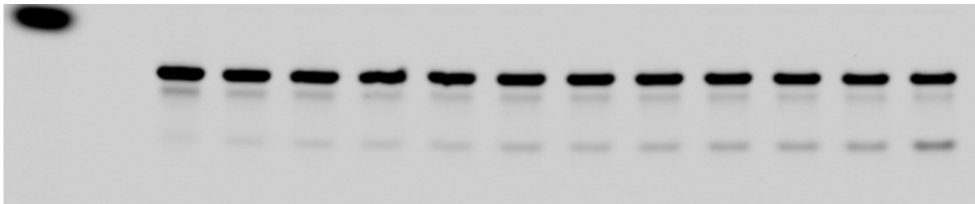

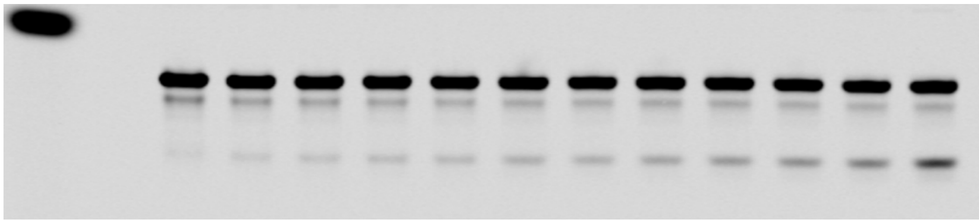

Dz-4

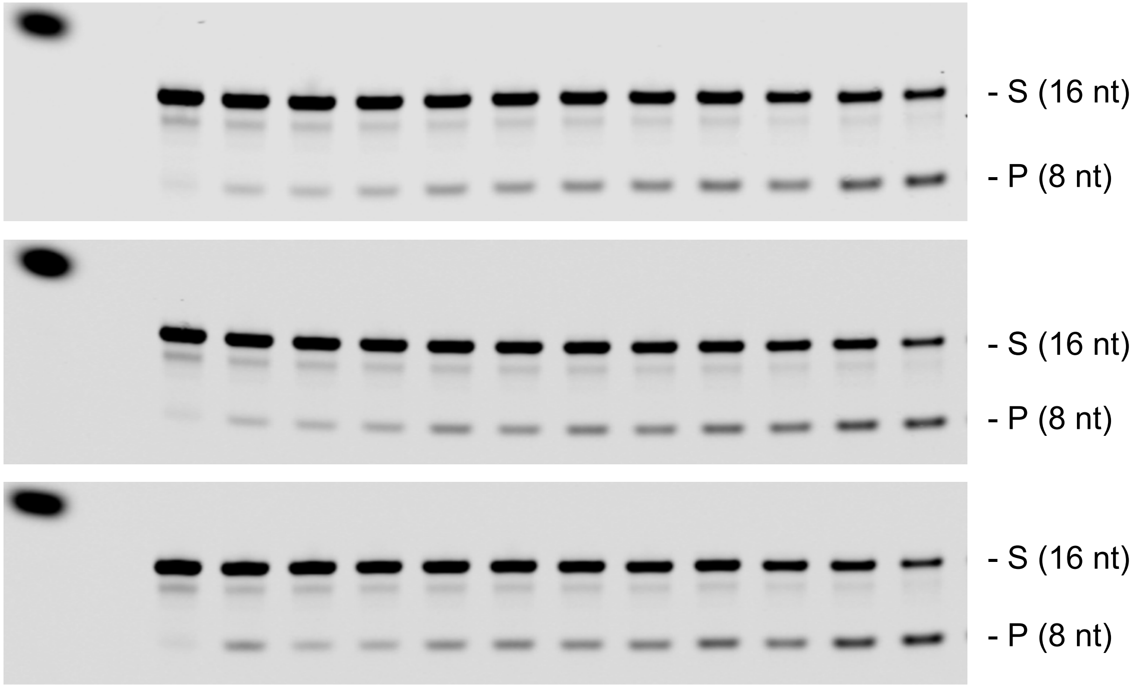
- S (16 nt)
- P (8 nt)
- S (16 nt)
- P (8 nt)
- S (16 nt)
- P (8 nt)

Dz-12

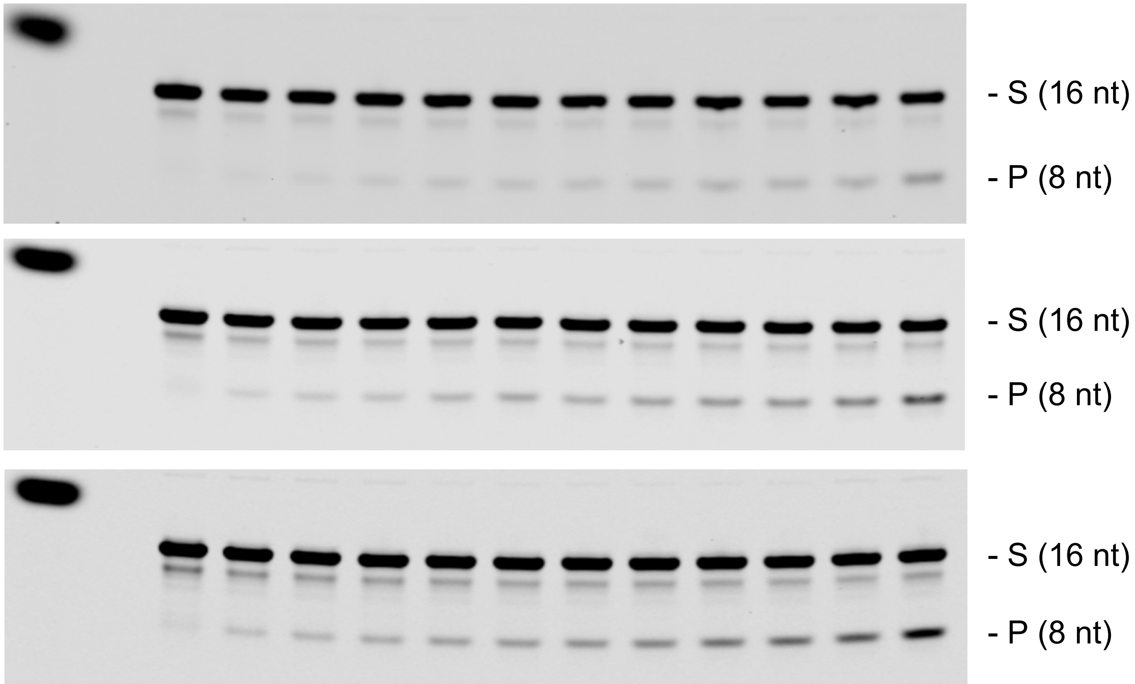
- S (16 nt)
- P (8 nt)
- S (16 nt)
- P (8 nt)
- S (16 nt)
- P (8 nt)

Dz-13

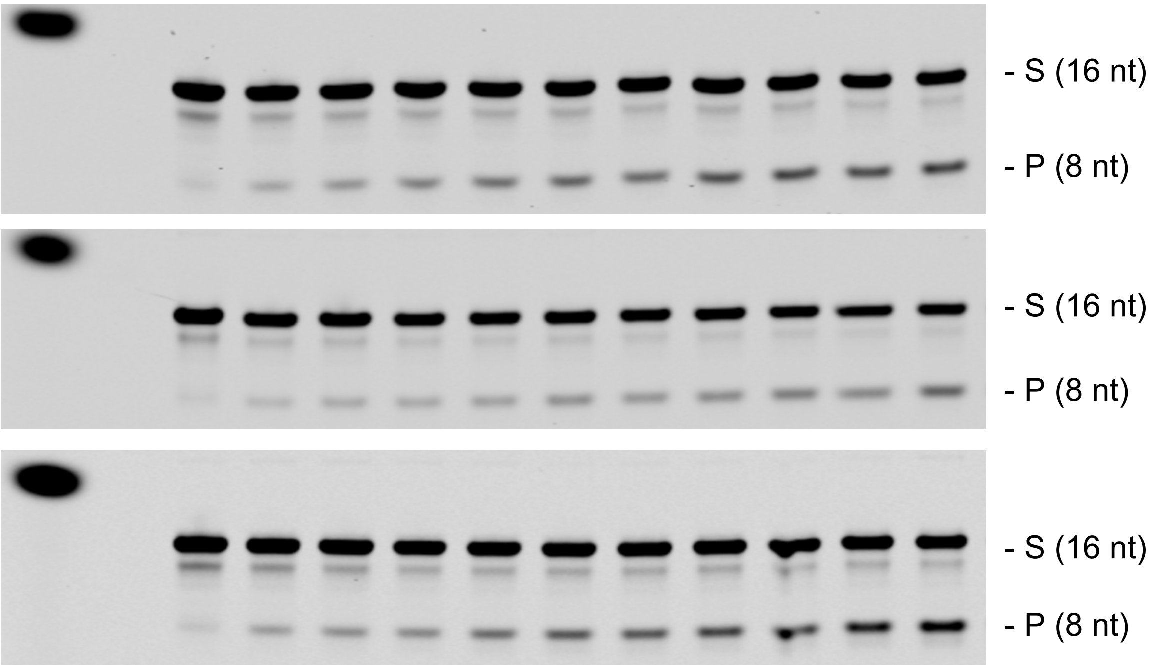
- S (16 nt)
- P (8 nt)
- S (16 nt)
- P (8 nt)
- S (16 nt)
- P (8 nt)

Dz-31

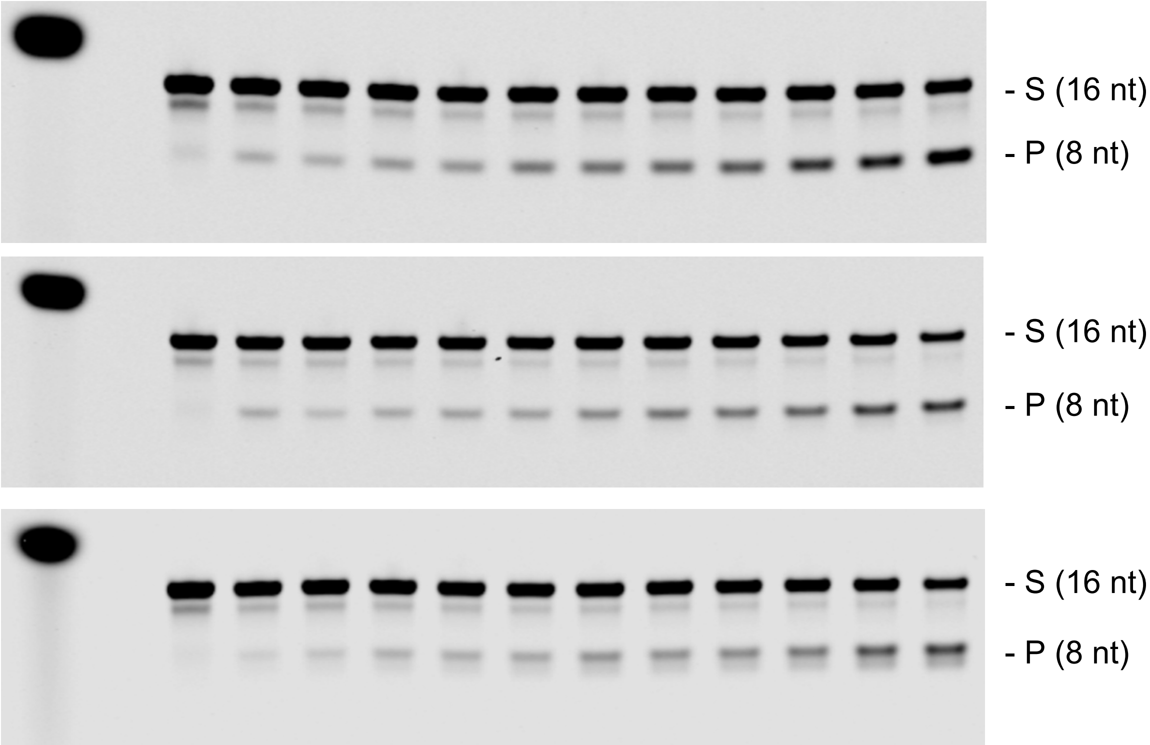
- S (16 nt)
- P (8 nt)
- S (16 nt)
- P (8 nt)
- S (16 nt)
- P (8 nt)

Dz-38

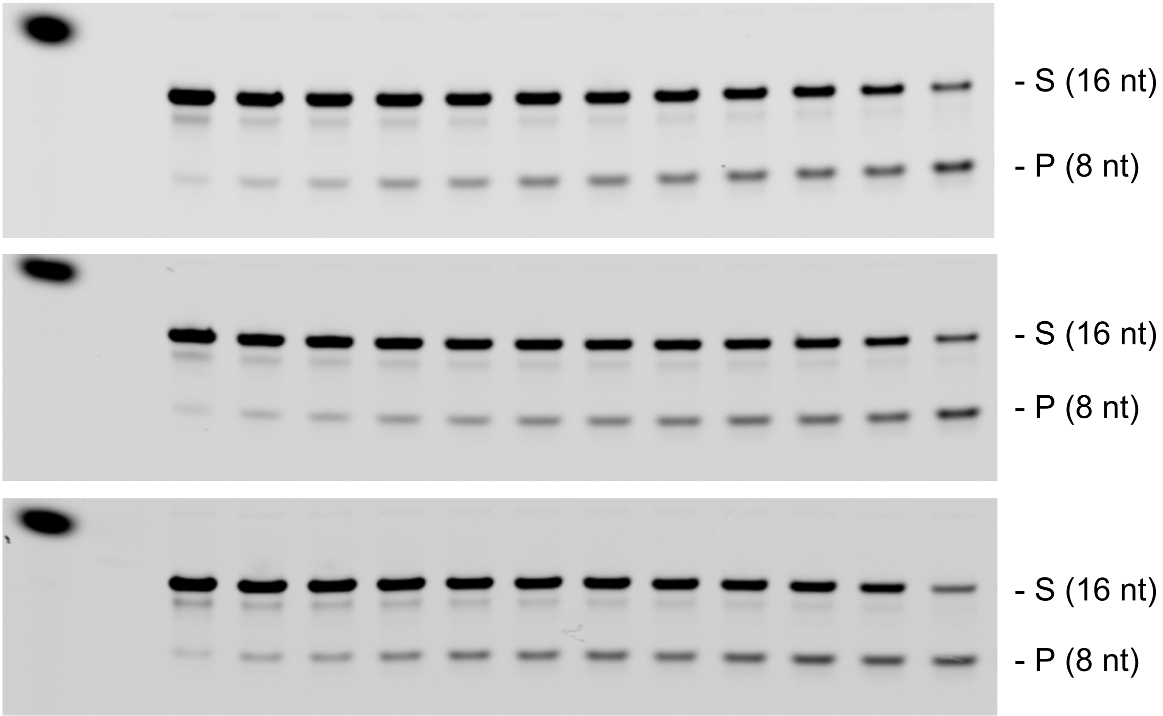

- S (16 nt)
- P (8 nt)
- S (16 nt)
- P (8 nt)
- S (16 nt)
- P (8 nt)

Dz-42

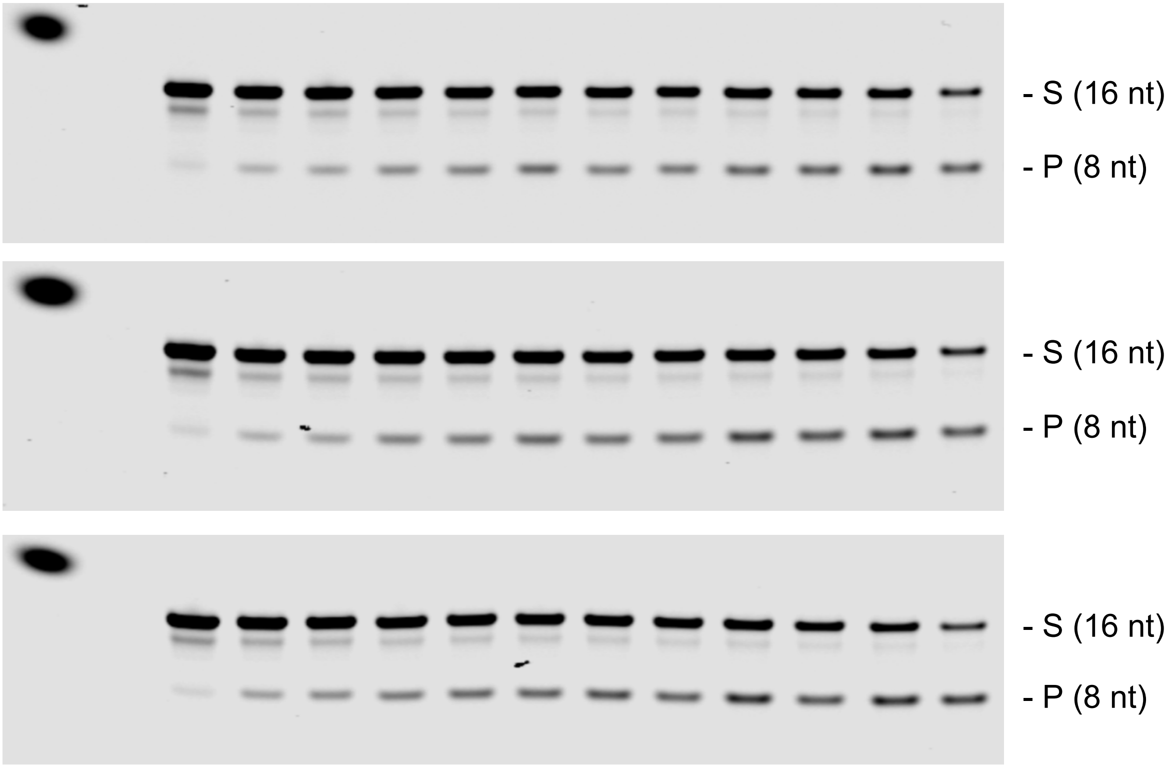

- S (16 nt)
- P (8 nt)
- S (16 nt)
- P (8 nt)
- S (16 nt)
- P (8 nt)

Dz-46

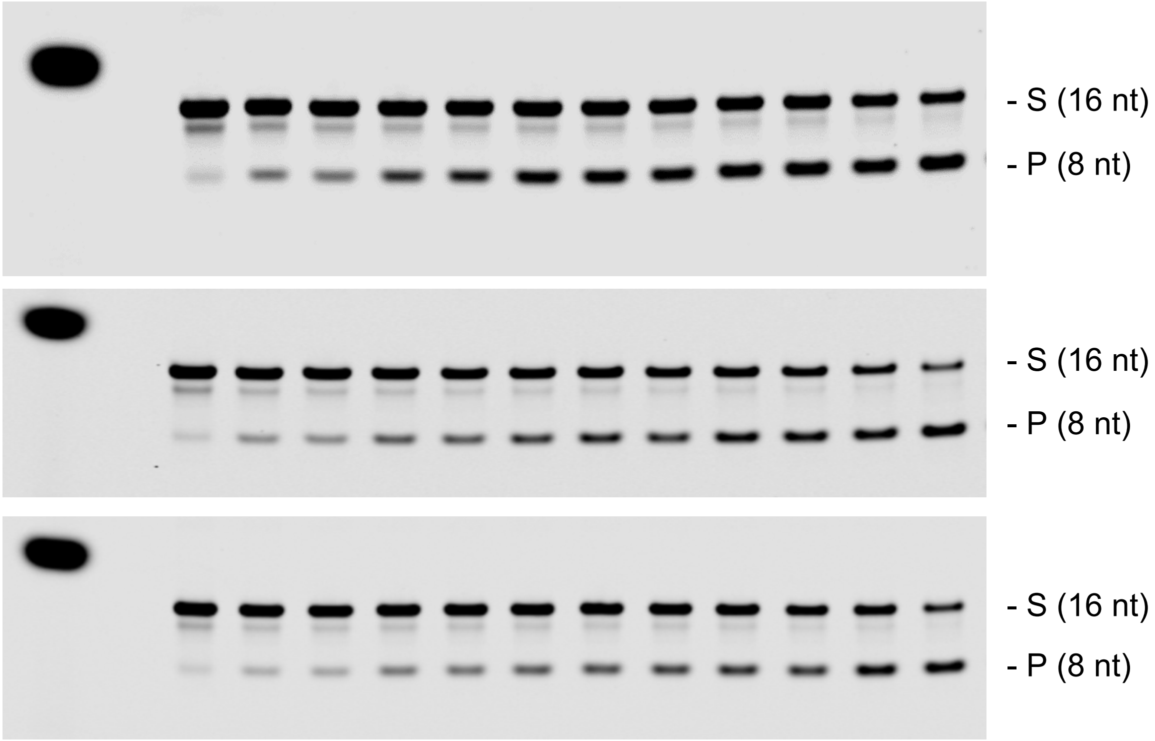

Figure 1D:

Dz #	v_o	Stdev
Dz-1	1.65	0.08
Dz-3	4.76	0.98
Dz-12	6.44	0.60
Dz-31	21.55	4.39
Dz-4	21.00	5.12
Dz-13	27.27	2.07
Dz-38	37.97	1.33
Dz-42	41.41	4.45
Dz-46	55.21	2.58

Figure 1E: MTO (100S:1E)

(100S:1E) Time (minutes)	Dz-1, r1	Dz-1, r2	Dz-1, r3	Avg
0	0.97	1.39	1.60	1.32
0.5	1.34	1.53	1.33	1.40
1	1.07	1.49	1.59	1.38
2	1.56	1.73	1.95	1.75
3	1.99	1.94	2.25	2.06
4	2.20	2.09	2.15	2.15
5	2.61	2.77	2.59	2.66
6	2.39	2.68	2.93	2.67
8	3.31	3.09	3.17	3.19
10	3.83	3.78	3.80	3.80
15	4.66	4.14	4.62	4.47
30	7.24	6.71	7.47	7.14
60	10.53	11.14	12.04	11.23
Vini	1.59	1.63	1.74	1.65
STDEV				0.08

(100S:1E) Time (minutes)	Dz-3, r1	Dz-3, r2	Dz-3, r3	Avg
0	0.53	0.55	0.40	0.49
0.5	1.61	1.07	1.28	1.32
1	2.02	1.54	1.55	1.71
2	2.69	2.24	2.21	2.38
3	3.36	2.72	2.62	2.90
4	3.76	3.46	3.16	3.46
5	4.46	3.30	3.64	3.80
6	4.91	4.00	3.73	4.21
8	6.39	5.10	5.12	5.54
10	7.90	6.04	6.29	6.74
15	9.36	7.33	7.87	8.19
30	16.67	13.50	12.66	14.28
Vini	5.89	4.32	4.09	4.76
STDEV				0.98

(100S:1E) Time (minutes)	Dz-12, r1	Dz-12, r2	Dz-12, r3	Avg
0	0.31	0.48	0.48	0.42
0.5	0.91	1.17	1.53	1.20
1	1.56	1.73	2.22	1.84
2	2.48	2.81	2.74	2.67
3	3.38	3.70	3.90	3.66
4	4.01	4.75	4.61	4.45
5	4.25	5.17	5.00	4.81
6	5.02	6.32	5.82	5.72
8	6.43	7.44	7.07	6.98
10	7.85	8.42	8.79	8.36
15	8.98	10.87	10.42	10.09
30	15.94	17.88	15.99	16.61
Vini	5.78	6.93	6.63	6.44
STDEV				0.60

(100S:1E) Time (minutes)	Dz-4, r1	Dz-4, r2	Dz-4, r3	Avg
0	0.96	0.93	0.95	0.95
0.5	3.46	3.74	8.28	5.16
1	4.14	4.59	4.77	4.50
2	6.45	6.84	6.65	6.65
3	9.90	10.78	10.59	10.42
4	10.71	10.85	12.84	11.47
5	13.21	14.09	13.24	13.51
6	15.85	15.96	17.02	16.28
8	18.64	19.77	20.86	19.76
10	22.60	23.81	23.93	23.45
15	31.55	31.01	34.77	32.44
30	43.70	46.95	51.16	47.27
Vini	24.50	26.31	24.58	25.13
STDEV				1.03

(100S:1E) Time (minutes)	Dz-31, r1	Dz-31, r2	Dz-31, r3	Avg
0	0.79	0.54	0.52	0.62
0.5	3.44	4.82	1.78	3.35
1	4.06	3.67	2.88	3.54
2	6.33	6.40	5.12	5.95
3	8.14	7.98	6.03	7.38
4	10.98	9.58	7.03	9.20
5	11.78	11.72	9.61	11.04
6	13.84	13.67	10.60	12.70
8	18.75	17.18	13.86	16.59
10	20.41	22.47	17.19	20.02
15	27.05	29.82	24.50	27.12
30	42.00	41.95	36.63	40.19
Vini	25.48	22.35	16.81	21.55
STDEV				4.39

(100S:1E) Time (minutes)	Dz-13, r1	Dz-13, r2	Dz-13, r3	Avg
0	0.67	0.82	0.55	0.68
0.5	2.92	3.31	2.79	3.01
1	4.49	5.26	4.38	4.71
2	6.59	6.30	6.06	6.32
3	8.73	8.88	8.20	8.60
4	9.80	10.45	9.55	9.93
5	10.95	10.87	10.41	10.74
6	12.49	12.32	11.75	12.19
8	14.69	16.56	14.46	15.23
10	17.54	18.53	17.10	17.72
15	20.40	23.08	23.96	22.48
30	34.25	33.79	35.10	34.38
Vini	20.56	20.11	19.73	20.13
STDEV				0.41

(100S:1E) Time (minutes)	Dz-42, r1	Dz-42, r2	Dz-42, r3	Avg
0	0.96	0.85	1.36	1.06
0.5	3.99	4.28	5.29	4.52
1	6.07	12.42	7.70	8.73
2	9.62	9.81	11.72	10.38
3	12.98	12.18	15.27	13.48
4	16.58	15.17	17.54	16.43
5	16.40	17.08	20.97	18.15
6	18.34	19.85	21.40	19.86
8	21.84	24.25	28.48	24.86
10	25.71	25.16	29.78	26.88
15	32.89	32.98	38.97	34.95
30	44.76	45.50	53.30	47.85
Vini	40.06	37.79	46.37	41.41
STDEV				4.45

(100S:1E) Time (minutes)	Dz-38, r1	Dz-38, r2	Dz-38, r3	Avg
0	1.57	1.57	1.72	1.62
0.5	4.49	4.63	5.03	4.72
1	6.32	6.18	6.55	6.35
2	10.03	9.30	9.74	9.69
3	12.61	12.88	13.54	13.01
4	16.04	15.63	15.51	15.73
5	18.40	18.33	18.69	18.47
6	21.44	20.67	20.36	20.82
8	25.62	24.22	23.89	24.58
10	28.22	27.13	28.78	28.04
15	37.05	36.69	34.78	36.18
30	59.21	58.06	56.87	58.05
Vini	36.80	37.69	39.42	37.97
STDEV				1.33

(100S:1E) Time (minutes)	Dz-46, r1	Dz 46, r2	Dz-46, r3	Avg
0	2.47	2.24	2.84	2.52
0.5	6.26	6.51	6.45	6.41
1	8.29	8.83	8.90	8.68
2	13.36	13.86	13.46	13.56
3	17.70	17.62	18.90	18.07
4	21.63	21.72	22.75	22.03
5	24.38	24.48	24.74	24.53
6	26.87	27.10	27.97	27.31
8	33.25	32.99	34.01	33.42
10	39.14	39.48	40.76	39.79
15	49.36	49.43	48.69	49.16
30	64.96	66.44	69.50	66.97
Vini	54.42	58.09	53.11	55.21
STDEV				2.58

Figure 2B

UGUU

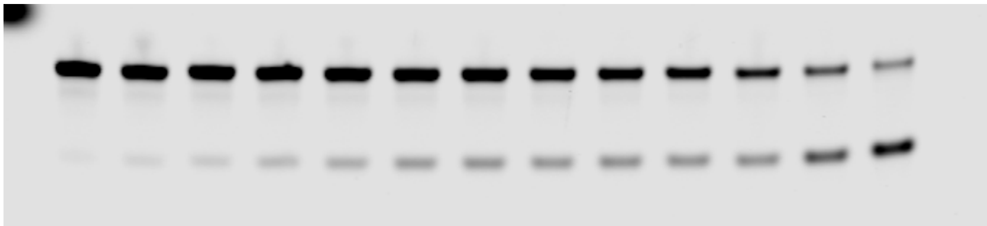

- S (16 nt)
- P (8 nt)

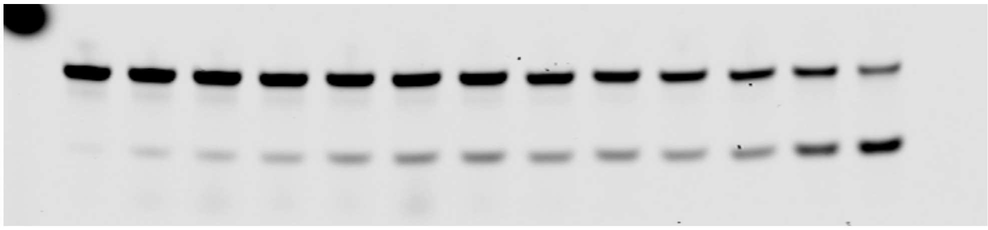

- S (16 nt)
- P (8 nt)

UGUG

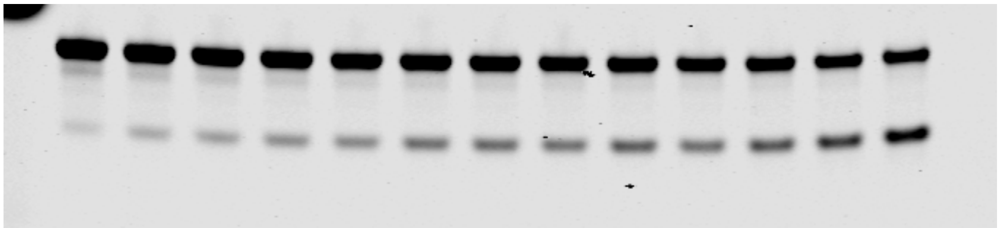

- S (16 nt)
- P (8 nt)

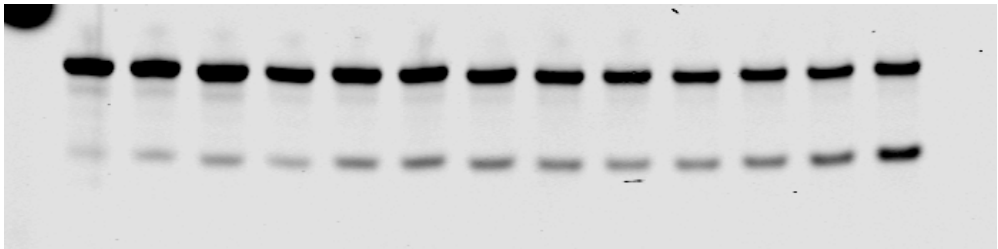

- S (16 nt)
- P (8 nt)

UGUC

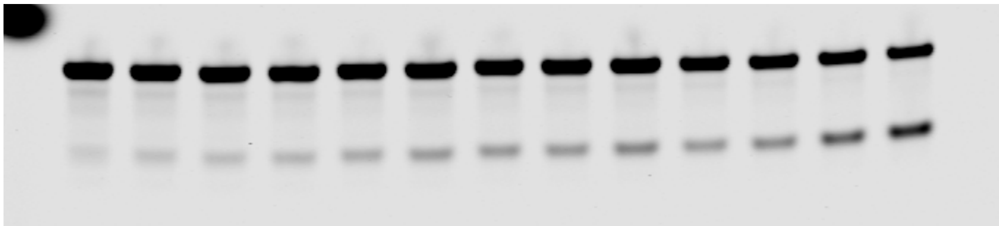

- S (16 nt)
- P (8 nt)

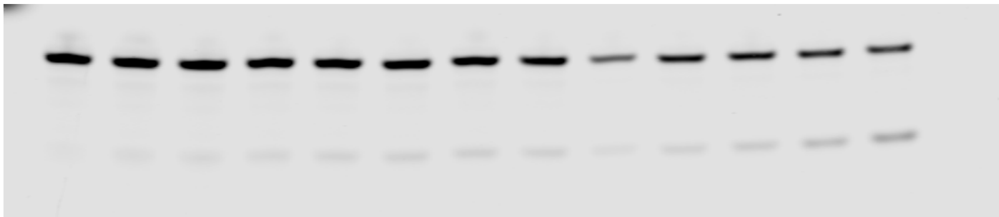

- S (16 nt)
- P (8 nt)

UGUA

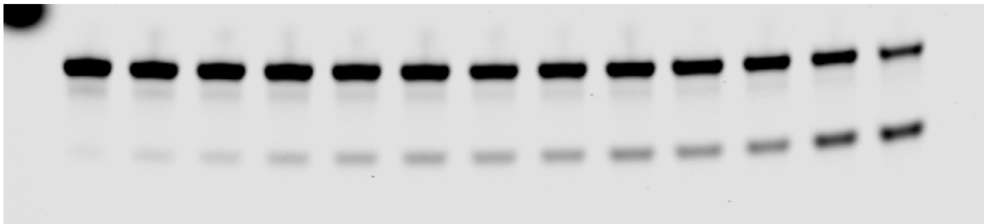

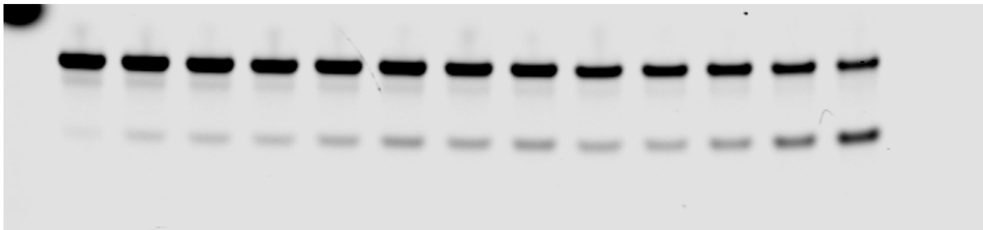

CGUU

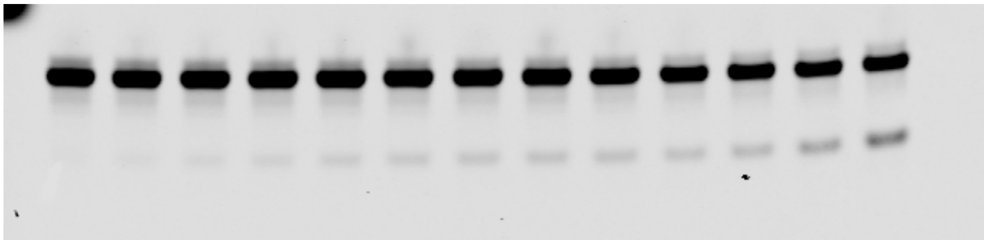

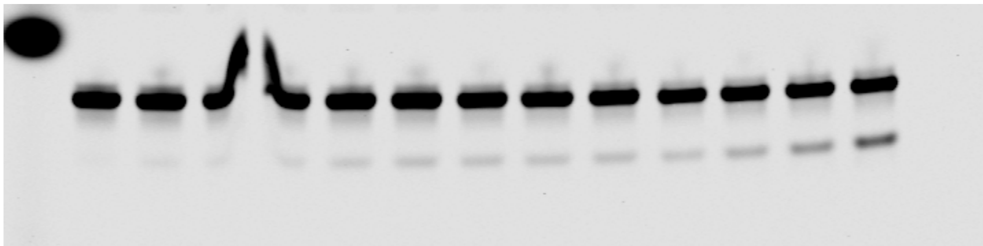

GGUU

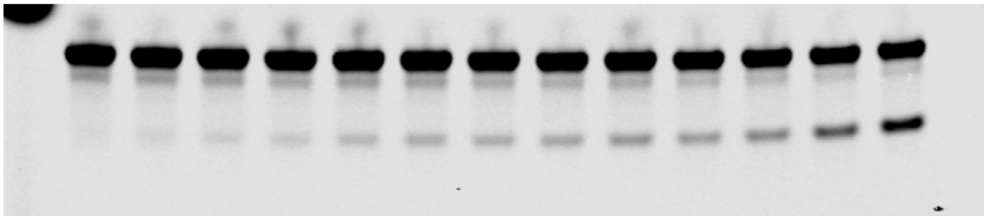

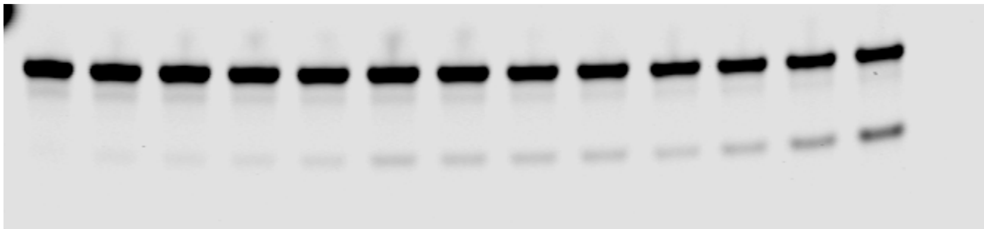

AGUU

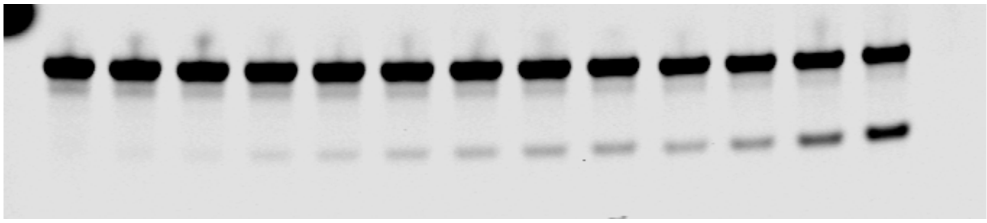

- S (16 nt)
- P (8 nt)

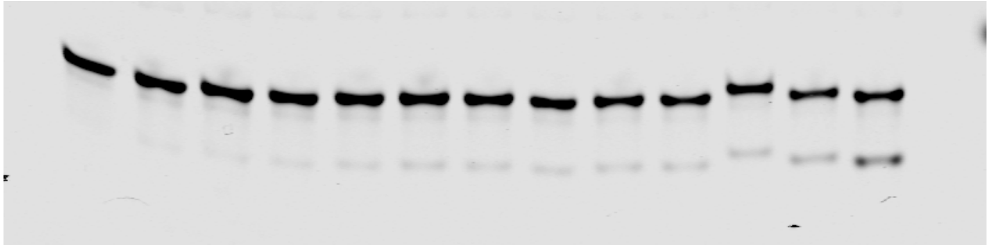

- S (16 nt)
- P (8 nt)

Normalized initial rates:

UGUU	UGUG	UGUC	UGUA	CGUU	GGUU	AGUU
1.03	0.86	0.3	0.6	0.14	0.2	0.17
0.97	0.86	0.44	0.63	0.19	0.19	0.18

Figure 2C

GATA3- UGUU

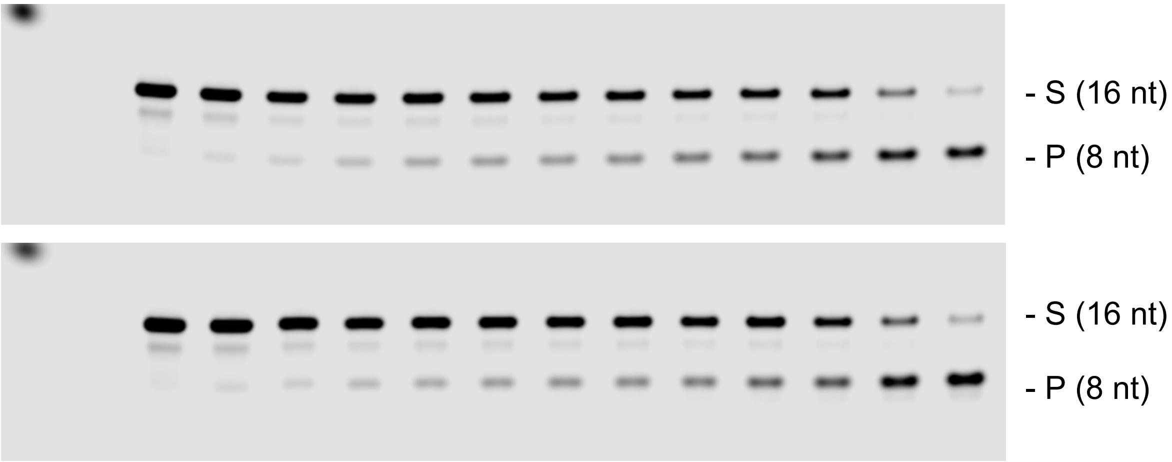

GATA3- CGUC

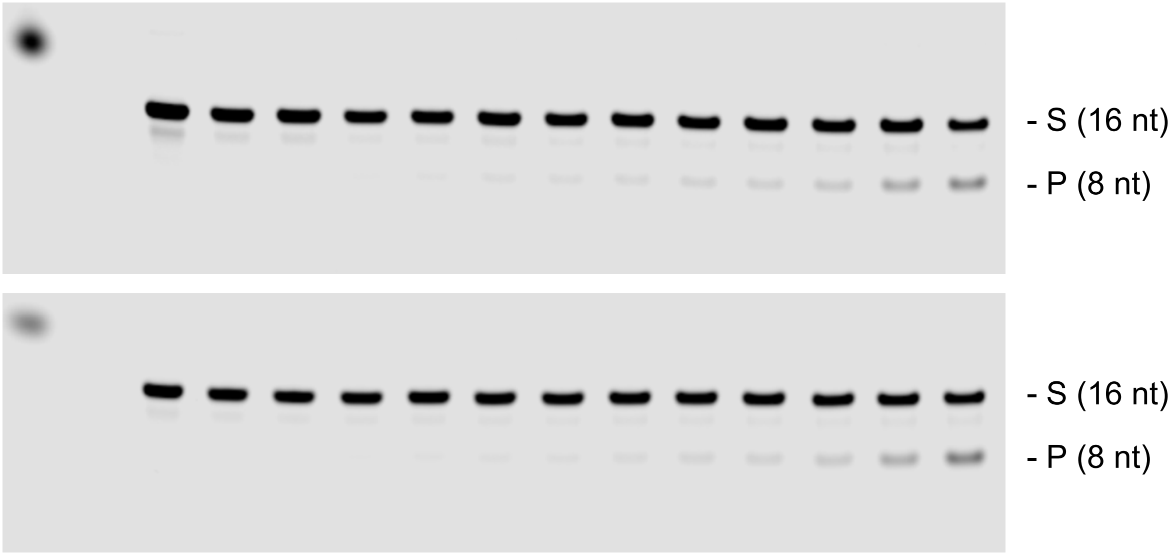

Cjun- UGUU

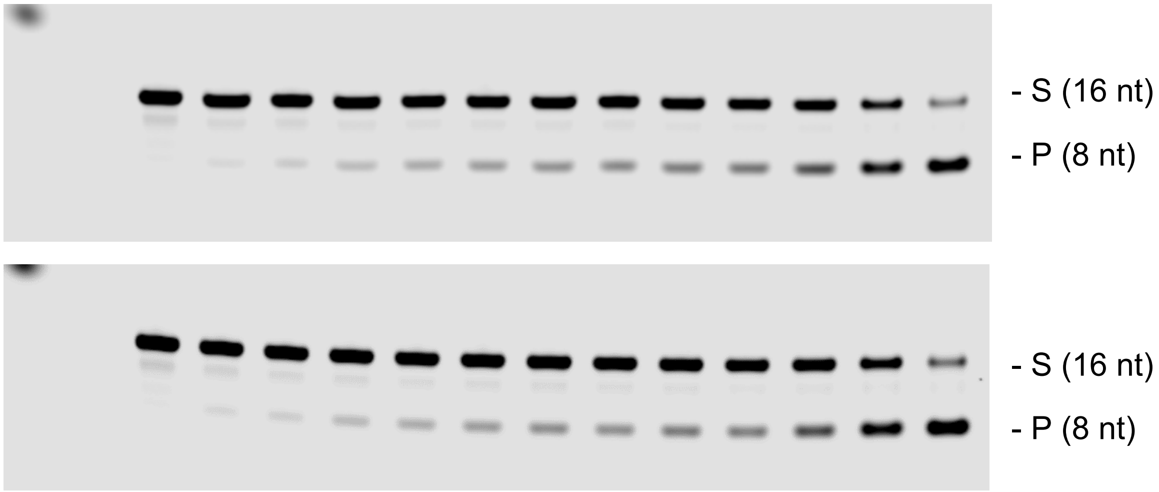

Cjun- CGUU

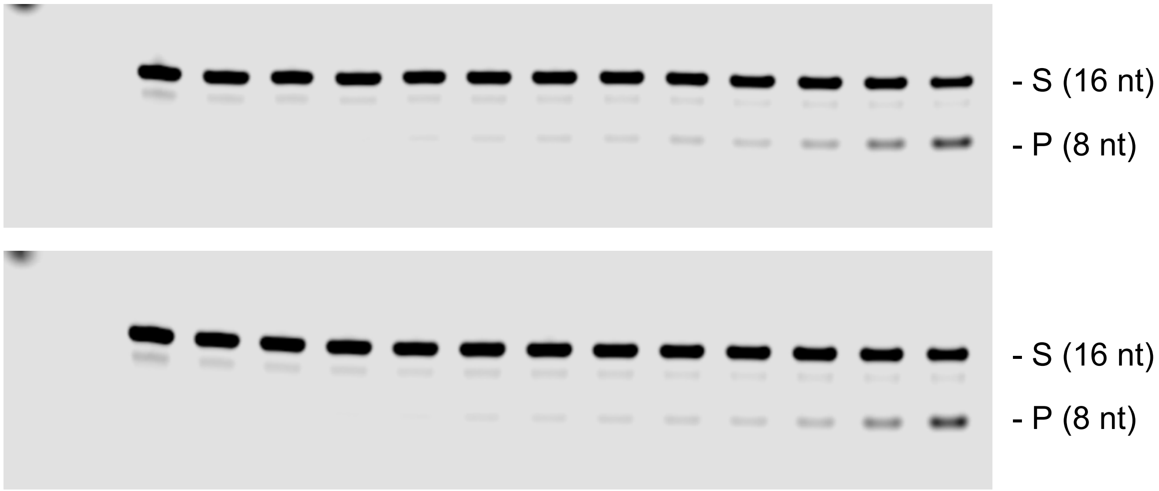

HTT- UGUU

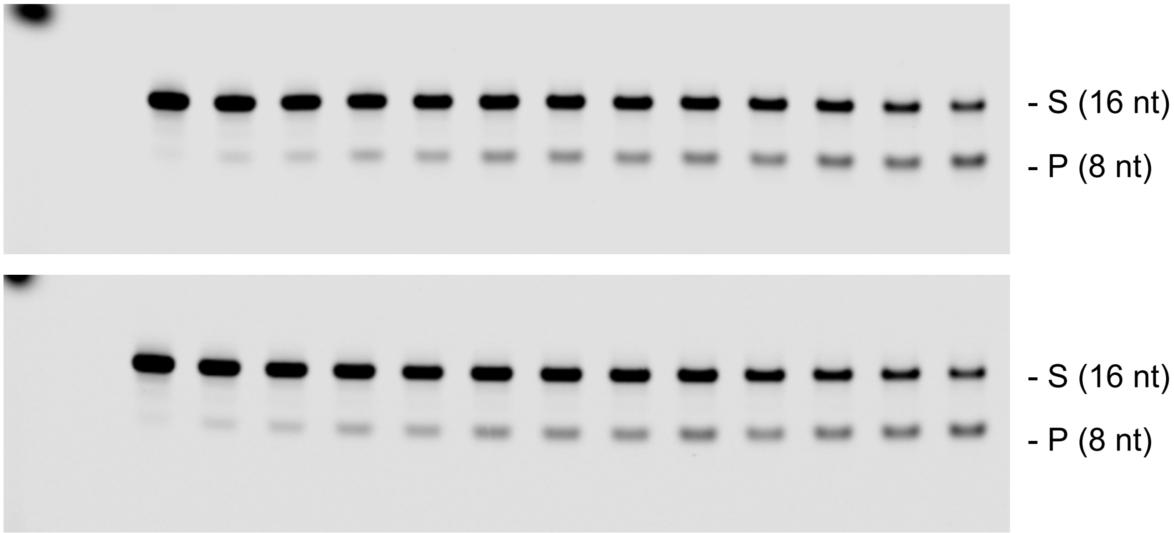

HTT- CGUU

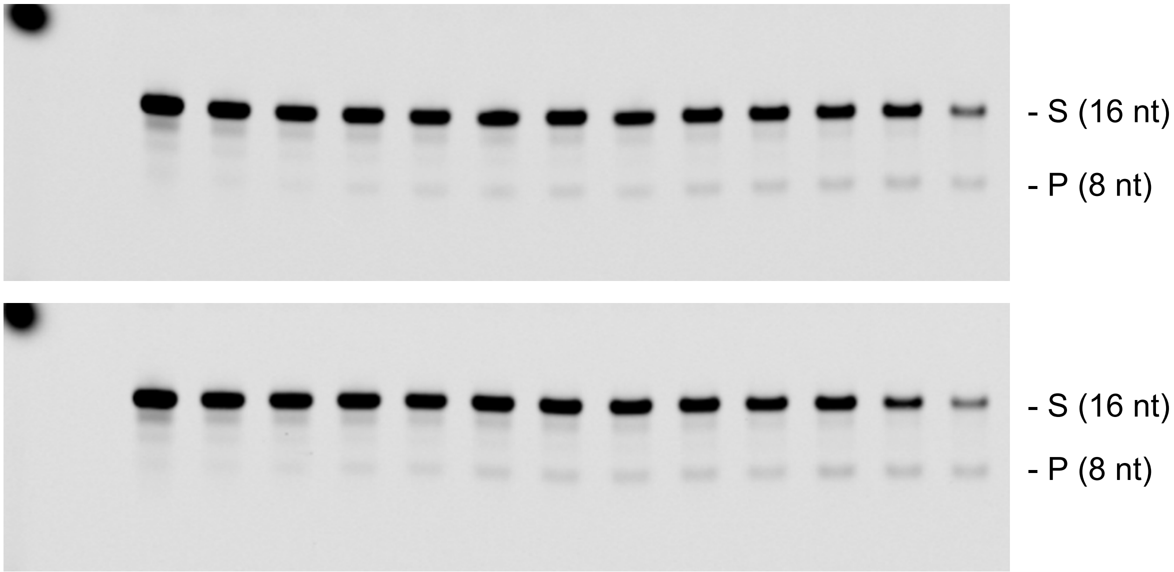

PCSK9- UGUU

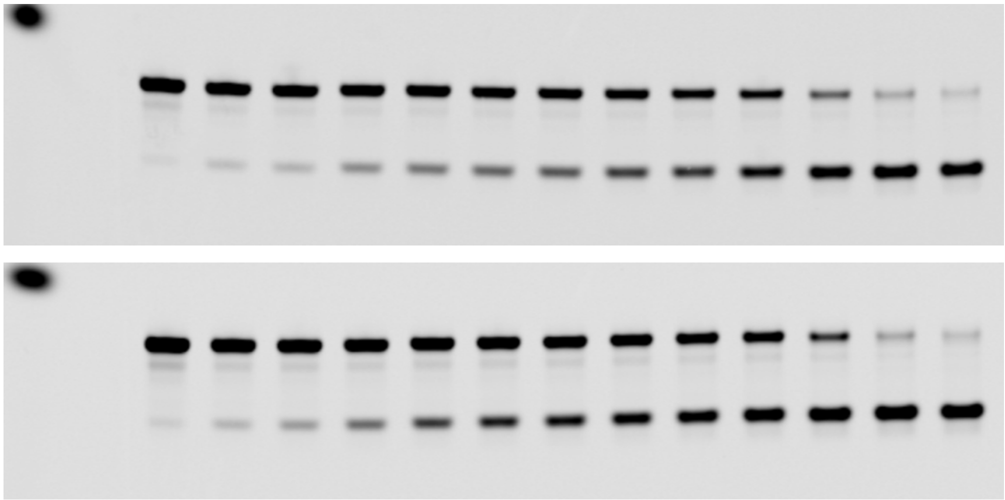

- S (16 nt)
- P (8 nt)
- S (16 nt)
- P (8 nt)

PCSK9- AGUU

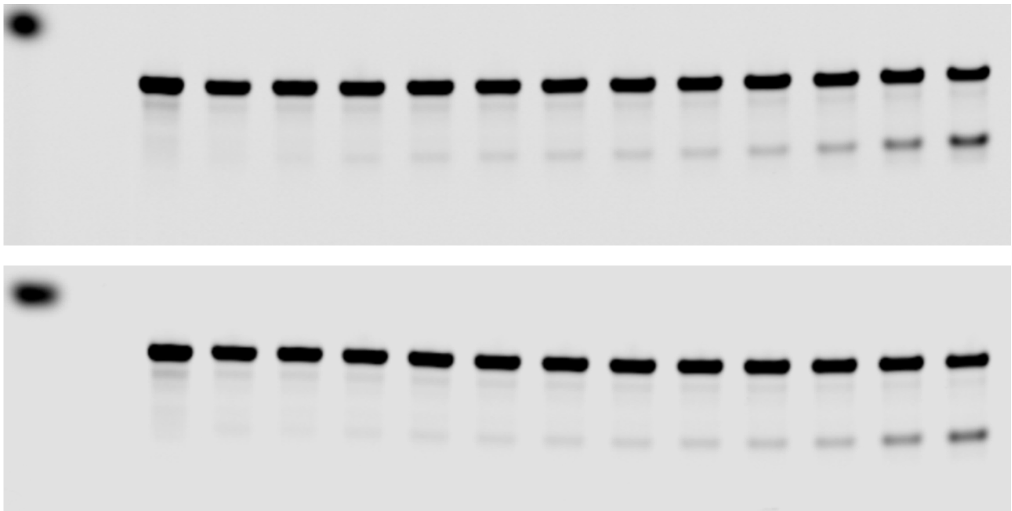

- S (16 nt)
- P (8 nt)
- S (16 nt)
- P (8 nt)

GATA3 normalized initial rates

	CGUC	UGUU
**Replicate 1**	1.00306	9.266929
**Replicate 2**	0.99694	6.623968

cjun normalized initial rates

	CGUU	UGUU
**Replicate 1**	1.061565	5.212328
**Replicate 2**	0.938435	4.60057

HTT normalized initial rates

	UGUU	CGUU
**Replicate 1**	0.94687	0.277453
**Replicate 2**	1.05313	0.297054

PCSK9 normalized initial rates

	UGUU	AGUU
**Replicate 1**	1.00217	0.079227
**Replicate 2**	0.99783	0.079572

Figure 3B.

E.coli RNAse H1 (0.5U/µL), KN128 = Dz-46 (active), KN129 = Dz-47 (inactive), (+) or (-) denotes with or without RNAse H1. 8% denaturing PAGE gel

0.5 minute:

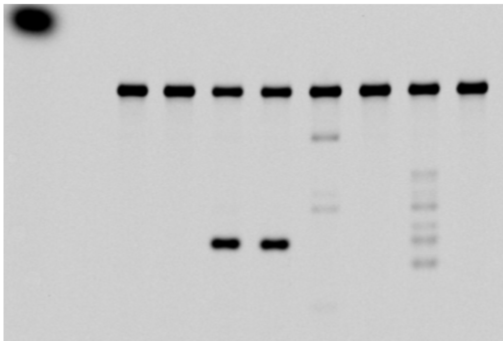

- S (60 nt)

- P (30 nt)

5 minutes:

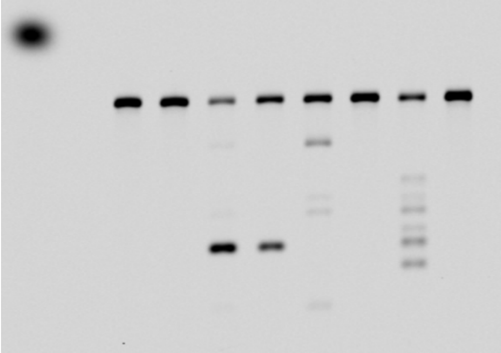

- S (60 nt)

- P (30 nt)

30 minutes:

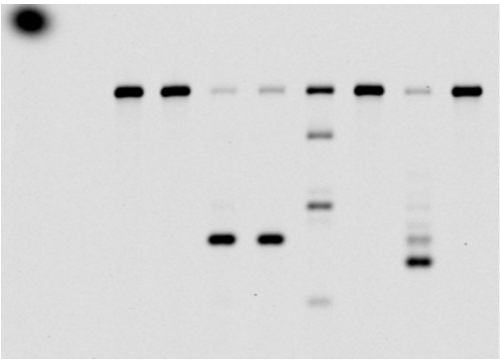

- S (60 nt)

- P (30 nt)

0.5 min

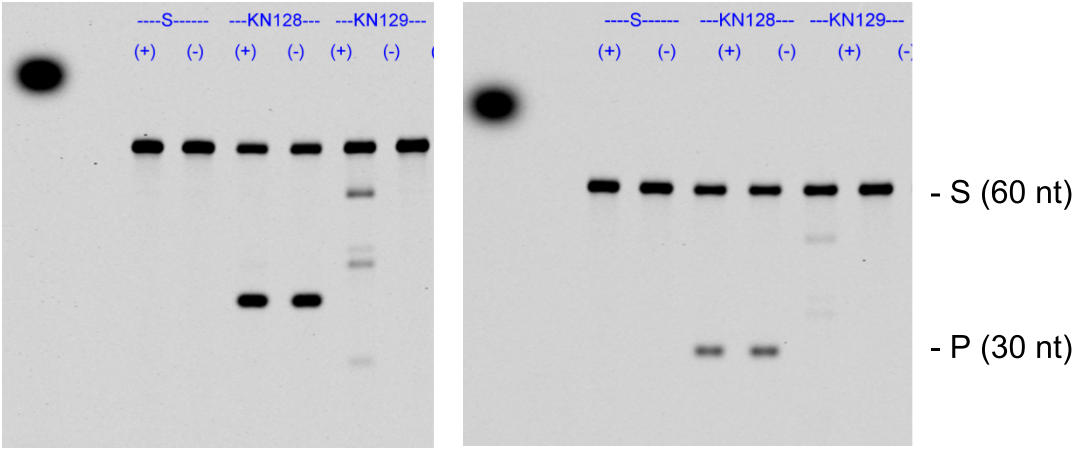

5 min

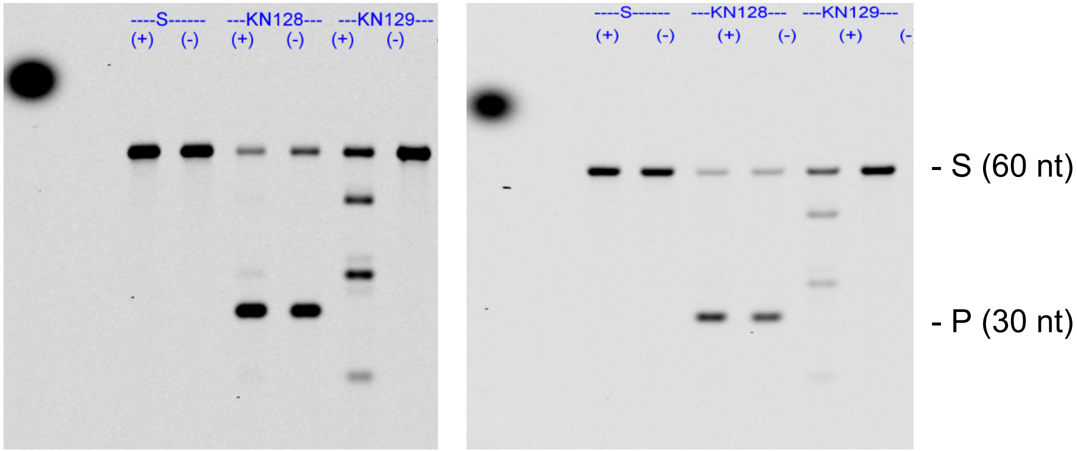

30 min

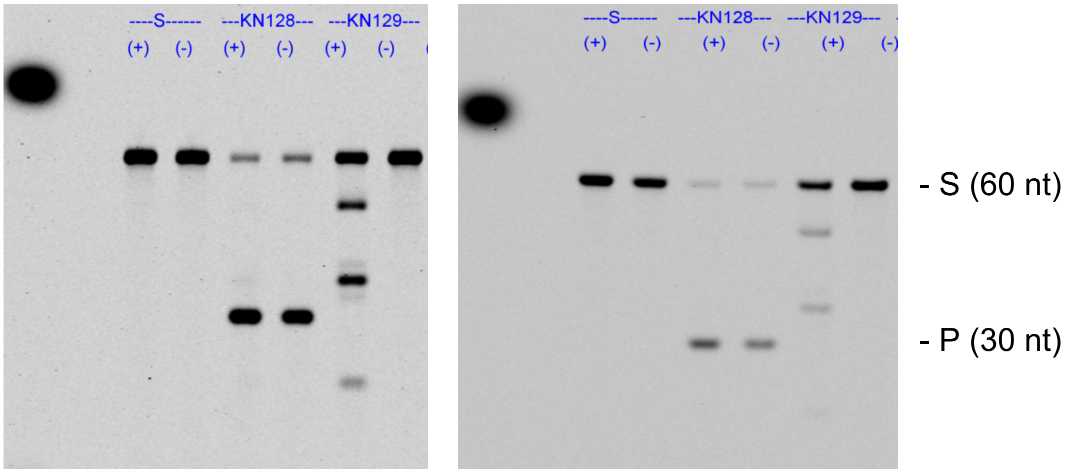

Figure 3C.

Determination of cellular RNAse H level by ELISA

Rnase H from ELISA	RNaseH (fg / cell)	Avg RNAse H (fg/cell)	Stdev
HEK293	3.08274	2.98	0.18
	3.08087		
	2.77340		
K562	1.08095	1.07	0.07
	1.13671		
	0.99730		
NCI-H441	3.96079	3.66	0.42
	3.36232		

Figure 3D. Human RNAse HI titration (0-5ng/μL), 30 min., 8% denaturing PAGE ggel

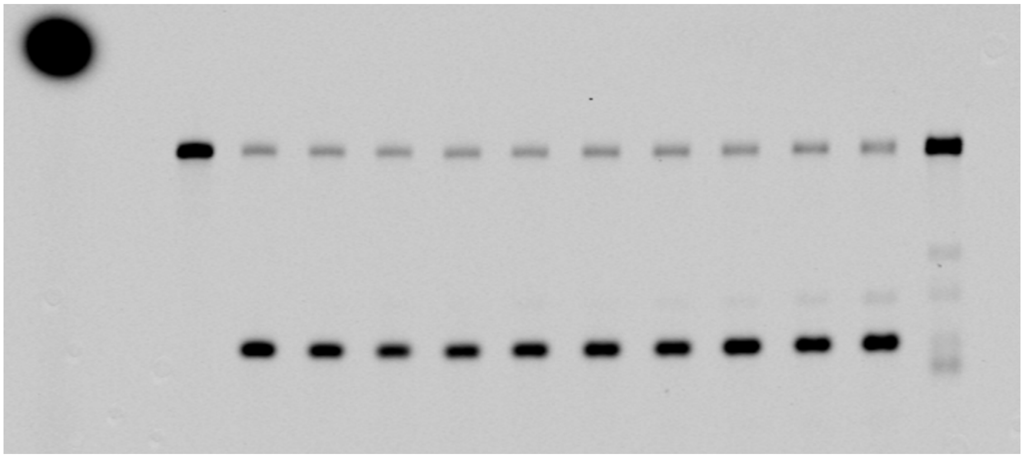

Figure 4B. Specificity analysis (KN128 = Dz-46, KN133 = Dz-49), (8% denaturing PAGE gel)

5 minutes (3 independent reactions were loaded on the same gel)

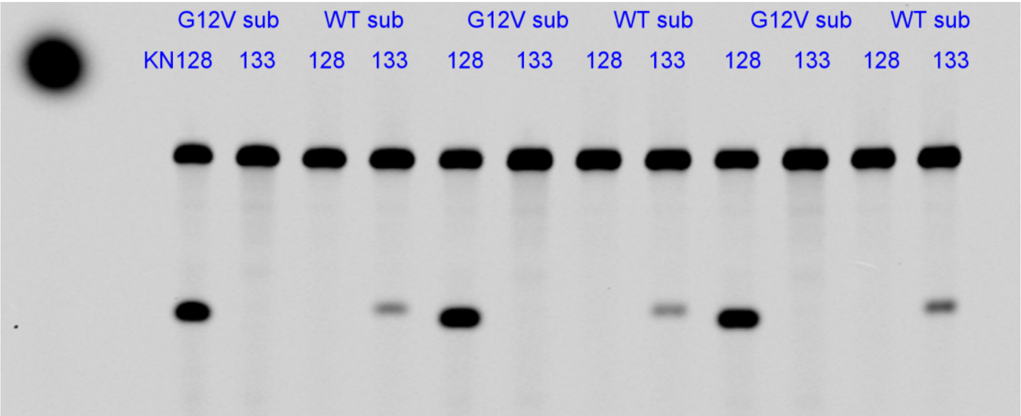

30 minutes (3 independent reactions were loaded on the same gel)

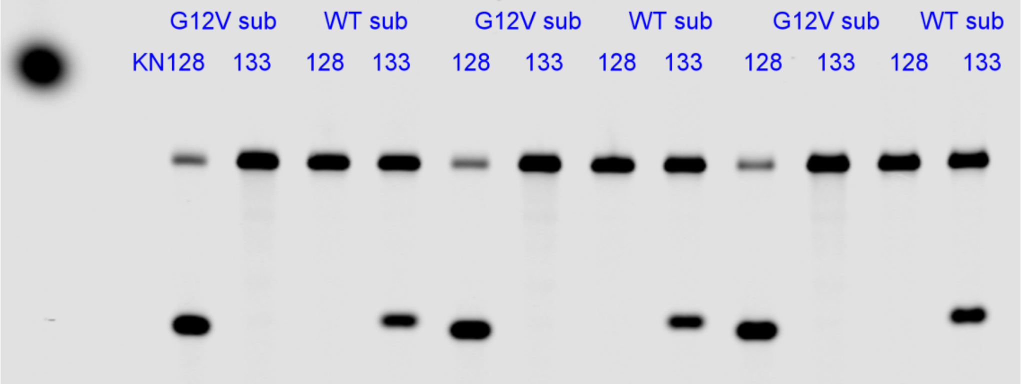

Figure 4C (20% denaturing PAGE gel)

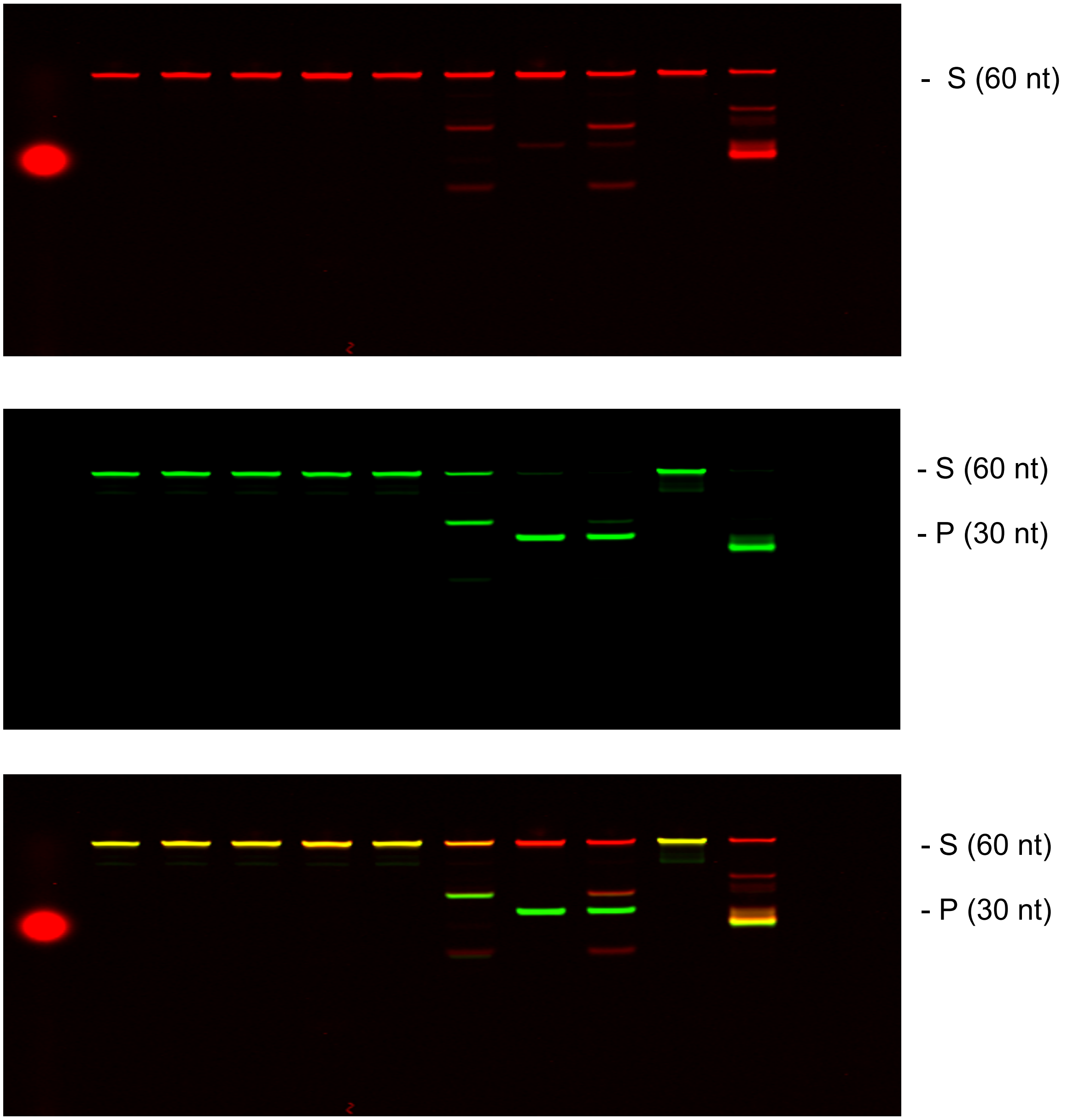

Figure 5C. Specificity In cells (RFLPA)

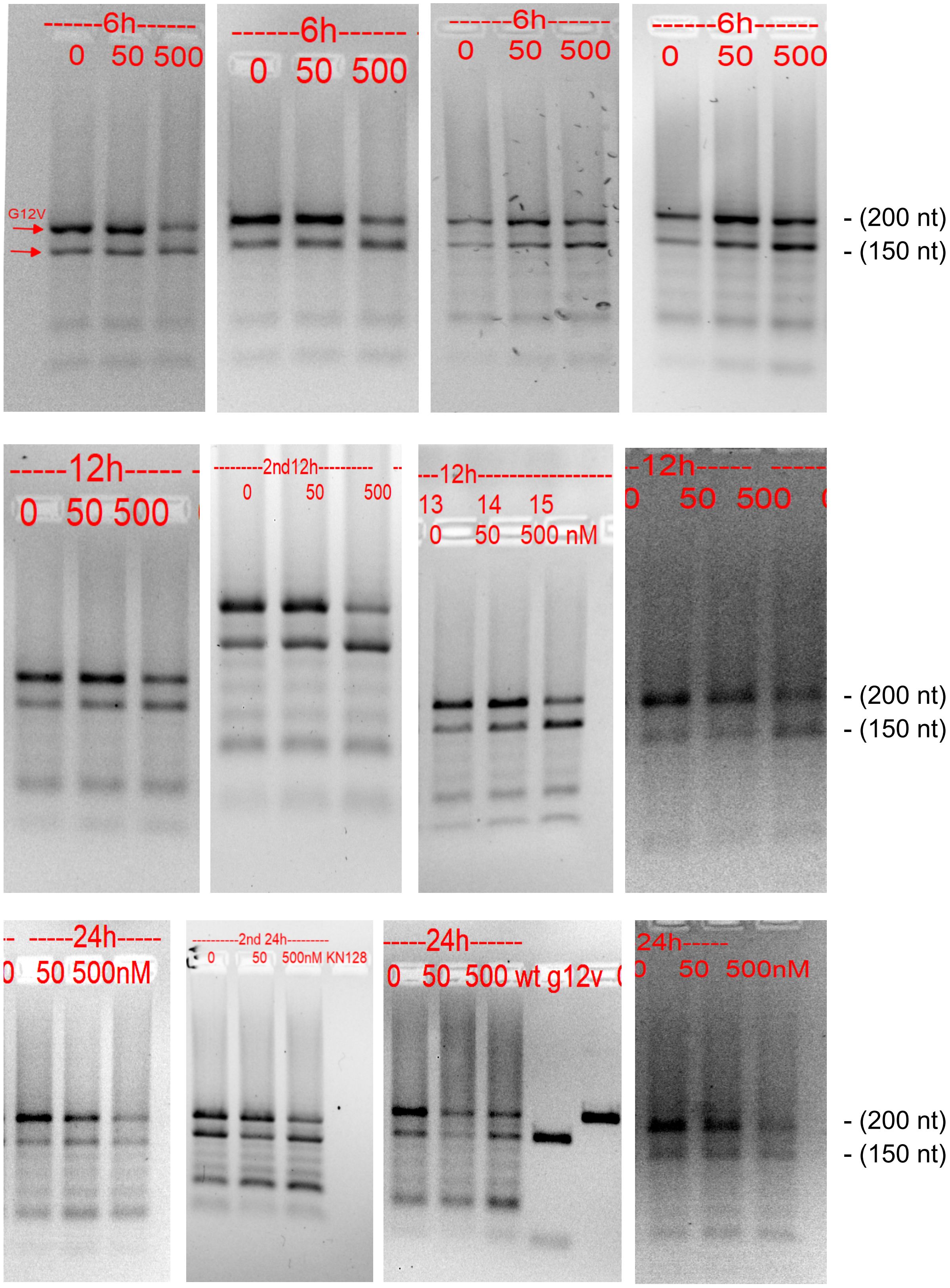

Figure 5C: plots (2 biological replicates, 2 technical replicates)

Samples	Norm. (ratio G12V/WT)	Norm. (ratio G12V/WT)	Norm. (ratio G12V/WT)	Norm. (ratio G12V/WT)	Avg	Stdev
No Dz_G12V-6h	2.38	2.35	2.14	1.63	2.13	0.35
No Dz_WT-6h	1.00	1.00	1.00	1.00		
Dz46-50nM_G12V-6h	2.12	2.18	1.87	1.79	1.99	0.19
Dz46-50nM_WT-6h	1.00	1.00	1.00	1.00		
Dz46-500nM_G12V-6h	0.79	0.84	0.94	0.87	0.86	0.06
Dz46-500nM_WT-6h	1.00	1.00	1.00	1.00		
No Dz_G12V-12h	2.50	2.66	1.59	1.45	2.05	0.62
No Dz_WT-12h	1.00	1.00	1.00	1.00		
Dz46-50nM_G12V-12h	2.18	2.37	1.50	1.26	1.83	0.53
Dz46-50nM_WT-12h	1.00	1.00	1.00	1.00		
Dz46-500nM_G12V-12h	1.06	1.03	0.73	0.69	0.88	0.20
Dz46-500nM_WT-12h	1.00	1.00	1.00	1.00		
No Dz_G12V-24h	1.94	2.10	2.89	2.38	2.33	0.42
No Dz_WT-24h	1.00	1.00	1.00	1.00		
Dz46-50nM_G12V-24h	1.52	1.27	1.40	1.48	1.42	0.11
Dz46-50nM_WT-24h	1.00	1.00	1.00	1.00		
Dz46-500nM_G12V-24h	0.87	0.91	0.90	1.24	0.98	0.17
Dz46-500nM_WT-24h	1.00	1.00	1.00	1.00		
