# Supplementary material for: Chemical evolution of an autonomous DNAzyme with allele-specific gene silencing activity
Source: Nat Commun. 2023 Apr 27;14:2413. doi: 10.1038/s41467-023-38100-9 (PMC10140269; doi:10.1038/s41467-023-38100-9)

# Source Data for Supplementary Document 2

# Chemical Evolution of an Autonomous DNAzyme with Allele-Specific Gene Silencing Activity

Kim Nguyen[1†], Turnee N. Malik[1†], and John C. Chaput[1-4*]

[1]Department of Pharmaceutical Sciences, University of California, Irvine, CA 92697-3958 USA
[2]Department of Chemistry, University of California, Irvine, CA 92697-3958 USA
[3]Department of Molecular Biology and Biochemistry, University of California, CA 92697-3958 USA
[4]Department of Chemical and Biomolecular Engineering, University of California, Irvine, CA 92697-3958 USA

## Figure S3-B: (15min)

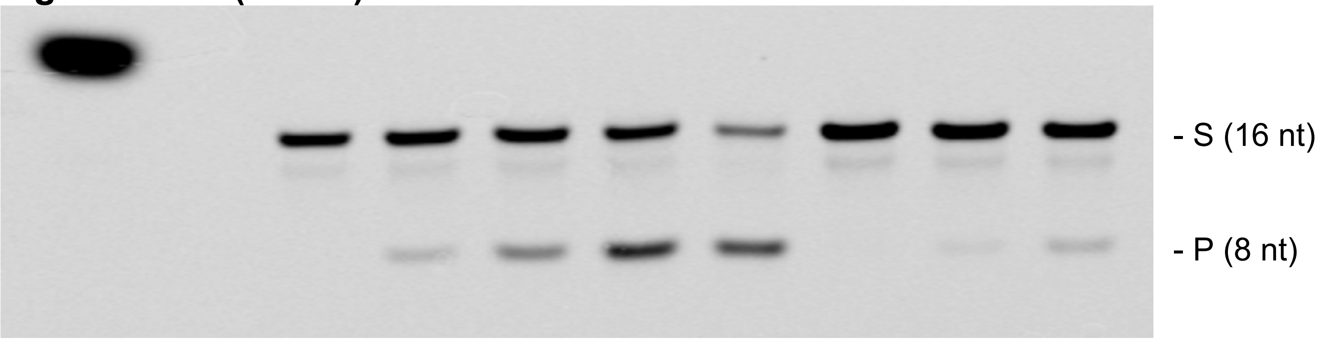


## Figure S3-C: MTO (10S:1E)

| Dz-# | 0.5 min | 5 min | 10 min | 15 min |
|---|---|---|---|---|
| 1 | 2.35 | 4.38 | 6.59 | 10.34 |
| 2 | 5.55 | 13.03 | 17.79 | 23.94 |
| 3 | 8.93 | 21.32 | 32.91 | 43.58 |
| 4 | 13.31 | 36.79 | 49.64 | 59.67 |
| 5 | 0.36 | 0.57 | 0.53 | 0.18 |
| 6 | 0.60 | 1.18 | 1.64 | 2.31 |
| 7 | 1.77 | 3.19 | 4.99 | 8.11 |

## Figure S4-B:
## 0.5 min

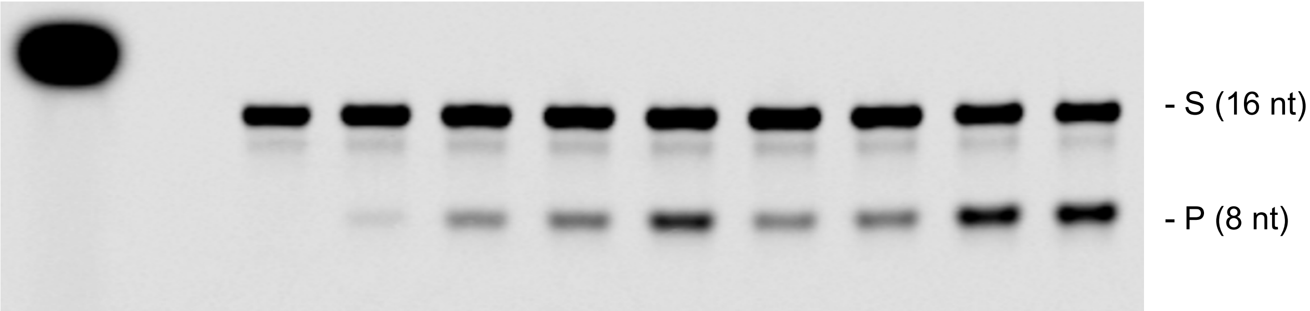


## 5 min

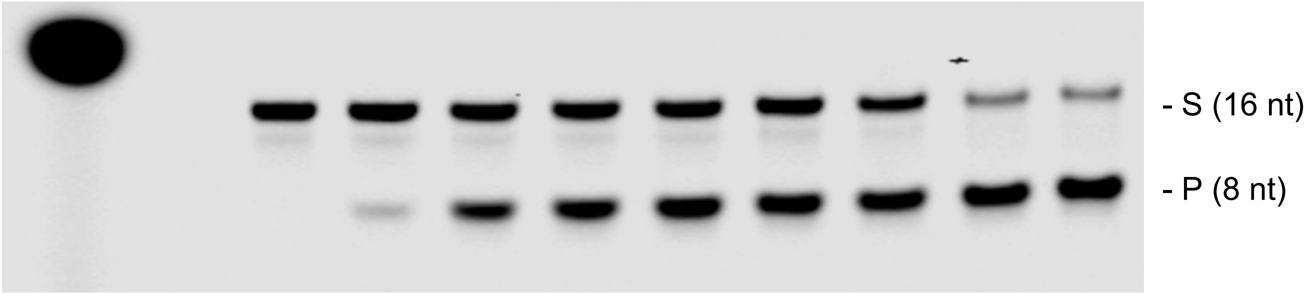

## Figure S4-C: MTO (10S:1E)

| Dz-# | 0.5 min | 5 min |
|---|---|---|
| 1 | 3.38 | 8.90 |
| 8 | 10.39 | 39.05 |
| 9 | 13.57 | 47.72 |
| 10 | 22.24 | 53.10 |
| 11 | 11.08 | 50.64 |
| 12 | 14.08 | 59.30 |
| 13 | 33.75 | 79.86 |

## Figure S5: MTO (10S:1E)

| Dz- # | Core residue | Norm. to Ome-G14 |
|---|---|---|
| 9 | DNA core | 0.76 |
| 10 | G14 | 1.00 |
| 14 | G1 | 0.06 |
| 15 | G2 | 0.95 |
| 16 | C3 | 0.01 |
| 17 | T4 | 0.13 |
| 18 | A5 | 0.01 |
| 19 | G6 | 0.14 |
| 20 | C7 | 1.08 |
| 21 | T8 | 1.19 |
| 22 | A9 | 0.38 |
| 23 | C10 | 0.01 |
| 24 | A11 | 0.95 |
| 25 | A12 | 0.31 |
| 26 | C13 | 0.17 |
| 27 | A15 | 0.82 |

**Figure S6-B:**

**10 min**

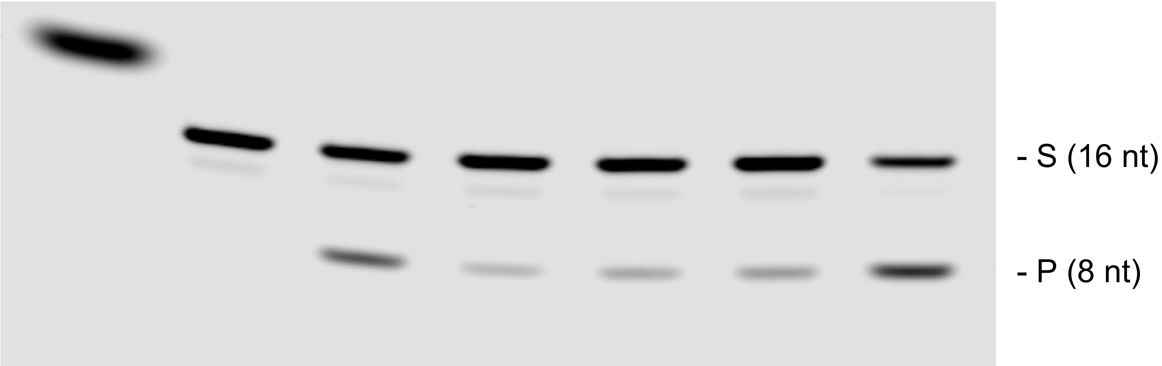


**5 min**

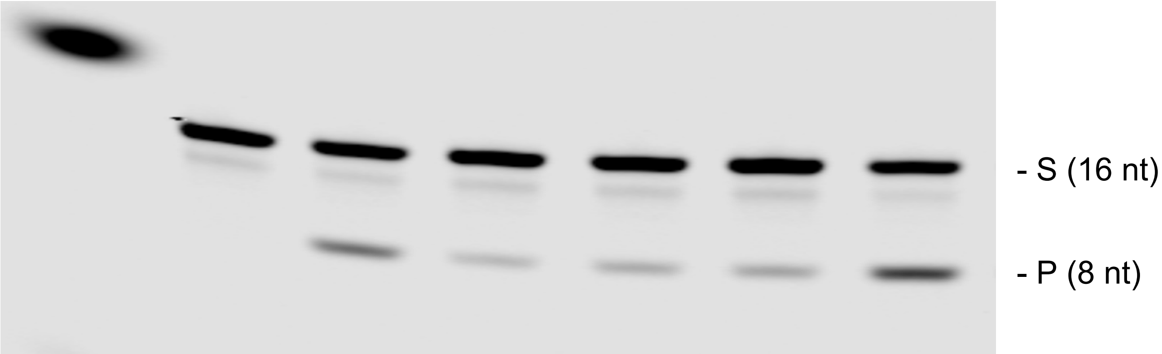


**0.5 min**

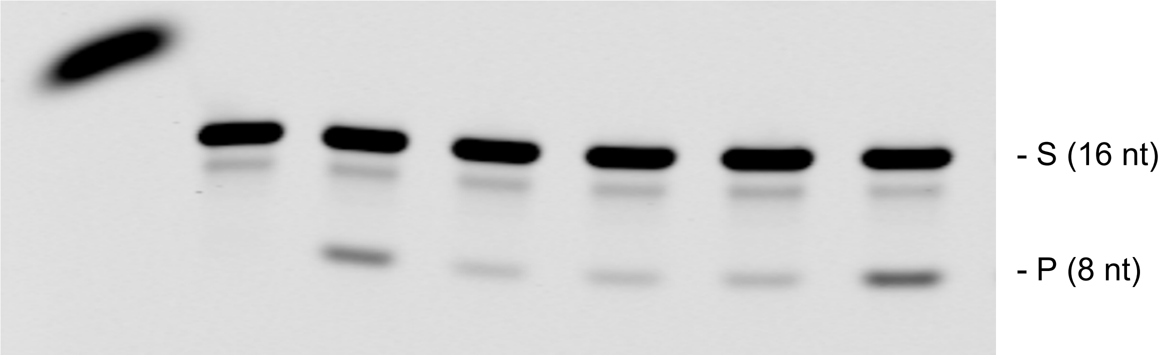

**Figure S6-C: MTO (10S:1E)**

| Dz-# | 0.5 min | 5 min | 10 min |
|---|---|---|---|
| 3 | 8.32 | 19.20 | 30.10 |
| 28 | 3.00 | 6.09 | 9.97 |
| 29 | 3.19 | 7.73 | 13.40 |
| 30 | 3.53 | 8.02 | 13.69 |
| 31 | 12.54 | 31.94 | 49.02 |

**Figure S7-B:**
**10 min**

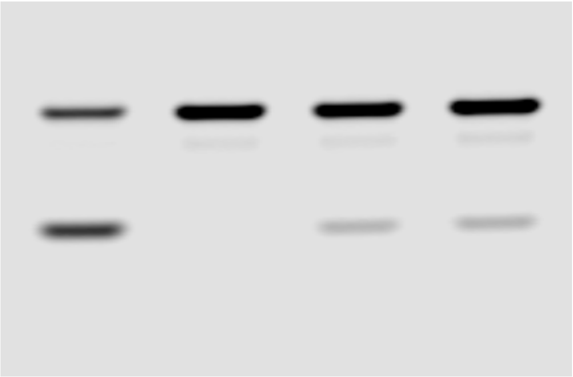


**5 min**

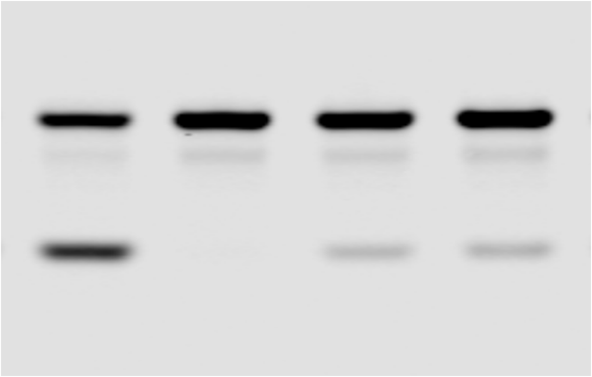


**0.5 min**

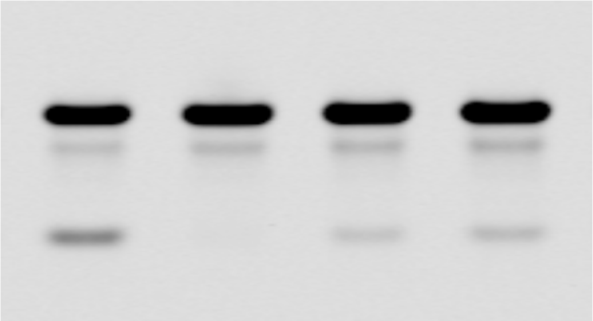

**Figure S7-C: MTO (10S:1E)**

| Dz-# | 0.5 min | 5 min | 10 min |
|---|---|---|---|
| 4 | 11.29 | 42.27 | 55.13 |
| 32 | 0.42 | 0.41 | 0.41 |
| 33 | 2.66 | 7.75 | 12.05 |
| 34 | 3.34 | 7.33 | 11.12 |

**Figure S8-B:**

**Dz-31 & Dz-35:**

**10 min** **5 min** **0.5 min**

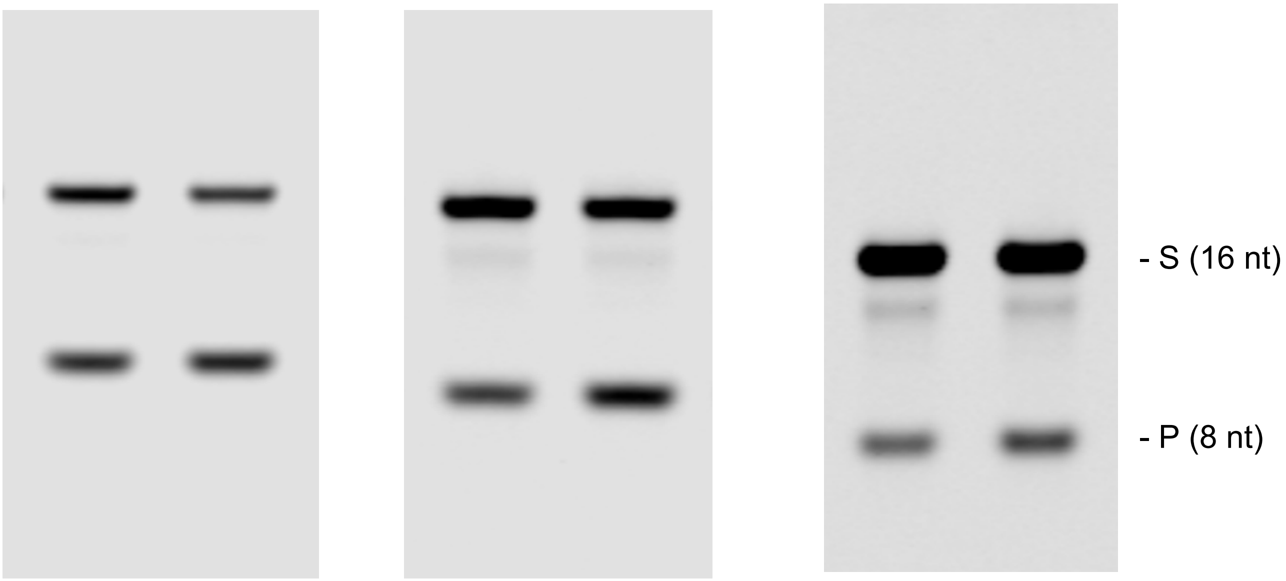


**Dz-36 & Dz-37:**

**10 min** **5 min** **0.5 min**

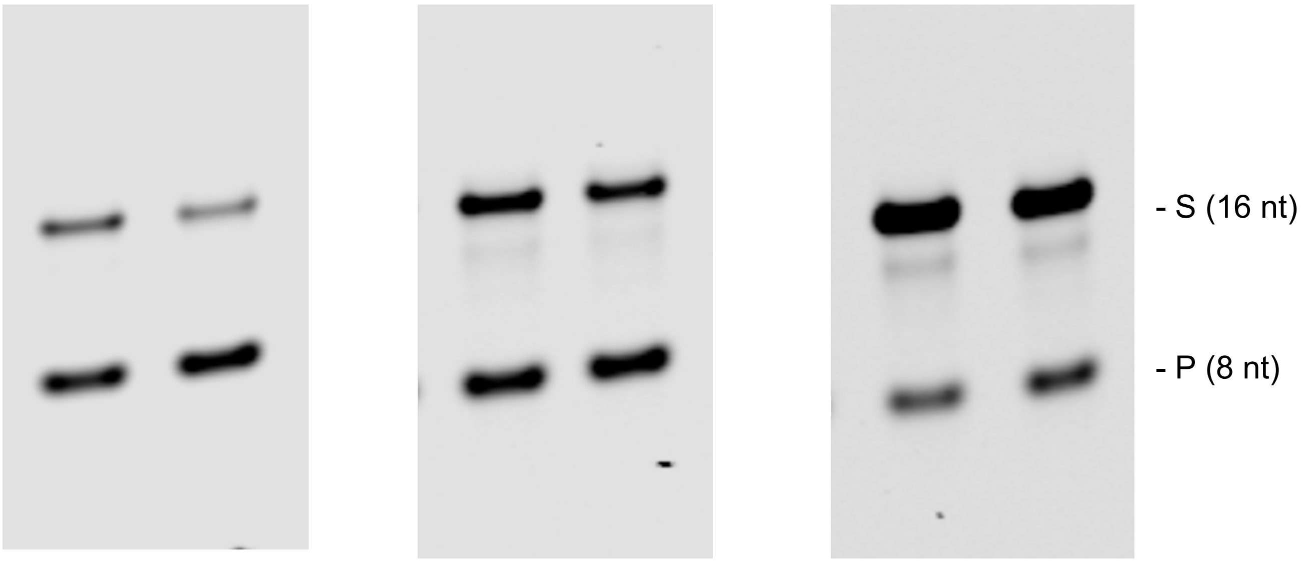

## Figure S8-C: MTO (10S:1E)

| Dz- # | 0.5 min | 5 min | 10 min |
|---|---|---|---|
| 31 | 12.54 | 31.94 | 49.02 |
| 35 | 15.92 | 43.14 | 58.03 |
| 36 | 15.70 | 47.80 | 64.42 |
| 37 | 21.24 | 58.57 | 76.54 |

## Figure S9-B:
## 5 min

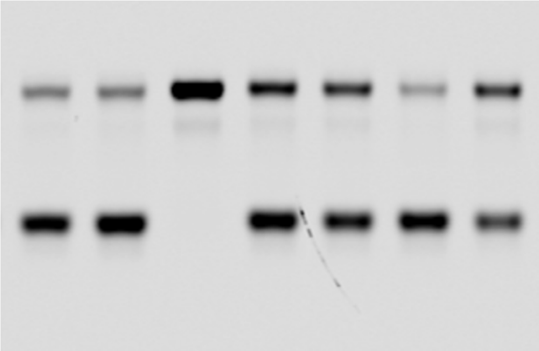

- S (16 nt)
- P (8 nt)

## 0.5 min

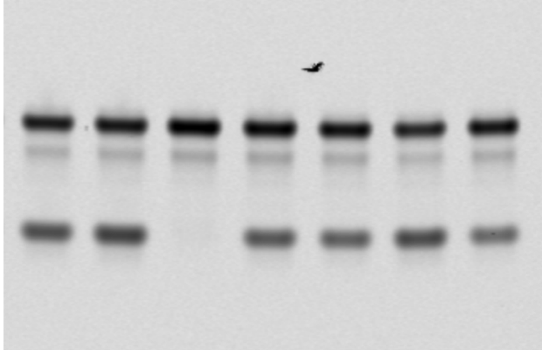

- S (16 nt)
- P (8 nt)

## Figure S9-C: MTO (10S:1E)

| Dz-# | 0.5 min | 5 min |
|---|---|---|
| 13 | 32.86 | 75.21 |
| 38 | 36.65 | 78.16 |
| 39 | 0.24 | 0.35 |
| 40 | 23.39 | 62.29 |
| 41 | 22.55 | 61.03 |
| 42 | 39.61 | 82.33 |
| 43 | 19.13 | 54.44 |

**Figure S10-B:**
**5 min**

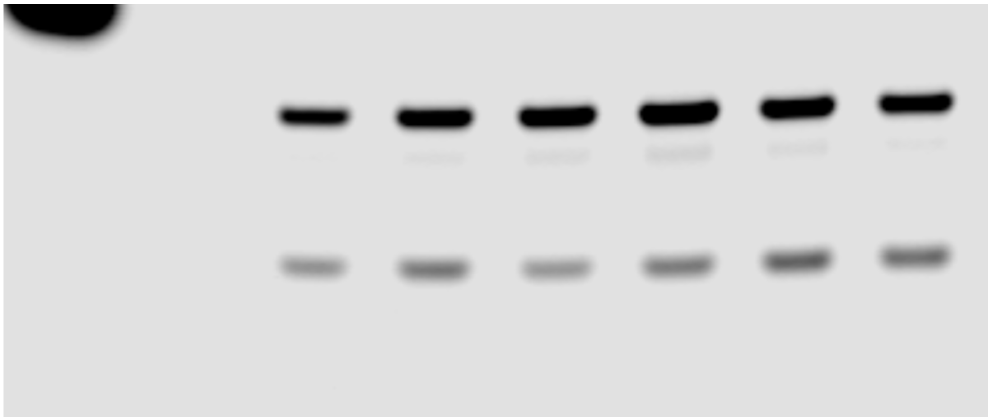


**10 min**

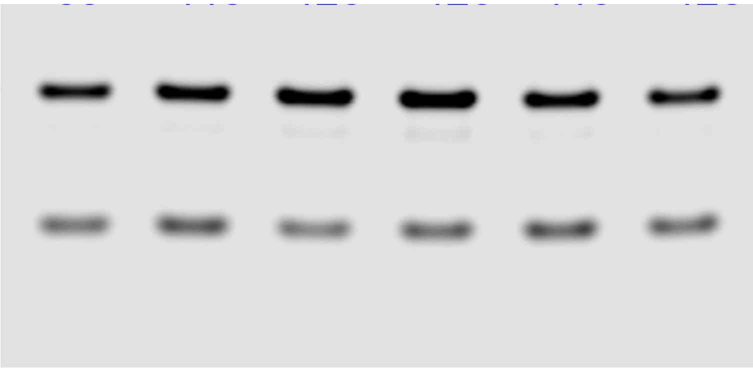


**15 min**

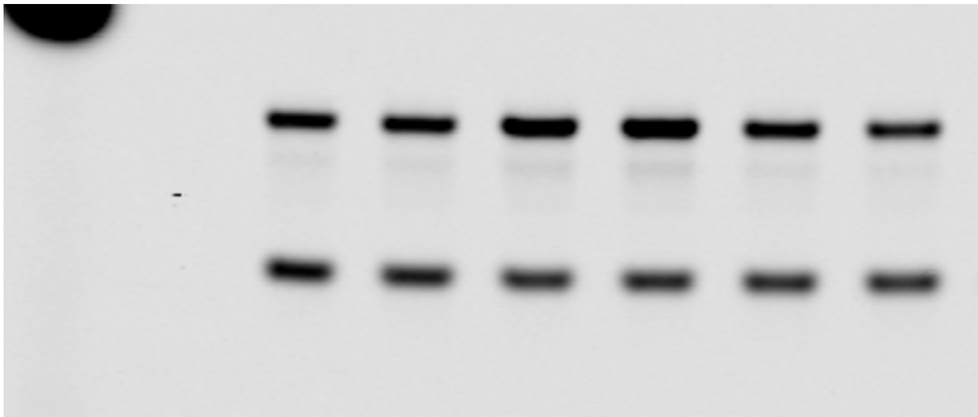

**Figure S10-C: MTO (100S:1E)**

| Dz- # | 5 min | 10 min | 15min |
|---|---|---|---|
| 38 | 28.09 | 38.38 | 49.89 |
| 44 | 26.32 | 38.49 | 47.49 |
| 43 | 17.22 | 28.05 | 36.23 |
| 45 | 17.49 | 27.60 | 33.67 |
| 42 | 26.85 | 37.72 | 46.65 |
| 46 | 29.71 | 40.90 | 51.95 |

## Figure S12-A: Un-cropped gels (0-120 min)

### Dz-1

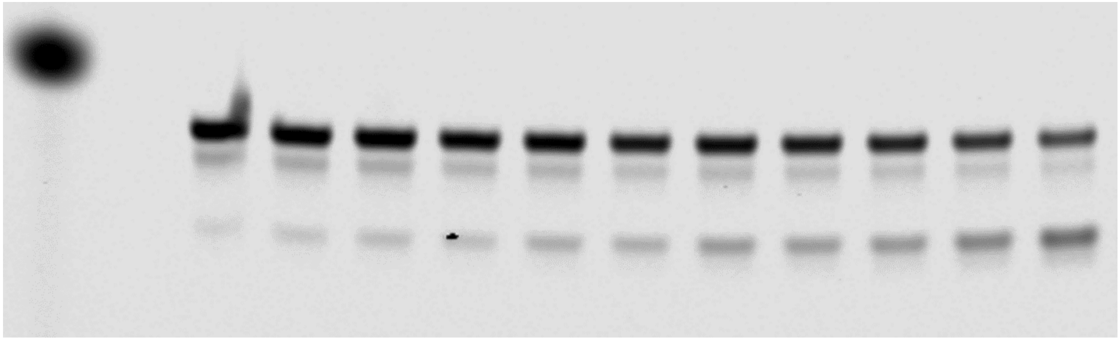

- S (16 nt)
- P (8 nt)

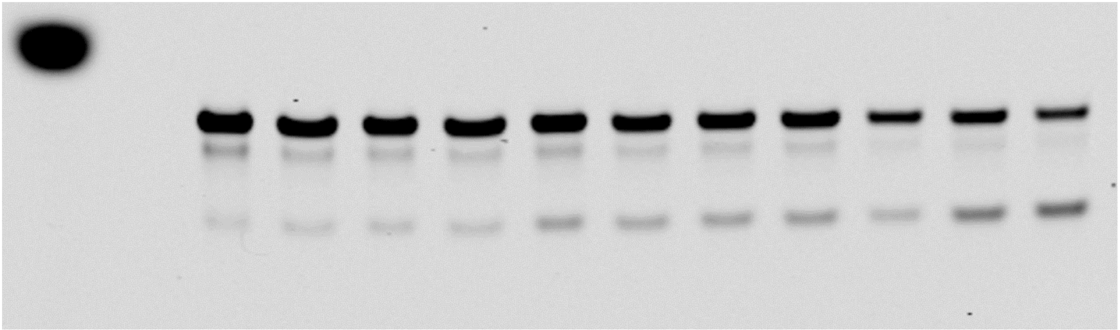

- S (16 nt)
- P (8 nt)

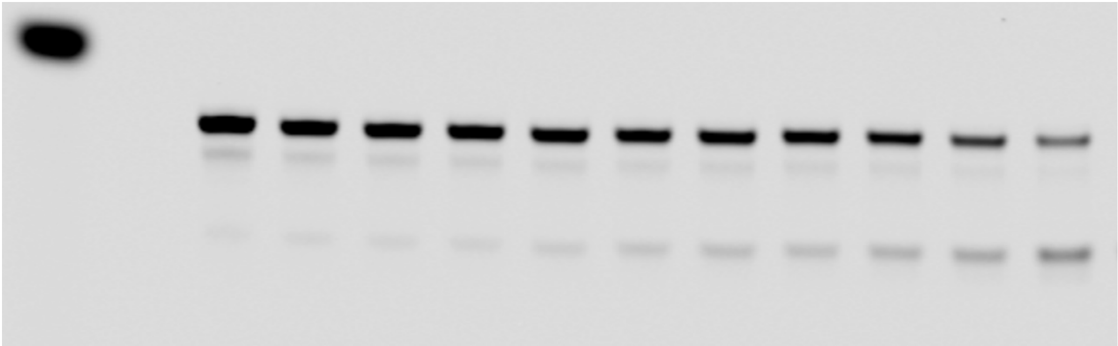

- S (16 nt)
- P (8 nt)

**Dz-3**

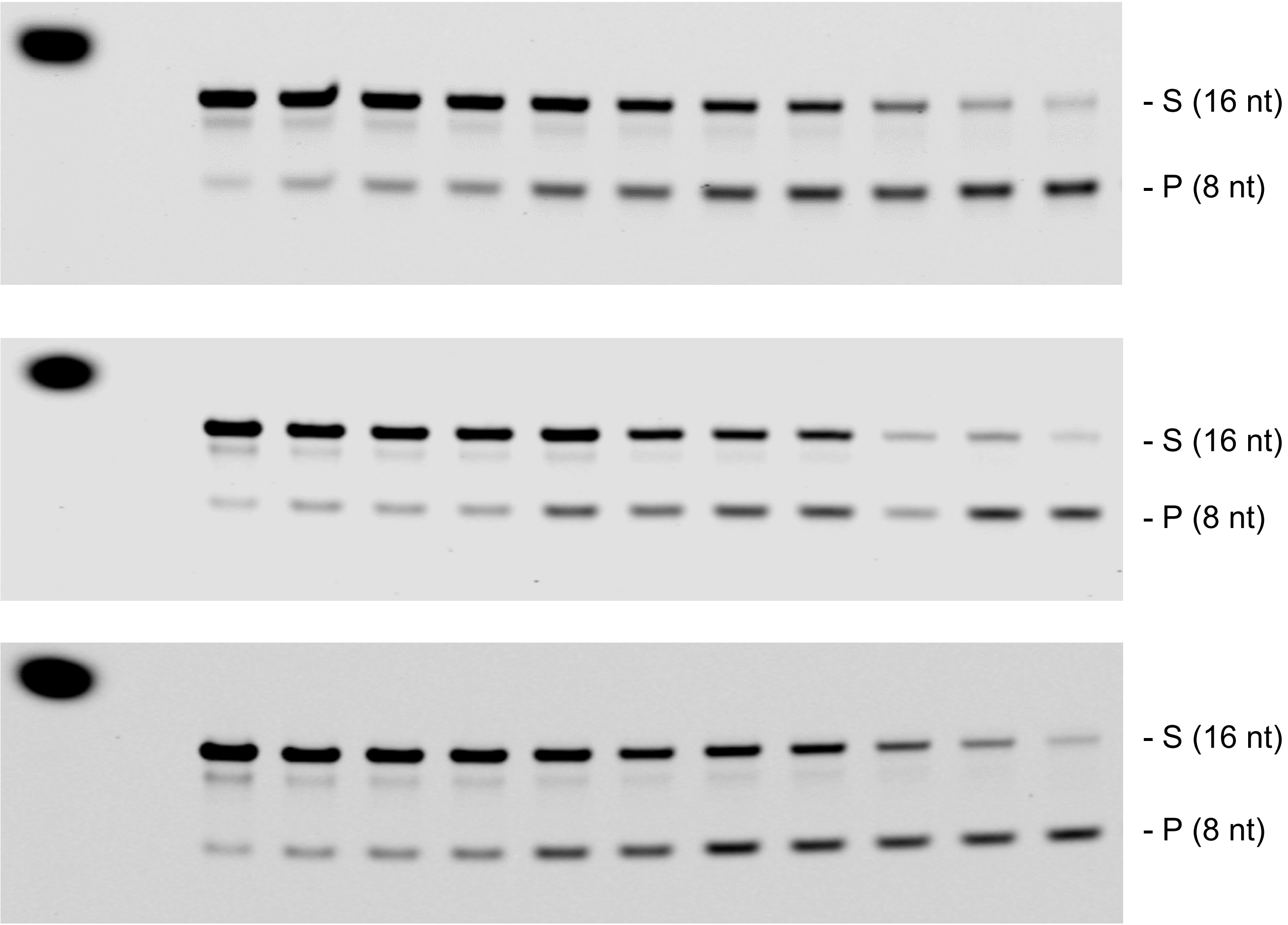


**Dz-4**

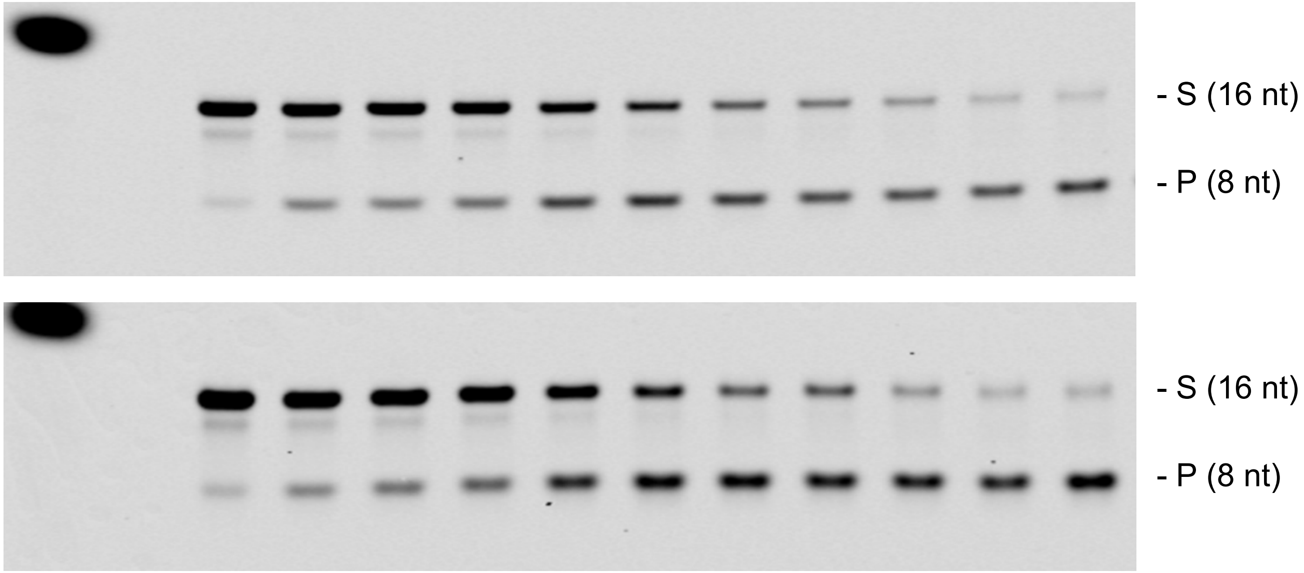

**Dz-12**

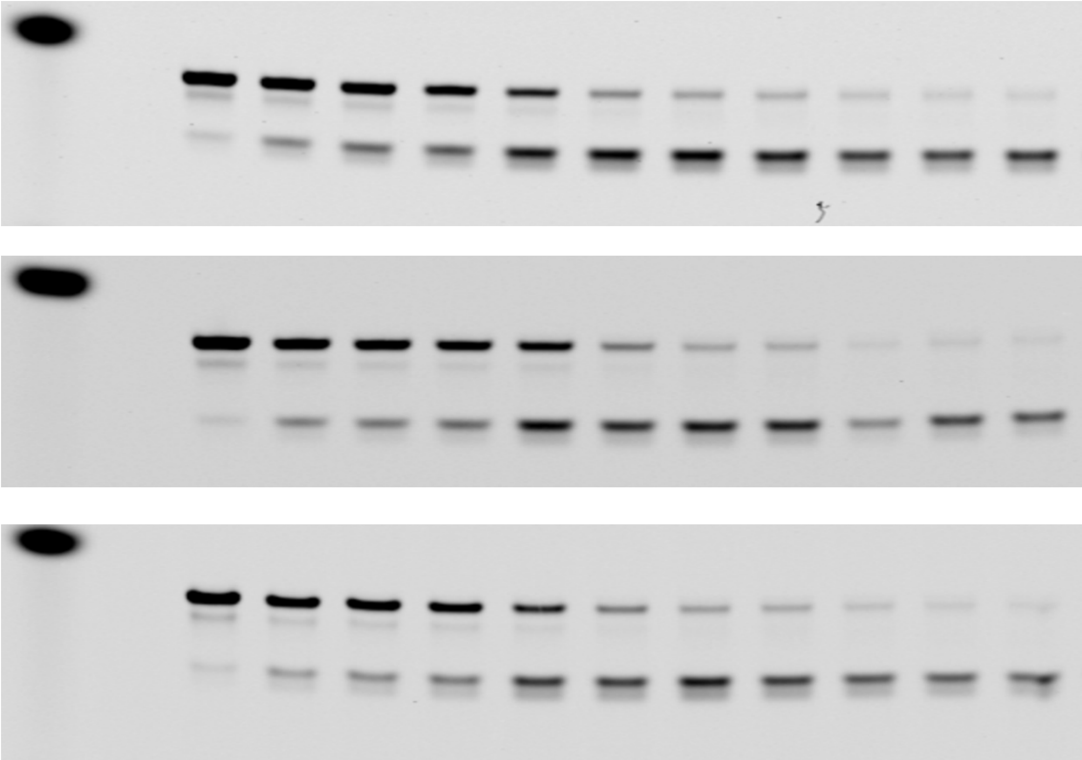

- S (16 nt)
- P (8 nt)
- S (16 nt)
- P (8 nt)
- S (16 nt)
- P (8 nt)

**Dz-13**

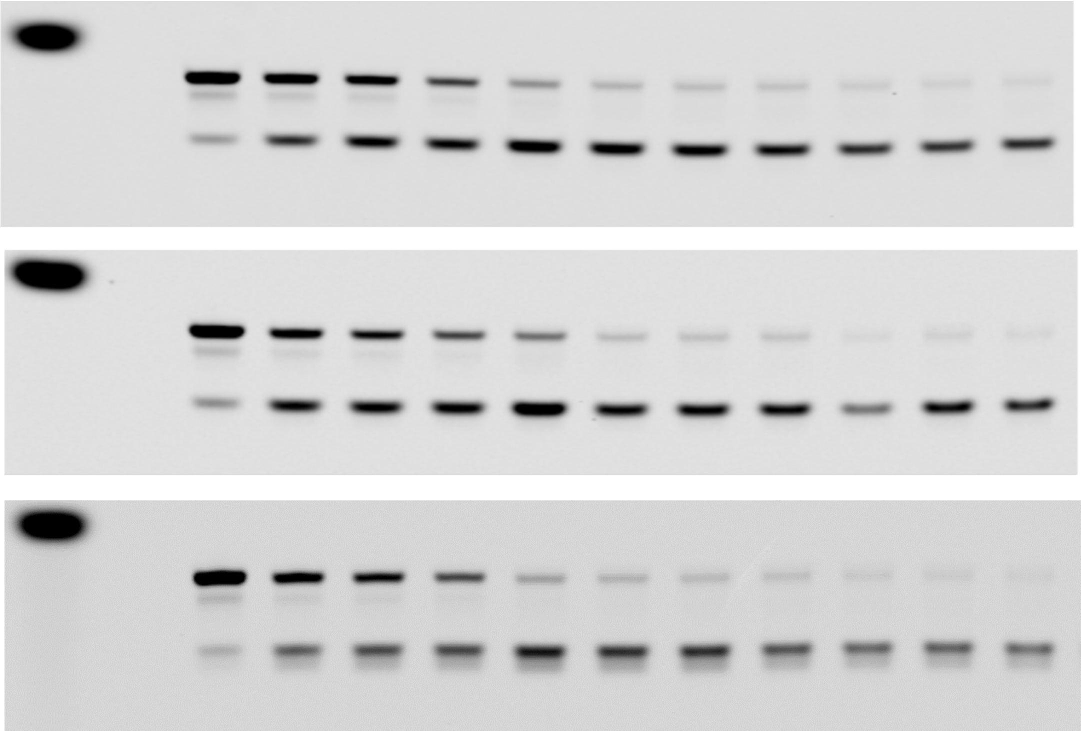

- S (16 nt)
- P (8 nt)
- S (16 nt)
- P (8 nt)
- S (16 nt)
- P (8 nt)

**Dz-31**

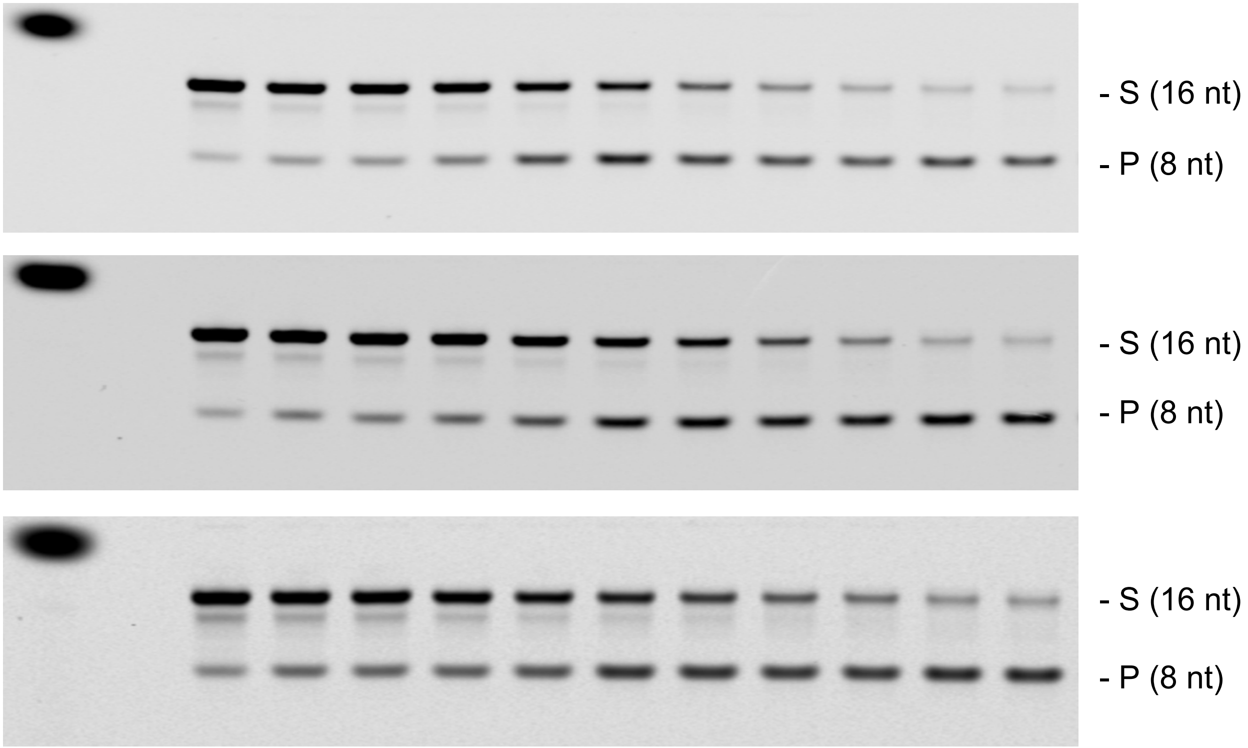
- S (16 nt)
- P (8 nt)
- S (16 nt)
- P (8 nt)
- S (16 nt)
- P (8 nt)

**Dz-38**

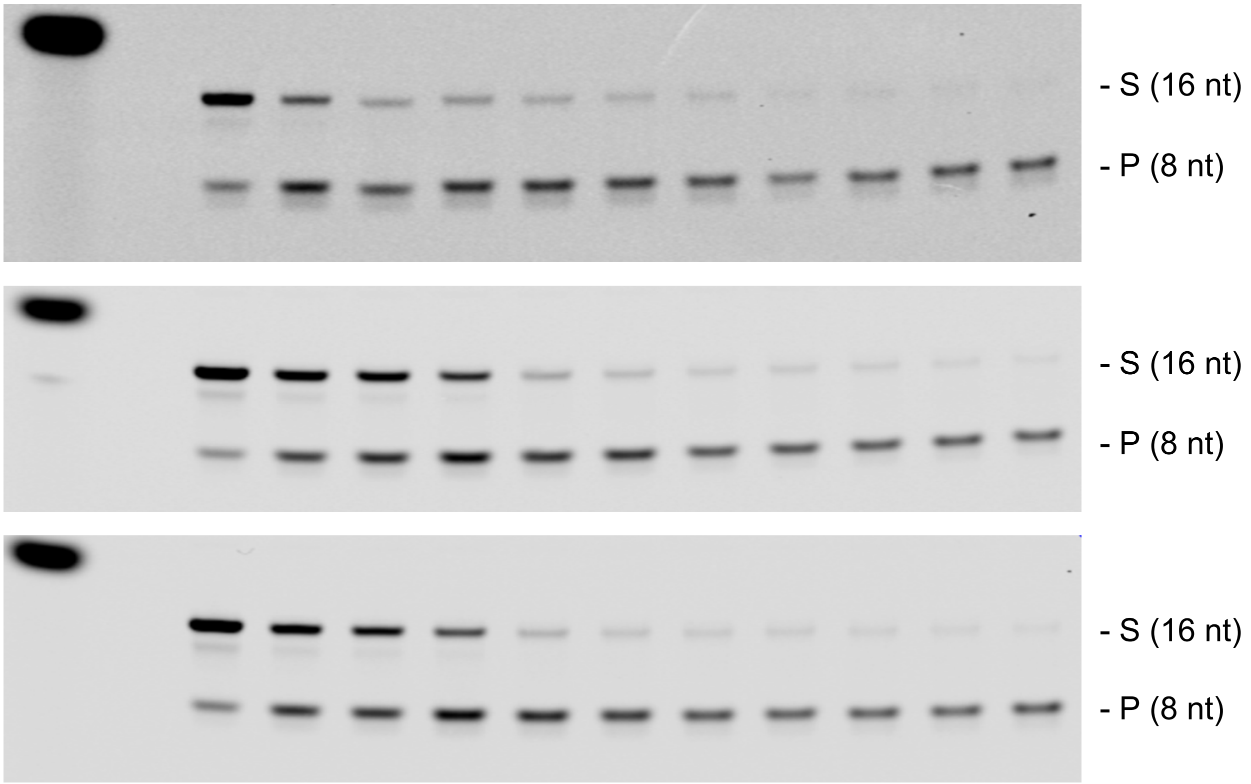
- S (16 nt)
- P (8 nt)
- S (16 nt)
- P (8 nt)
- S (16 nt)
- P (8 nt)

**Dz-42**

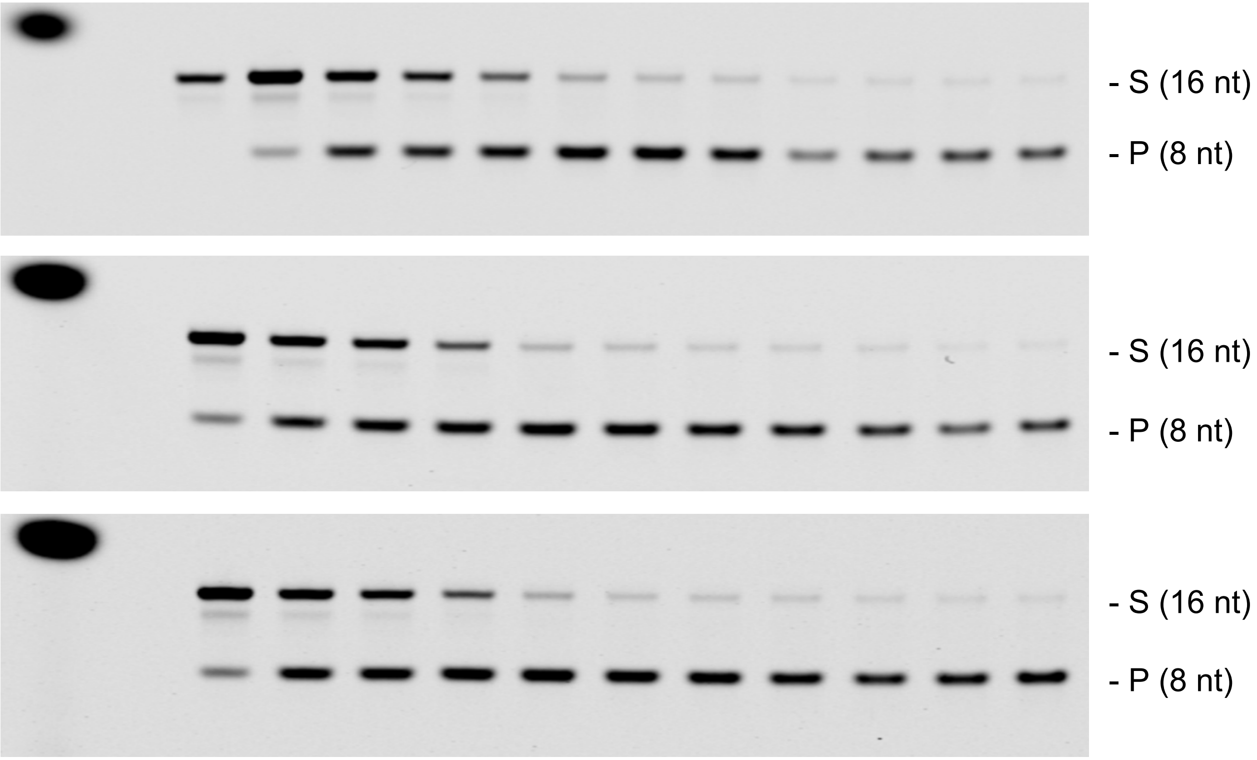
- S (16 nt)
- P (8 nt)
- S (16 nt)
- P (8 nt)
- S (16 nt)
- P (8 nt)

**Dz-46**

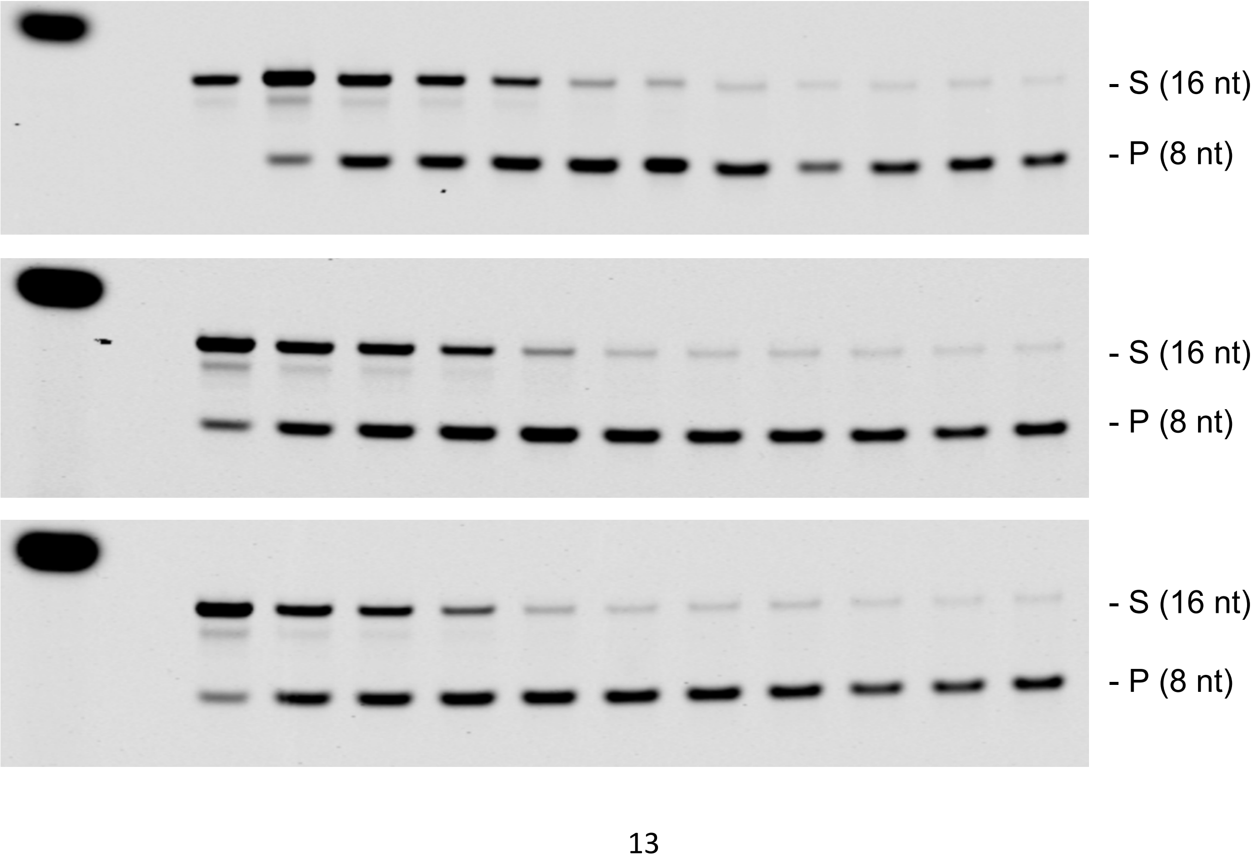
- S (16 nt)
- P (8 nt)
- S (16 nt)
- P (8 nt)
- S (16 nt)
- P (8 nt)

## Figure S12-B & -C:

| MTO (10S:1E) Time (minutes) | Dz-1, r1 | Dz-1, r2 | Dz-1, r3 |
|---|---|---|---|
| 0 | 0.74 | 1.04 | 0.69 |
| 0.5 | 2.12 | 1.99 | 1.52 |
| 1 | 2.95 | 2.61 | 2.08 |
| 2 | 2.48 | 3.27 | 2.74 |
| 5 | 5.83 | 5.08 | 4.38 |
| 10 | 7.40 | 7.33 | 5.92 |
| 15 | 10.26 | 9.10 | 8.96 |
| 18 | 11.10 | 9.58 | 9.64 |
| 30 | 13.98 | 13.54 | 13.89 |
| 60 | 22.83 | 24.38 | 22.22 |
| 120 | 41.61 | 41.69 | 48.66 |

| MTO (10S:1E) Time (minutes) | Dz-3, r1 | Dz-3, r2 | Dz-3, r3 |
|---|---|---|---|
| 0 | 2.11 | 3.25 | 2.65 |
| 0.5 | 8.22 | 8.05 | 8.05 |
| 1 | 11.23 | 9.06 | 11.18 |
| 2 | 17.23 | 12.28 | 13.23 |
| 5 | 27.00 | 21.37 | 24.74 |
| 10 | 36.22 | 32.12 | 37.84 |
| 15 | 46.16 | 41.88 | 42.83 |
| 18 | 52.56 | 45.83 | 47.45 |
| 30 | 62.04 | 58.76 | 56.31 |
| 60 | 77.64 | 75.66 | 70.54 |
| 120 | 88.48 | 88.17 | 86.61 |

| MTO (10S:1E) Time (minutes) | Dz-4, r1 | Dz-4, r2 |
|---|---|---|
| 0 | 2.68 | 3.76 |
| 0.5 | 13.41 | 10.70 |
| 1 | 15.42 | 15.51 |
| 2 | 20.19 | 20.30 |
| 5 | 37.40 | 39.78 |
| 10 | 50.95 | 57.05 |
| 15 | 59.94 | 66.99 |
| 18 | 63.75 | 63.98 |
| 30 | 71.13 | 76.79 |
| 60 | 81.42 | 83.45 |
| 120 | 88.26 | 86.58 |

| MTO (10S:1E) Time (minutes) | Dz-12, r1 | Dz-12, r2 | Dz-12, r3 |
|---|---|---|---|
| 0 | 2.45 | 1.58 | 1.45 |
| 0.5 | 12.93 | 13.43 | 11.89 |
| 1 | 22.51 | 20.11 | 17.18 |
| 2 | 30.29 | 26.81 | 24.16 |
| 5 | 56.90 | 49.89 | 51.34 |
| 10 | 76.10 | 66.19 | 67.86 |
| 15 | 83.75 | 85.09 | 81.99 |
| 18 | 83.26 | 86.29 | 82.42 |
| 30 | 88.23 | 88.24 | 87.71 |
| 60 | 89.94 | 93.34 | 91.48 |
| 120 | 91.70 | 93.90 | 94.16 |

| MTO (10S:1E) Time (minutes) | Dz-13, r1 | Dz-13, r2 | Dz-13, r3 |
|---|---|---|---|
| 0 | 7.48 | 7.09 | 4.61 |
| 0.5 | 43.60 | 30.27 | 28.79 |
| 1 | 56.46 | 48.30 | 42.68 |
| 2 | 68.76 | 63.74 | 55.36 |
| 5 | 83.28 | 84.25 | 82.16 |
| 10 | 90.10 | 89.78 | 85.96 |
| 15 | 91.57 | 91.49 | 88.36 |
| 18 | 91.07 | 91.42 | 89.50 |
| 30 | 94.36 | 92.88 | 90.91 |
| 60 | 96.28 | 93.88 | 91.40 |
| 120 | 96.37 | 94.39 | 94.26 |

| MTO (10S:1E) Time (minutes) | Dz-31, r1 | Dz-31, r2 | Dz-31, r3 |
|---|---|---|---|
| 0 | 4.68 | 4.86 | 5.52 |
| 0.5 | 12.04 | 10.16 | 11.71 |
| 1 | 14.36 | 12.23 | 13.67 |
| 2 | 20.38 | 16.54 | 18.61 |
| 5 | 38.62 | 27.99 | 29.95 |
| 10 | 52.65 | 43.57 | 47.78 |
| 15 | 59.59 | 53.56 | 55.54 |
| 18 | 67.36 | 61.54 | 64.39 |
| 30 | 73.96 | 72.44 | 72.00 |
| 60 | 84.73 | 84.76 | 84.37 |
| 120 | 87.98 | 88.24 | 86.69 |

| MTO (10S:1E) Time (minutes) | Dz-38, r1 | Dz-38, r2 | Dz-38, r3 |
|---|---|---|---|
| 0 | 17.29 | 10.76 | 13.18 |
| 0.5 | 64.61 | 35.17 | 40.02 |
| 1 | 73.57 | 43.81 | 48.59 |
| 2 | 78.13 | 59.57 | 65.04 |
| 5 | 86.00 | 79.50 | 84.14 |
| 10 | 88.80 | 85.86 | 88.84 |
| 15 | 91.29 | 87.41 | 90.45 |
| 18 | 95.11 | 87.30 | 91.41 |
| 30 | 95.35 | 90.50 | 92.91 |
| 60 | 95.78 | 93.11 | 93.14 |
| 120 | 97.52 | 94.96 | 95.47 |

| MTO (10S:1E) Time (minutes) | Dz-42, r1 | Dz-42, r2 | Dz-42, r3 |
|---|---|---|---|
| 0 | 8.17 | 11.98 | 11.50 |
| 0.5 | 40.98 | 38.78 | 44.57 |
| 1 | 54.85 | 51.39 | 56.94 |
| 2 | 67.60 | 69.54 | 73.09 |
| 5 | 85.52 | 89.23 | 89.40 |
| 10 | 91.12 | 90.69 | 92.75 |
| 15 | 90.76 | 92.99 | 93.42 |
| 18 | 89.77 | 94.04 | 93.77 |
| 30 | 92.52 | 93.73 | 93.93 |
| 60 | 94.12 | 94.90 | 94.84 |
| 120 | 94.96 | 95.71 | 95.94 |

| MTO (10S:1E) Time (minutes) | Dz-46, r1 | Dz-46, r2 | Dz-46, r3 |
|---|---|---|---|
| 0 | 9.58 | 10.13 | 10.70 |
| 0.5 | 40.88 | 38.55 | 44.40 |
| 1 | 51.96 | 50.80 | 57.00 |
| 2 | 67.47 | 68.91 | 73.36 |
| 5 | 86.75 | 89.03 | 88.70 |
| 10 | 92.14 | 92.82 | 92.45 |
| 15 | 92.73 | 93.55 | 92.09 |
| 18 | 91.39 | 93.34 | 91.57 |
| 30 | 93.55 | 94.81 | 91.69 |
| 60 | 94.29 | 95.02 | 94.26 |
| 120 | 95.87 | 95.60 | 94.52 |

## Figure S13:

## Dz 1 targeting PCSK9:

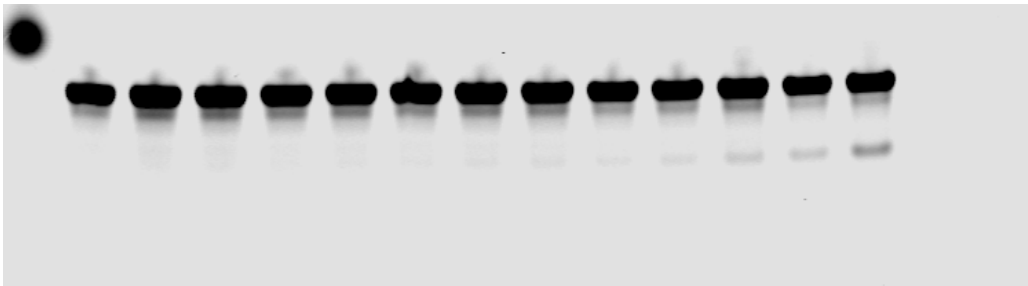

- S (16 nt)
- P (8 nt)

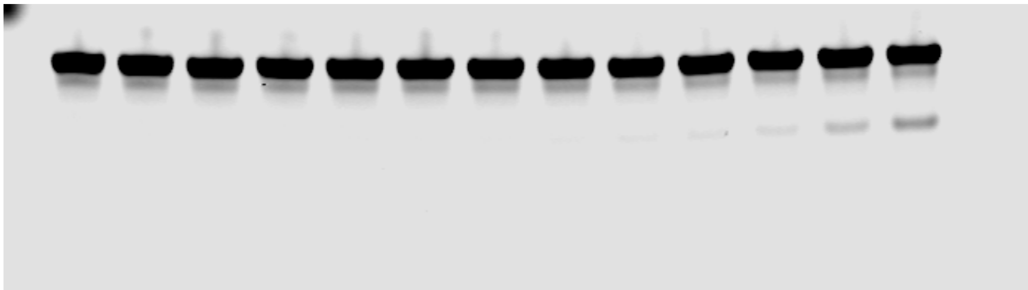

- S (16 nt)
- P (8 nt)

## Dz 1 targeting HTT

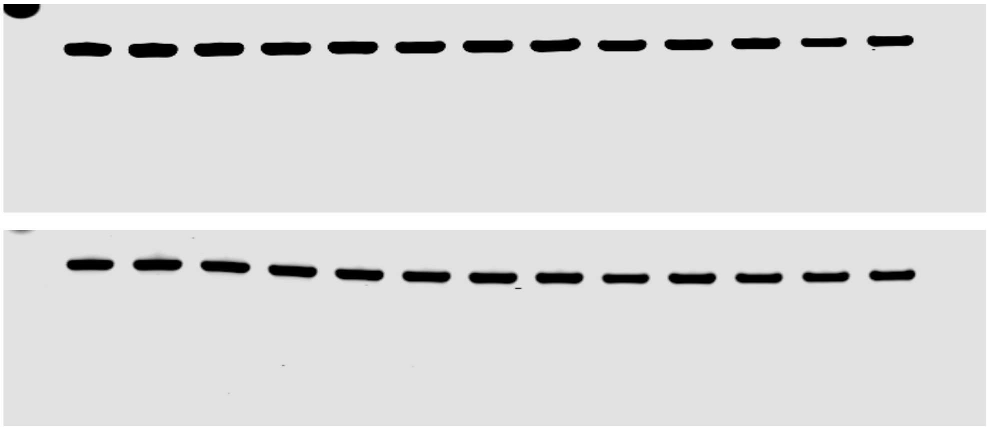


## Dz 1 targeting GATA3 (UGUU)

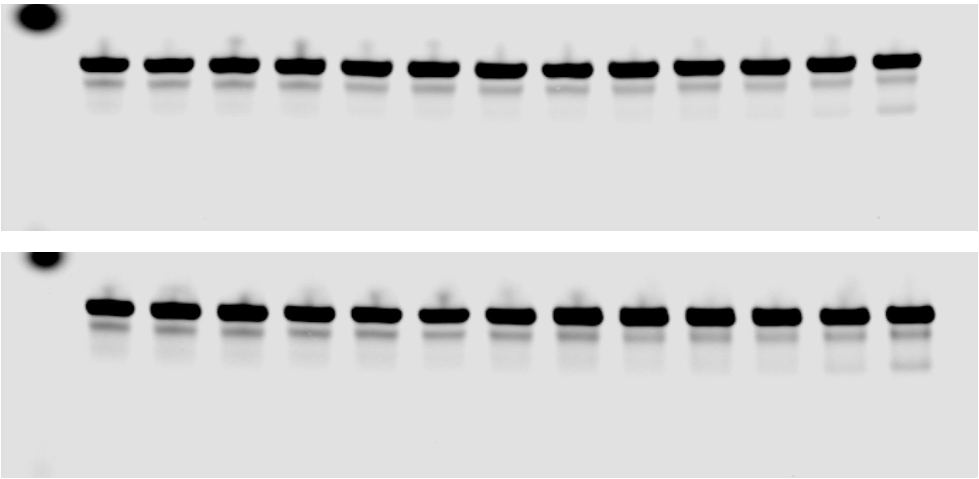


## Dz 1 targeting cjun (UGUU)

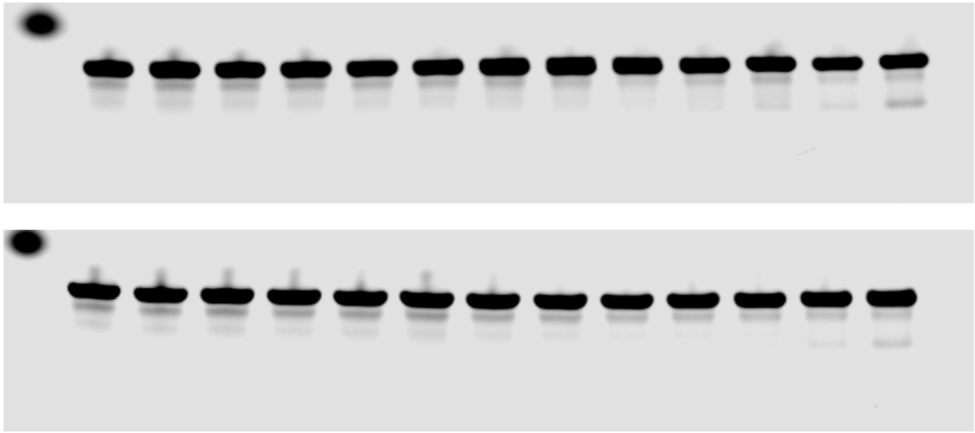

| | Dz 1 targeting PCSK9 % RNA cleavage | |
|---|---|---|
| Time (min) | rep. 1 | rep. 2 |
| 0 | 0.31 | 0.29 |
| 0.5 | 0.27 | 0.22 |
| 1 | 0.31 | 0.23 |
| 2 | 0.34 | 0.21 |
| 3 | 0.41 | 0.22 |
| 4 | 0.68 | 0.23 |
| 5 | 0.29 | 0.42 |
| 6 | 0.32 | 0.57 |
| 8 | 0.54 | 0.97 |
| 10 | 1.08 | 1.1 |
| 15 | 1.44 | 1.2 |
| 30 | 2.2 | 2.15 |
| 60 | 3.7 | 3.51 |
| $V_0$ (nM $min^{-1}$) | 0.55 | 0.53 |

| | Dz 1 targeting GATA3 (UGUU) % RNA cleavage | |
|---|---|---|
| Time (min) | rep. 1 | rep. 2 |
| 0 | 0.32 | 0.33 |
| 0.5 | 0.67 | 0.54 |
| 1 | 0.42 | 0.74 |
| 2 | 0.66 | 0.87 |
| 3 | 0.36 | 0.9 |
| 4 | 0.48 | 0.94 |
| 5 | 0.56 | 0.86 |
| 6 | 0.77 | 0.91 |
| 8 | 0.88 | 0.96 |
| 10 | 1.05 | 1.06 |
| 15 | 1.08 | 1.23 |
| 30 | 1.35 | 1.63 |
| 60 | 3.32 | 2.53 |
| $V_0$ (nM $min^{-1}$) | 0.49 | 0.35 |

| | Dz 1 targeting cjun (UGUU) % RNA cleavage | |
|---|---|---|
| Time (min) | rep. 1 | rep. 2 |
| 0 | 0.54 | 0.26 |
| 0.5 | 0.53 | 0.32 |
| 1 | 0.59 | 0.38 |
| 2 | 0.66 | 0.41 |
| 3 | 0.68 | 0.42 |
| 4 | 0.71 | 0.64 |
| 5 | 0.72 | 0.78 |
| 6 | 0.81 | 0.91 |
| 8 | 1.14 | 0.87 |
| 10 | 1.27 | 1.08 |
| 15 | 1.42 | 1.18 |
| 30 | 2.3 | 1.68 |
| 60 | 4.1 | 3.04 |
| $V_0$ (nM min$^{-1}$) | 0.59 | 0.45 |

Gels/data analysis for the Dz 46 variants targeting these sequences are in the Source Data for Main Text Figure 2C

## Figure S14-B:

### DV15E4

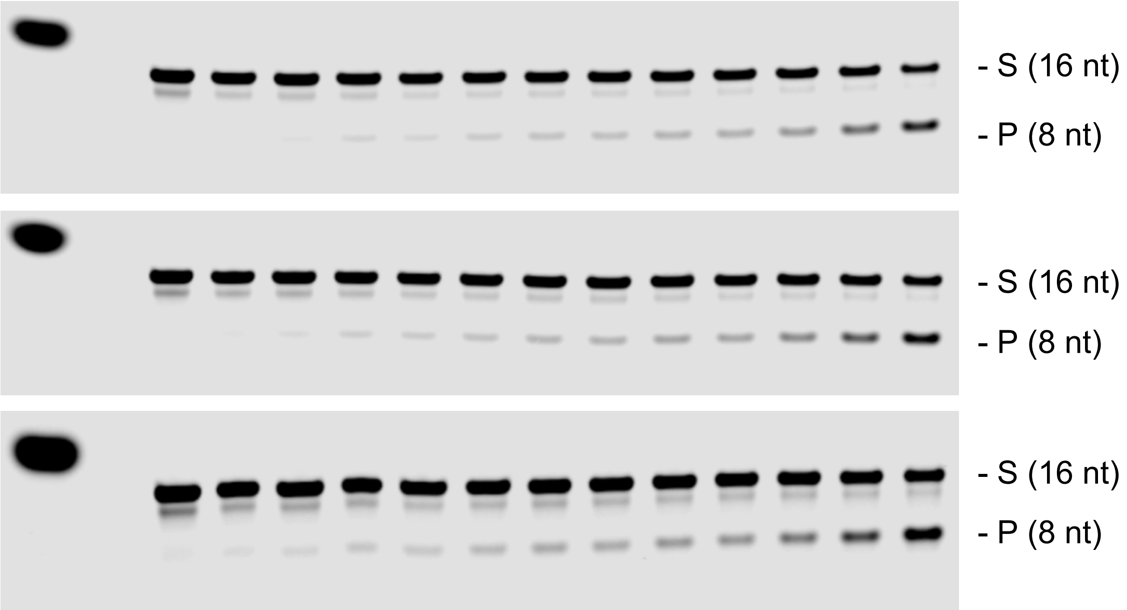

**DH5E**

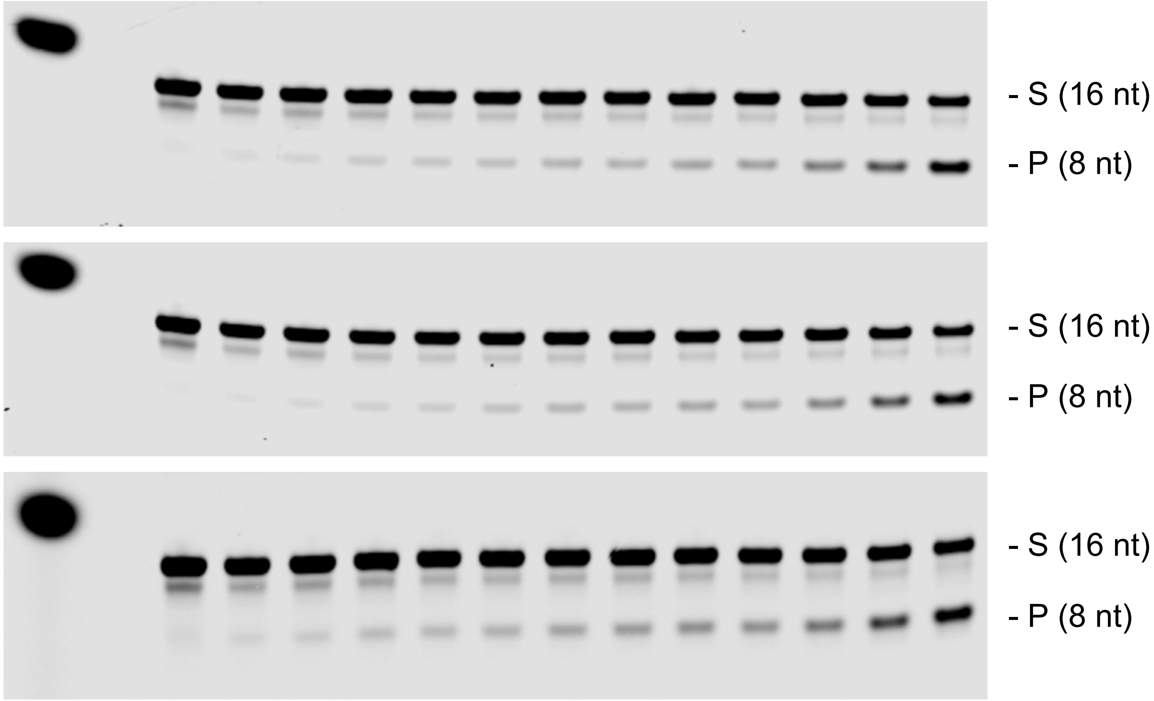


**Figure S14-C:**

| MTO (100S:1E) Time (minutes) | DV15E4, r1 | DV15E4, r2 | DV15E4, r3 | Avg |
|---|---|---|---|---|
| 0 | 0.64 | 0.63 | 0.27 | 0.51 |
| 0.5 | 1.51 | 1.33 | 1.06 | 1.30 |
| 1 | 1.81 | 1.83 | 1.48 | 1.71 |
| 2 | 3.20 | 3.24 | 2.41 | 2.95 |
| 3 | 4.01 | 4.15 | 3.53 | 3.90 |
| 4 | 5.34 | 5.36 | 4.64 | 5.11 |
| 5 | 6.36 | 6.14 | 5.40 | 5.97 |
| 6 | 7.03 | 6.77 | 6.00 | 6.60 |
| 8 | 8.94 | 8.98 | 7.98 | 8.63 |
| 10 | 10.67 | 10.69 | 9.42 | 10.26 |
| 15 | 15.51 | 14.44 | 13.39 | 14.44 |
| 30 | 25.85 | 23.94 | 22.55 | 24.11 |
| 60 | 46.43 | 42.69 | 41.67 | 43.59 |
| Vini | 10.03 | 10.05 | 8.75 | 9.61 |
| Stdev | | | | 0.75 |

| MTO (100S:1E) Time (minutes) | DH5E, r1 | DH5E, r2 | DH5E, r3 | Avg |
|---|---|---|---|---|
| 0 | 0.64 | 0.66 | 0.54 | 0.61 |
| 0.5 | 1.40 | 1.28 | 1.19 | 1.29 |
| 1 | 1.89 | 1.82 | 1.76 | 1.82 |
| 2 | 2.85 | 2.90 | 2.87 | 2.87 |
| 3 | 4.01 | 3.85 | 3.90 | 3.92 |
| 4 | 4.64 | 5.33 | 4.57 | 4.84 |
| 5 | 5.46 | 6.25 | 5.84 | 5.85 |
| 6 | 6.29 | 6.53 | 6.73 | 6.52 |
| 8 | 8.13 | 9.28 | 8.26 | 8.56 |
| 10 | 9.64 | 9.82 | 9.78 | 9.75 |
| 15 | 14.49 | 14.17 | 13.74 | 14.13 |
| 30 | 24.37 | 25.75 | 23.58 | 24.57 |
| 60 | 46.34 | 44.52 | 41.34 | 44.07 |
| Vini | 9.23 | 9.01 | 8.80 | 9.01 |
| Stdev | | | | 0.18 |

**Figure S15-C:**
**Dz-1**

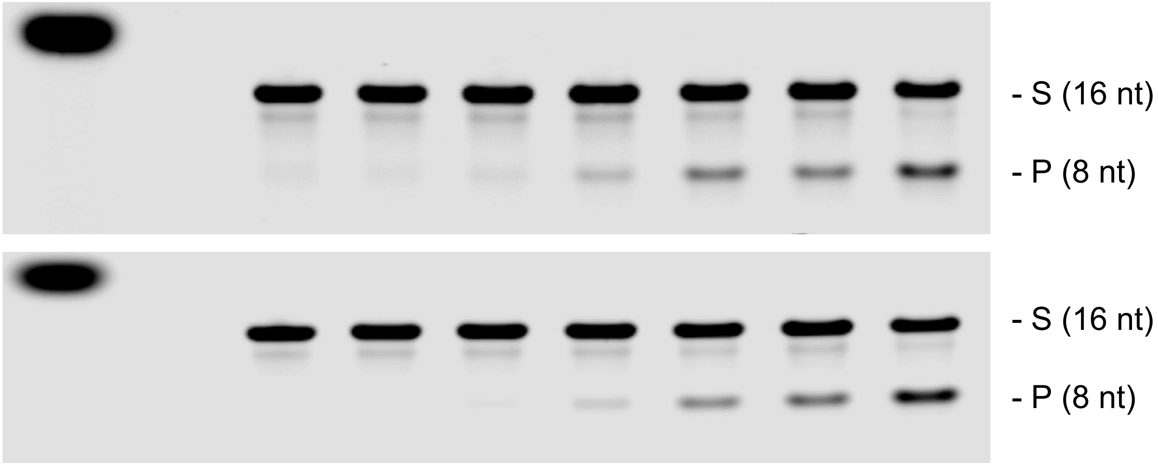

**Dz-38**

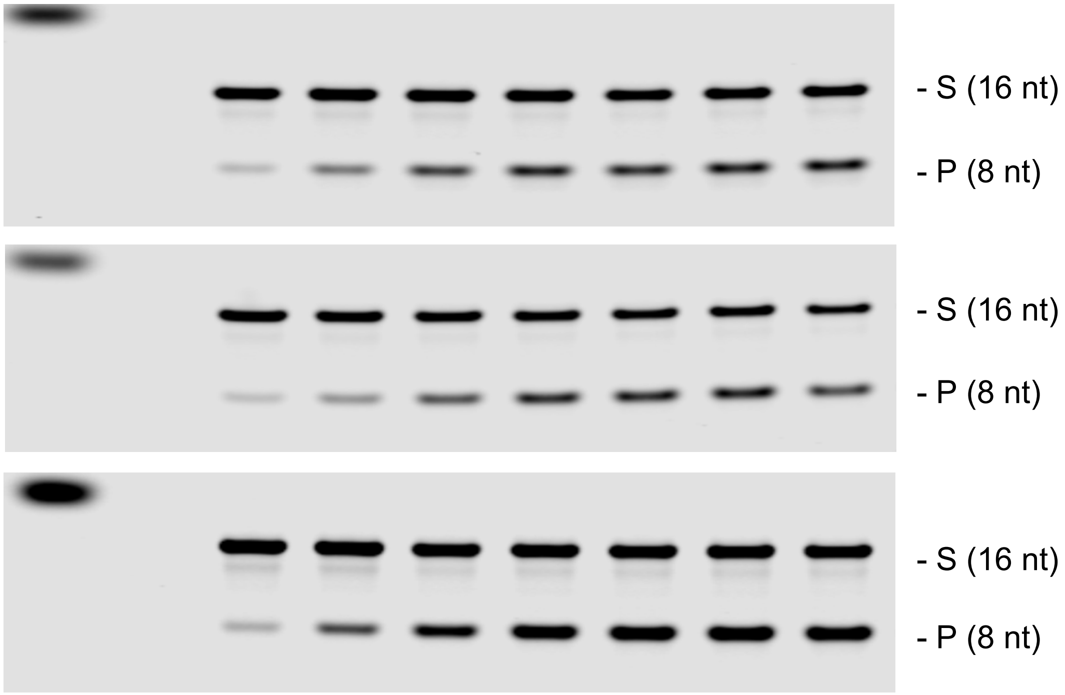
- S (16 nt)
- P (8 nt)
- S (16 nt)
- P (8 nt)
- S (16 nt)
- P (8 nt)

**Dz-42**

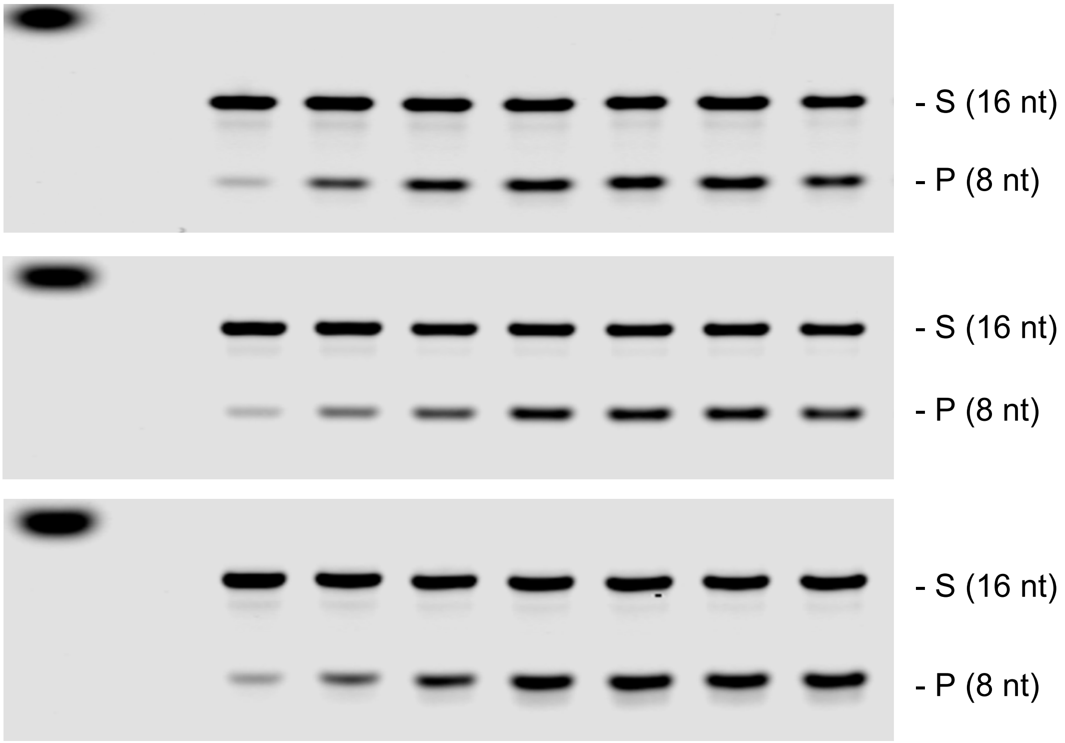
- S (16 nt)
- P (8 nt)
- S (16 nt)
- P (8 nt)
- S (16 nt)
- P (8 nt)

**Dz-46**

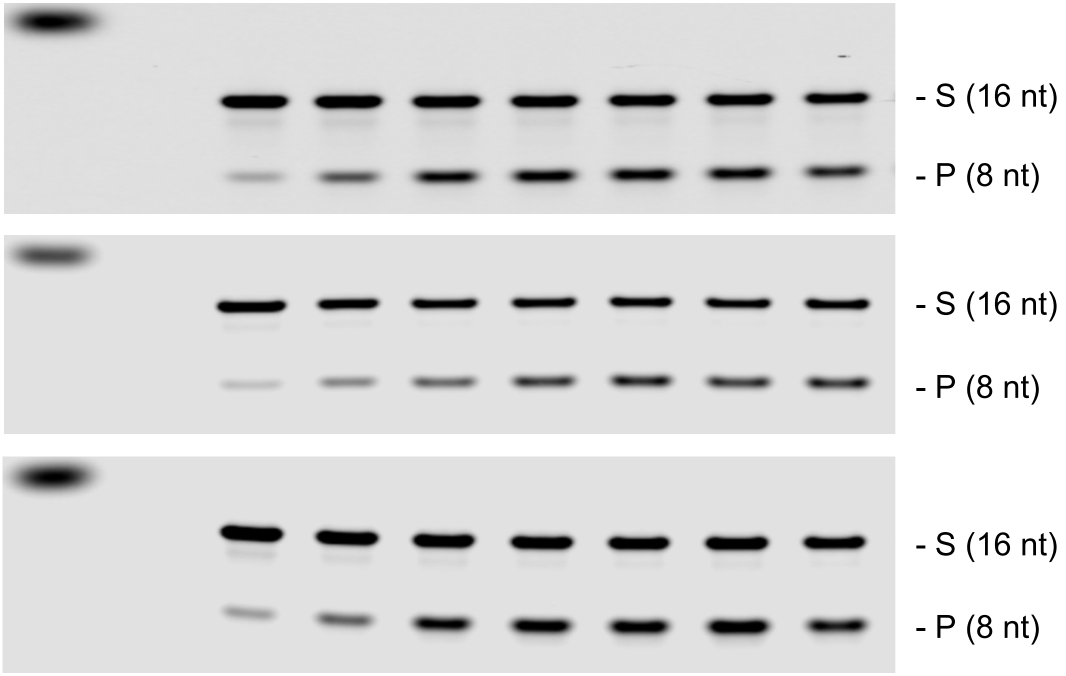


## Figure S15-D:

| MTO (100S:1E) $[MgCl_2]$ mM | Dz-1 | Dz-38 | Dz-42 | Dz-46 | SD, Dz-1 | SD, Dz-38 | SD, Dz-42 | SD, Dz-46 |
|---|---|---|---|---|---|---|---|---|
| 0.00 | 1.14 | 6.26 | 6.92 | 7.44 | 0.07 | 1.26 | 1.29 | 0.73 |
| 0.10 | 1.13 | 14.99 | 17.68 | 19.87 | 0.06 | 1.34 | 0.33 | 1.06 |
| 0.25 | 1.53 | 27.31 | 28.81 | 32.63 | 0.14 | 3.53 | 0.44 | 0.55 |
| 0.50 | 3.90 | 35.09 | 36.72 | 38.48 | 0.50 | 5.25 | 2.40 | 1.87 |
| 1.00 | 12.61 | 38.62 | 38.41 | 40.21 | 0.73 | 4.41 | 2.68 | 1.35 |
| 2.50 | 13.29 | 38.76 | 37.33 | 40.67 | 0.91 | 4.69 | 3.58 | 2.19 |
| 5.00 | 30.70 | 39.62 | 37.85 | 39.19 | 3.38 | 3.55 | 1.95 | 1.94 |

| MTO (100S:1E) 20 min $[MgCl_2]$ mM | Dz-1, r1 | Dz-1, r2 | Avg |
|---|---|---|---|
| 0.00 | 0.99 | 1.28 | 1.14 |
| 0.10 | 1.06 | 1.19 | 1.13 |
| 0.25 | 1.30 | 1.76 | 1.53 |
| 0.50 | 3.73 | 4.07 | 3.90 |
| 1.00 | 12.28 | 12.93 | 12.61 |
| 2.50 | 11.64 | 14.94 | 13.29 |
| 5.00 | 28.44 | 32.96 | 30.70 |

| MTO (100S:1E) 20 min [MgCl2] mM | Dz-38, r1 | Dz-38, r2 | Dz-38, r3 | Avg |
|---|---|---|---|---|
| 0.00 | 6.67 | 7.26 | 4.84 | 6.26 |
| 0.10 | 15.17 | 16.23 | 13.57 | 14.99 |
| 0.25 | 23.27 | 29.77 | 28.90 | 27.31 |
| 0.50 | 29.04 | 37.79 | 38.44 | 35.09 |
| 1.00 | 33.54 | 41.46 | 40.85 | 38.62 |
| 2.50 | 33.39 | 40.85 | 42.04 | 38.76 |
| 5.00 | 35.54 | 41.33 | 42.00 | 39.62 |

| MTO (100S:1E) 20 min [$MgCl_2$] mM | Dz-42, r1 | Dz-42, r2 | Dz-42, r3 | Avg |
|---|---|---|---|---|
| 0.00 | 5.65 | 6.89 | 8.23 | 6.92 |
| 0.10 | 18.00 | 17.34 | 17.71 | 17.68 |
| 0.25 | 28.36 | 29.23 | 28.83 | 28.81 |
| 0.50 | 35.43 | 35.23 | 39.49 | 36.72 |
| 1.00 | 36.06 | 37.86 | 41.33 | 38.41 |
| 2.50 | 33.87 | 37.10 | 41.01 | 37.33 |
| 5.00 | 36.10 | 37.50 | 39.95 | 37.85 |

| MTO (100S:1E) 20 min [$MgCl_2$] mM | Dz-46, r1 | Dz-46, r2 | Dz-46, r3 | Avg |
|---|---|---|---|---|
| 0.00 | 7.65 | 6.62 | 8.04 | 7.44 |
| 0.10 | 19.21 | 21.09 | 19.30 | 19.87 |
| 0.25 | 31.99 | 32.95 | 32.95 | 32.63 |
| 0.50 | 36.49 | 40.21 | 38.74 | 38.48 |
| 1.00 | 38.67 | 41.23 | 40.71 | 40.21 |
| 2.50 | 38.14 | 42.07 | 41.80 | 40.67 |
| 5.00 | 37.57 | 41.35 | 38.67 | 39.19 |

## Figure S16-A

### No ATP

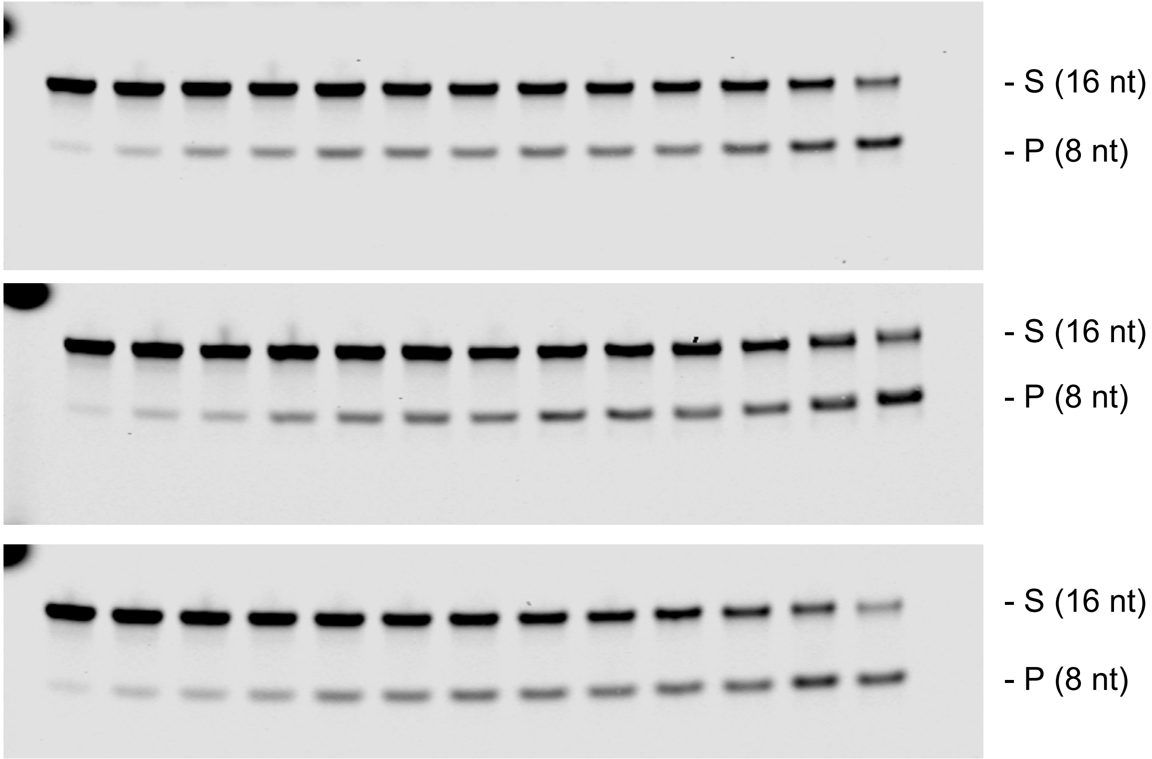


### 1 mM ATP

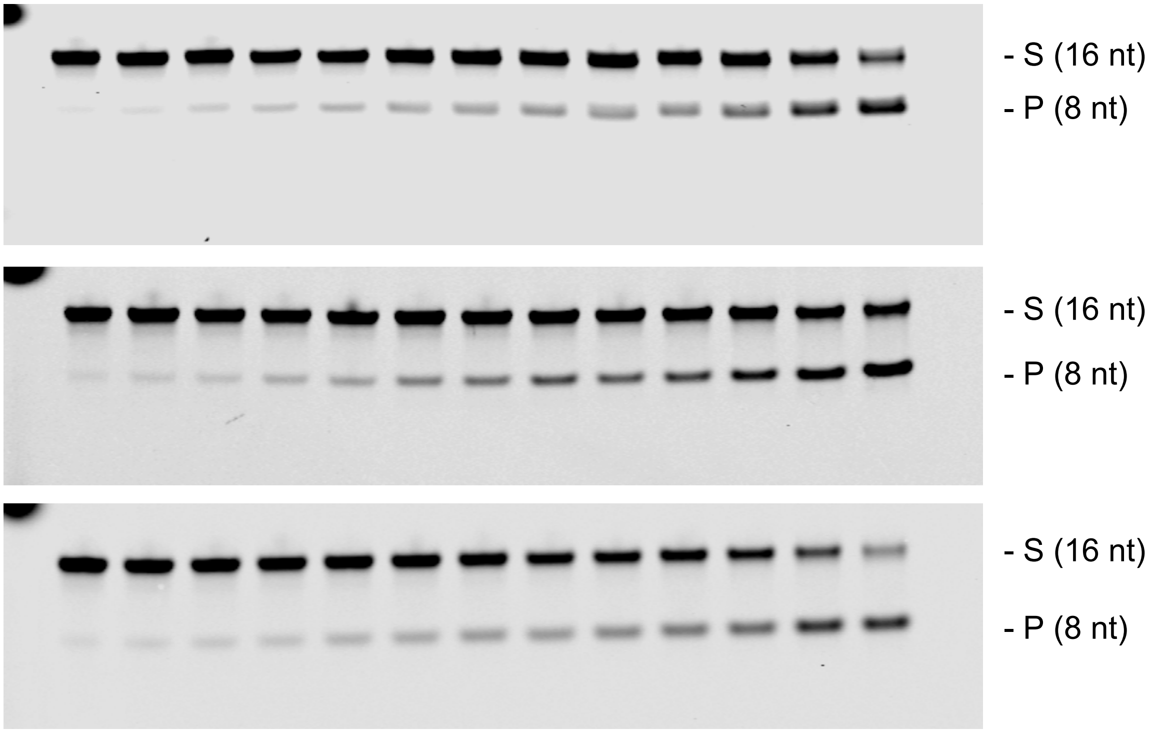

| Normalized rates | |
|---|---|
| NO ATP | 1 mM ATP |
| 1.031471 | 0.498033 |
| 0.998426 | 0.554681 |
| 0.970102 | 0.632573 |

## Figure S16-B & -C

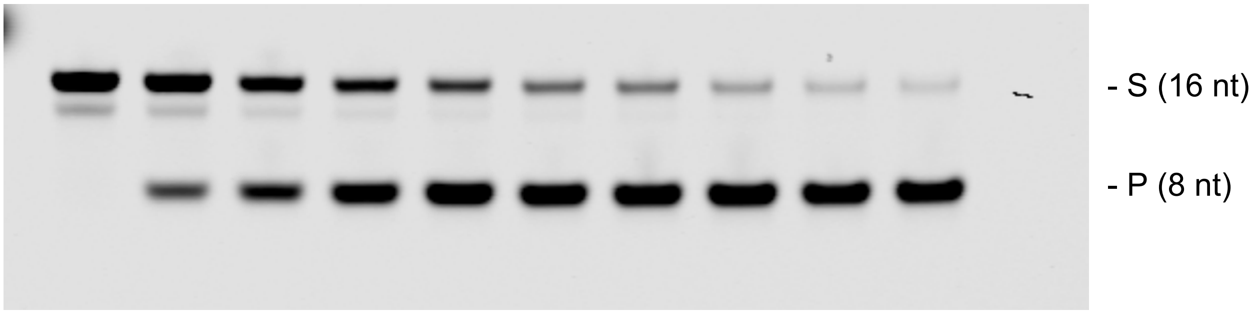


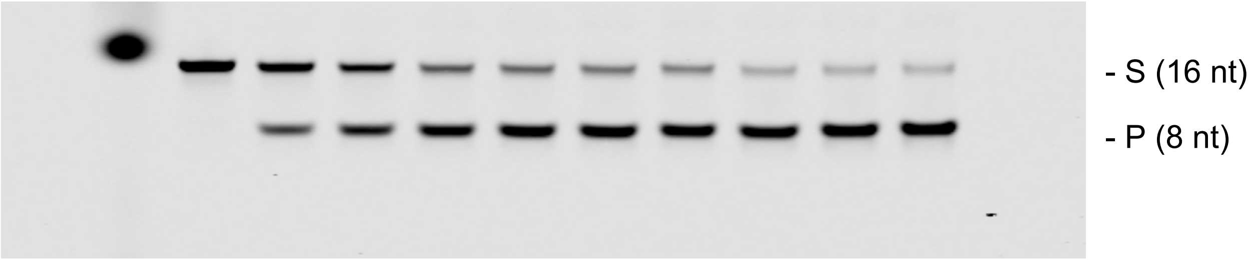


| | Dz 46 0.25 mM $MgCl_2$ %RNA cleavage | |
|---|---|---|
| Time (min) | rep 1 | rep 2 |
| 0.5 | 25.74 | 34.93 |
| 1 | 50.27 | 52.54 |
| 2 | 67.99 | 68.55 |
| 3 | 74.8 | 73.97 |
| 4 | 80.08 | 76.03 |
| 5 | 80.9 | 78.26 |
| 15 | 86.46 | 85 |
| 30 | 88.64 | 87.4 |
| 60 | 90.66 | 88.95 |

**Figure S17-A:**
**Dz 1 variants at 1 mM $MgCl_2$ (all gels are loaded in the same order-substrate, 0, 1, 5, 10, 15, 30, 60, 120 mins):**

**UGUU**

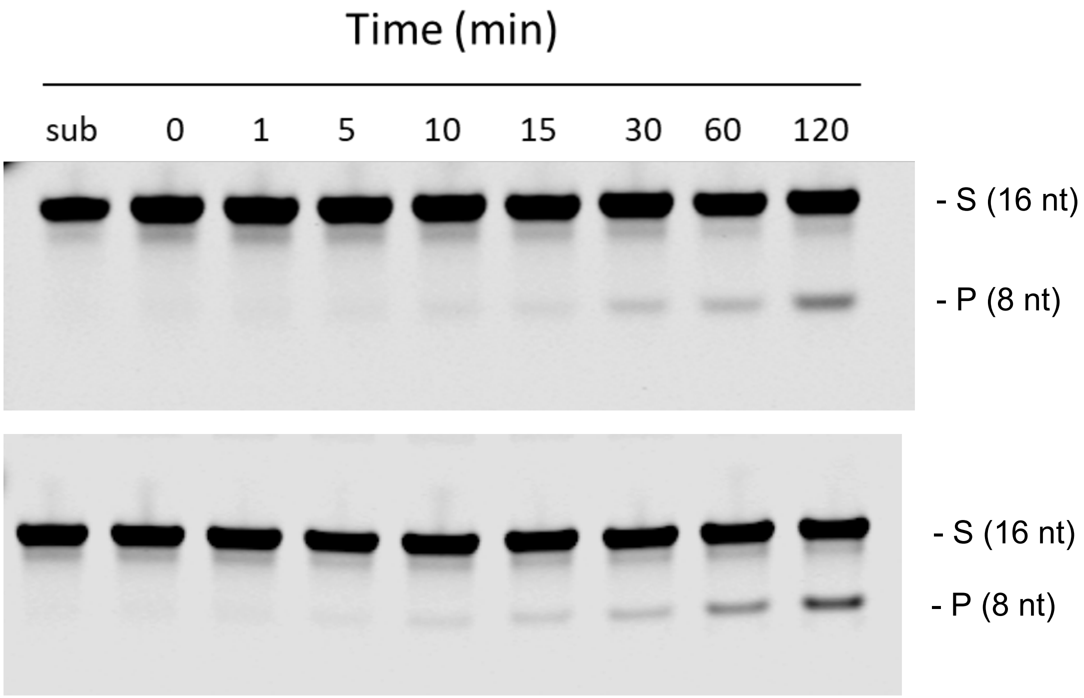


**UGUC**

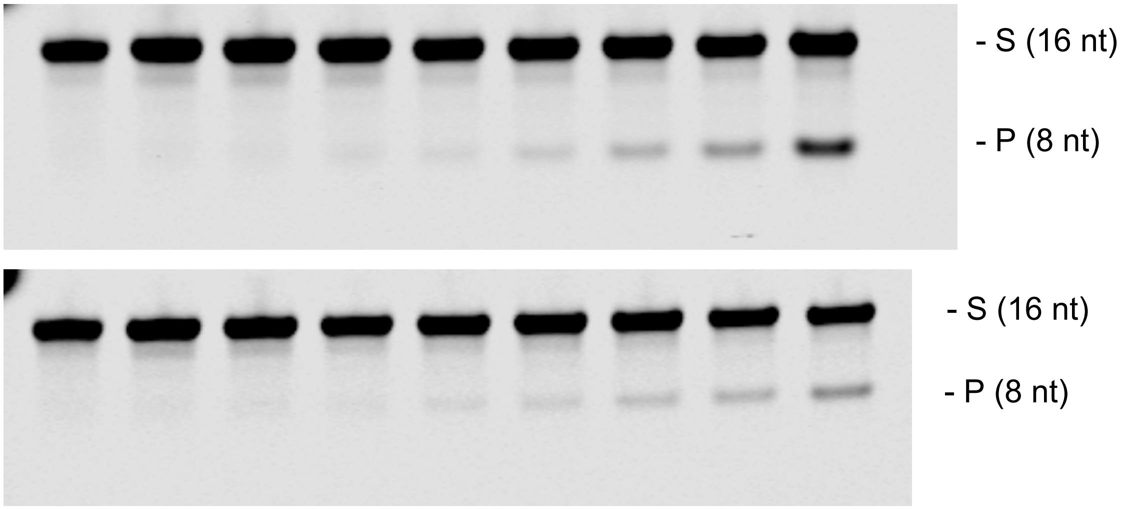

**UGUG**

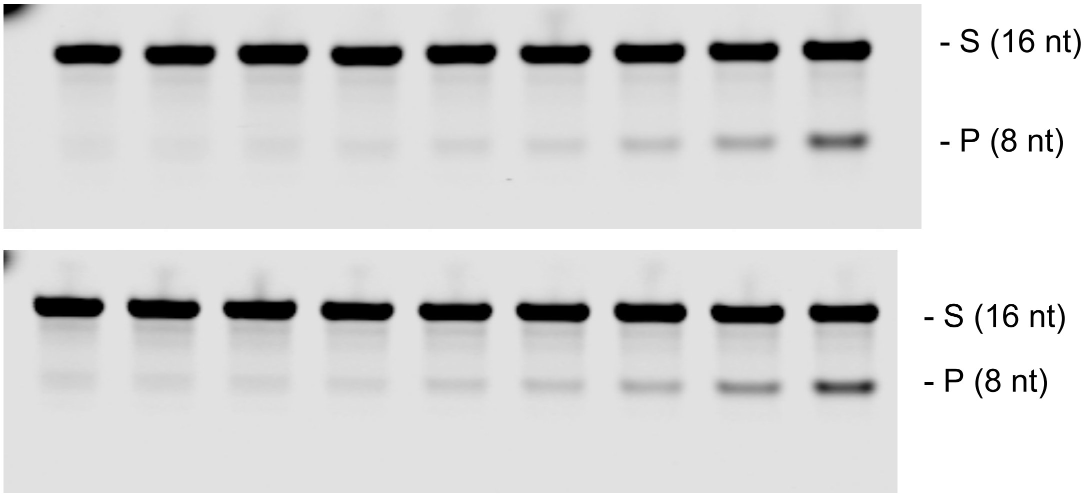


**UGUA**

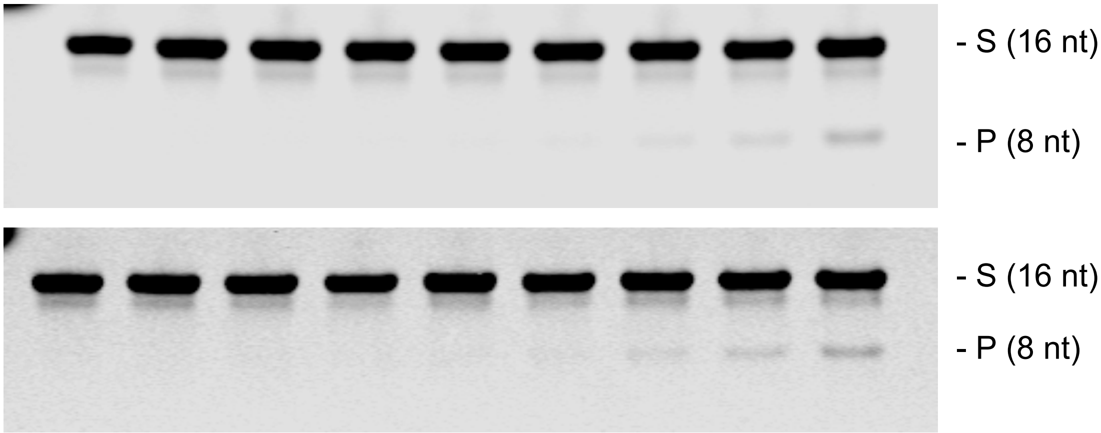


**CGUU**

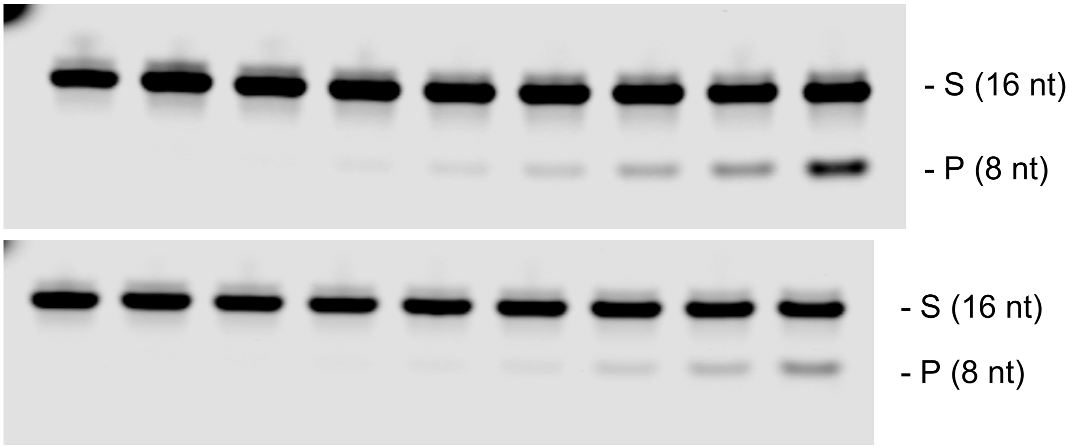

**GGUU**

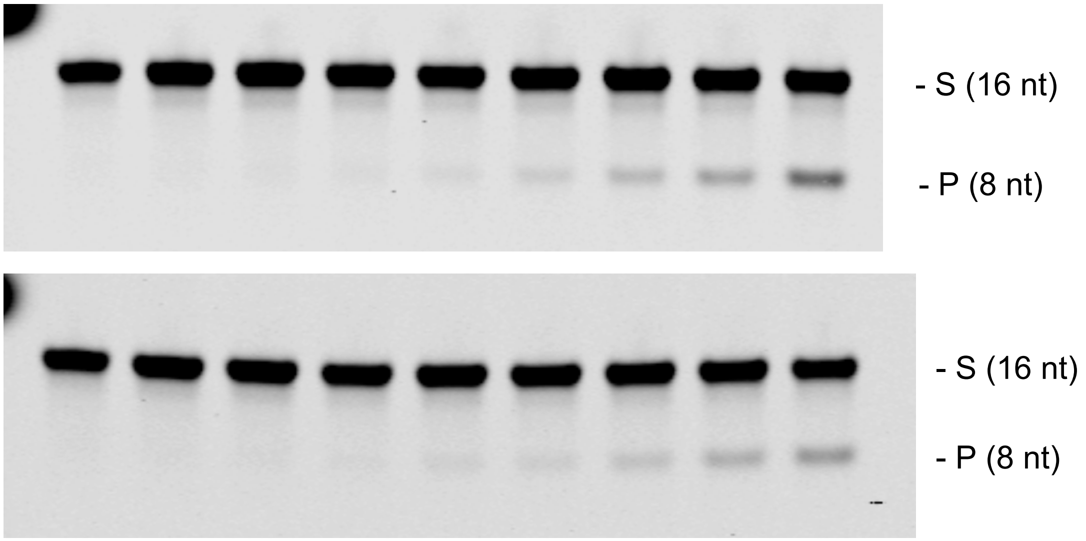


**AGUU**

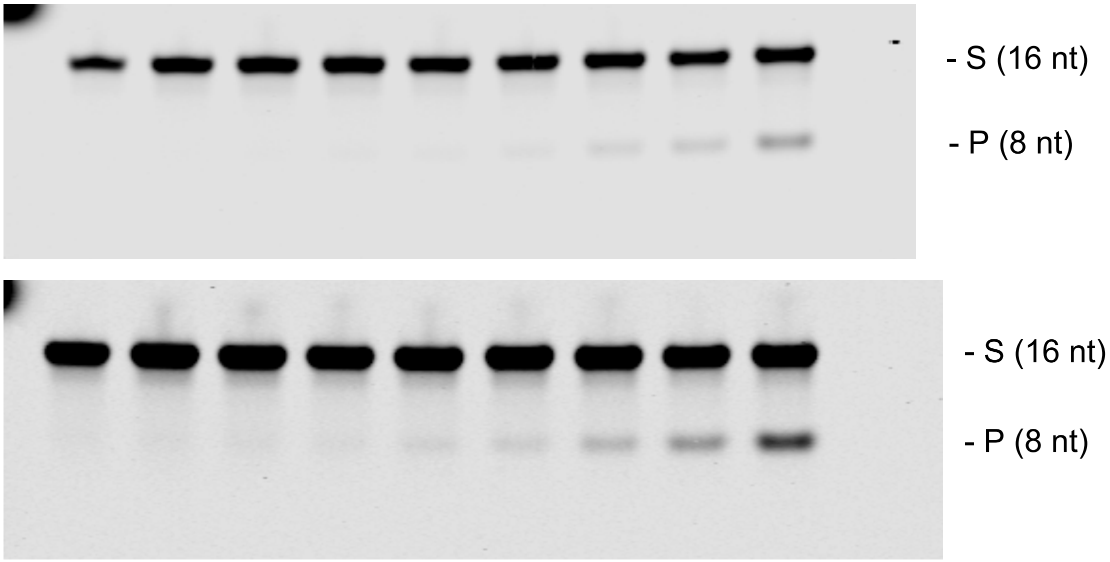


| | | 1 mM $MgCl_2$ Dz 1 variants | | | | | | |
|---|---|---|---|---|---|---|---|---|
| | | UGUU | UGU**C** | UGU**G** | UGU**A** | **C**GUU | **G**GUU | **A**GUU |
| normalized rate | rep 1 | 0.88 | 2.03 | 1.79 | 0.57 | 2.73 | 1.82 | 1.92 |
| | rep2 | 1.12 | 1.25 | 1.61 | 0.44 | 2.18 | 1.74 | 1.95 |

**Figure S17-B :**
**Dz 1 variants at 5 mM $MgCl_2$ (all gels are loaded in the same order- 0, 0.5, 1, 2, 3, 4, 5, 6, 8, 10, 15, 30, 60 mins):**

**UGUU**

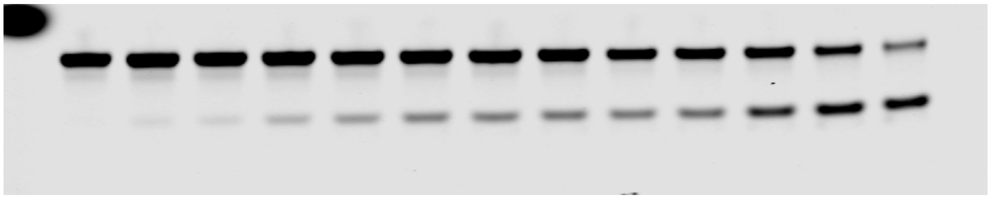

- S (16 nt)
- P (8 nt)

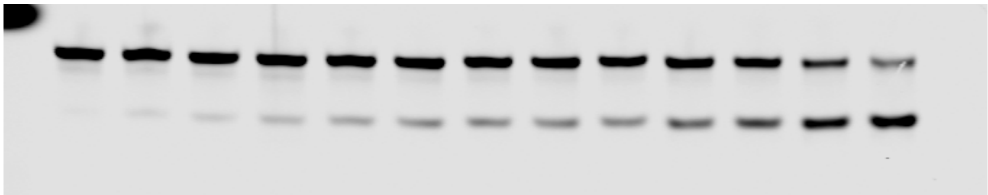

- S (16 nt)
- P (8 nt)

**UGUC**

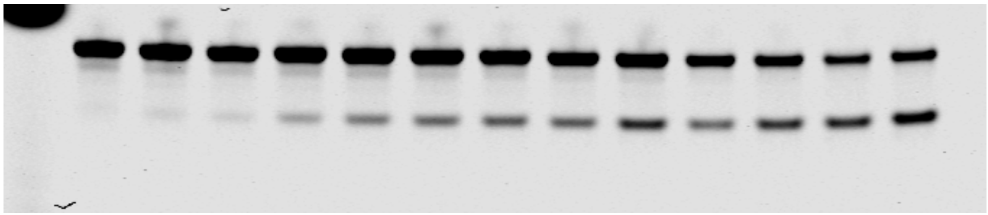

- S (16 nt)
- P (8 nt)

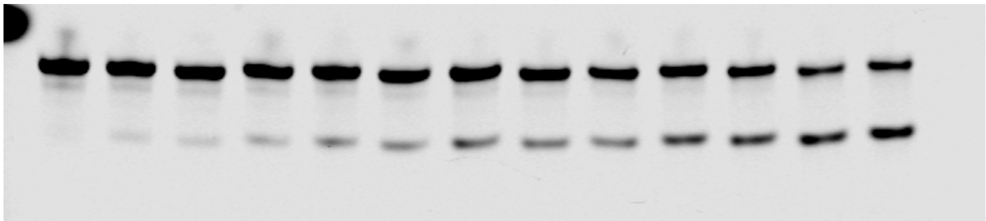

- S (16 nt)
- P (8 nt)

**UGUG**

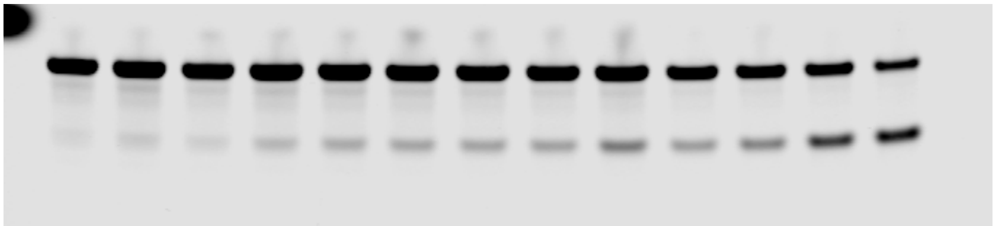

- S (16 nt)
- P (8 nt)

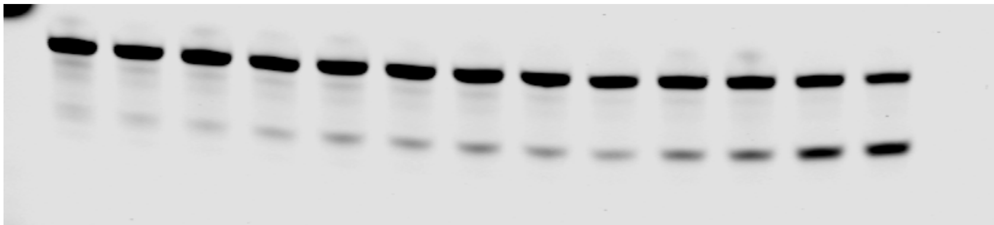

- S (16 nt)
- P (8 nt)

**UGUA**

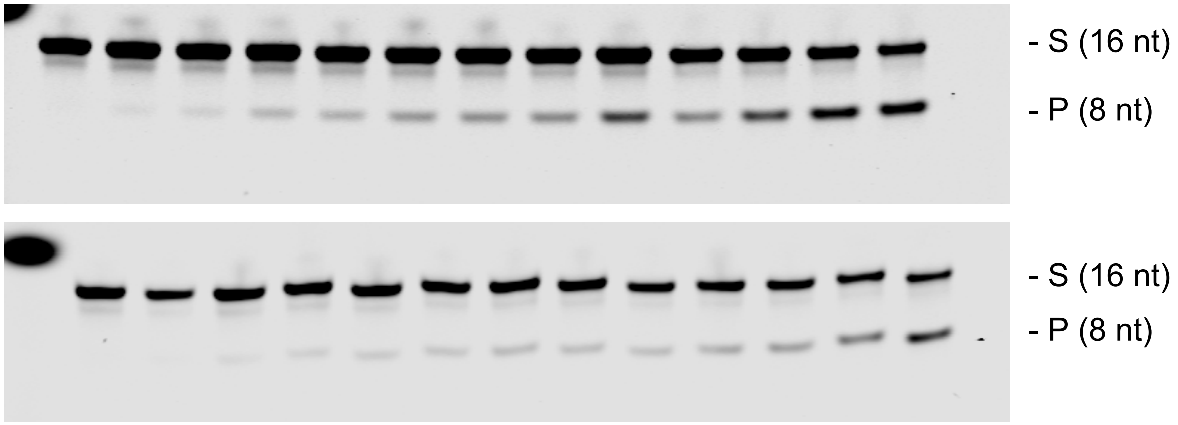


**CGUU**

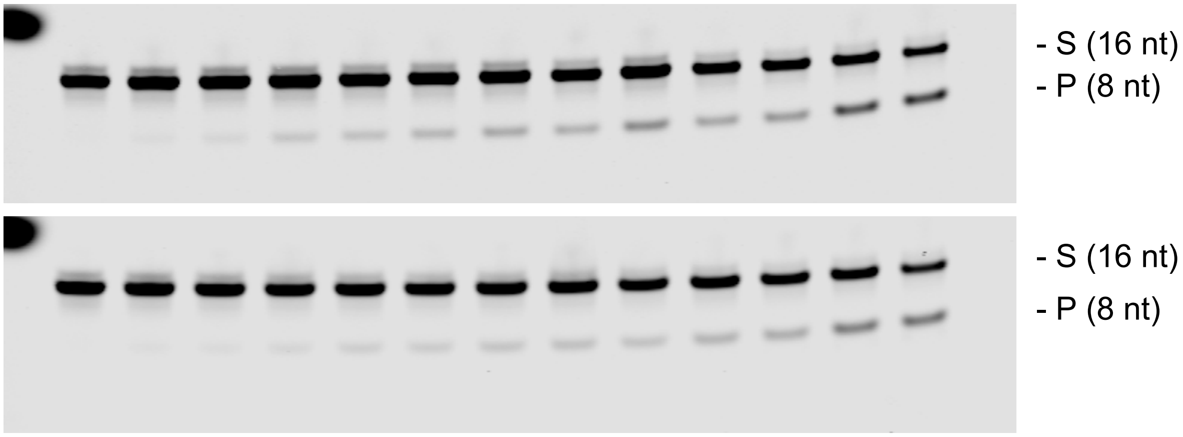


**GGUU**

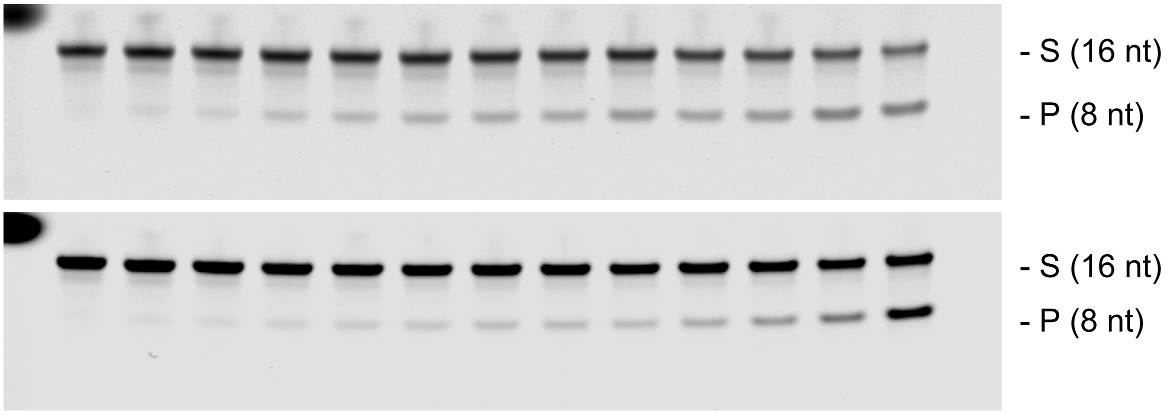

**AGUU**

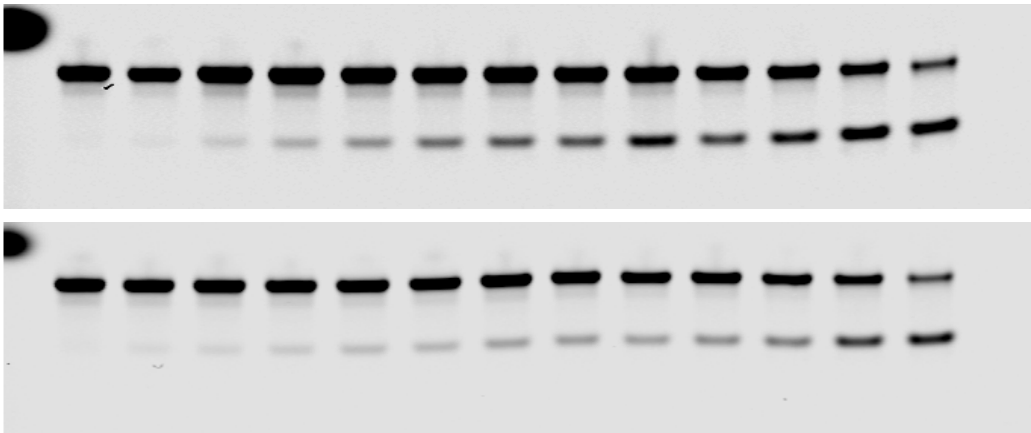

- S (16 nt)
- P (8 nt)
- S (16 nt)
- P (8 nt)

| | | 5 mM $MgCl_2$ Dz 1 variants | | | | | | |
|---|---|---|---|---|---|---|---|---|
| | | UGUU | UGU**G** | UGU**C** | UGU**A** | **C**GUU | **G**GUU | **A**GUU |
| normalized rate | rep 1 | 0.91 | 0.38 | 1.03 | 0.51 | 0.49 | 0.45 | 0.78 |
| | rep2 | 1.09 | 0.41 | 1.01 | 0.48 | 0.43 | 0.38 | 0.58 |

**Figure S18:**
**UGUU**

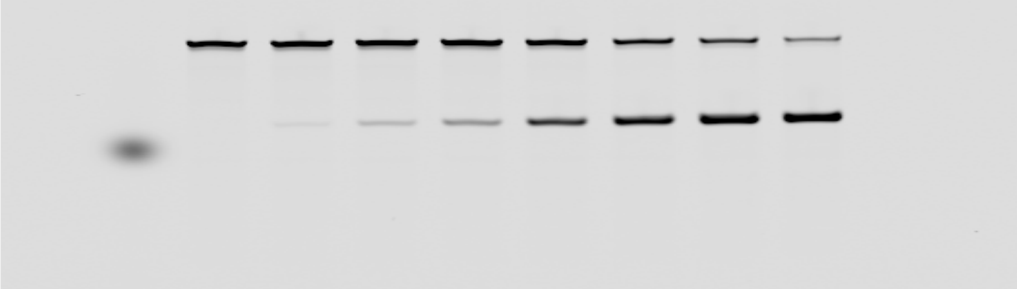

- S (60 nt)
- P (30 nt)

**AGUU**

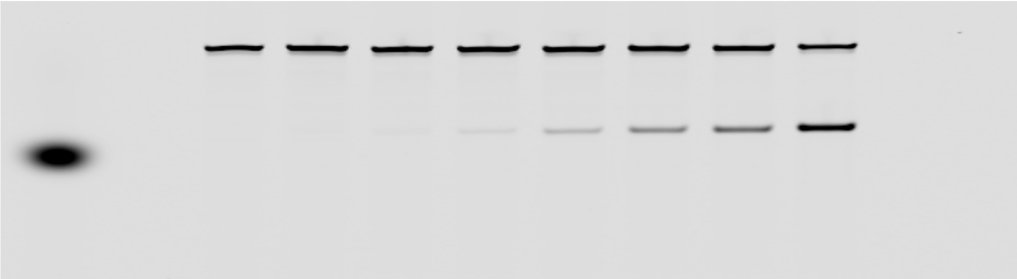

- S (60 nt)
- P (30 nt)

**Figure S19:**

**UGUU**

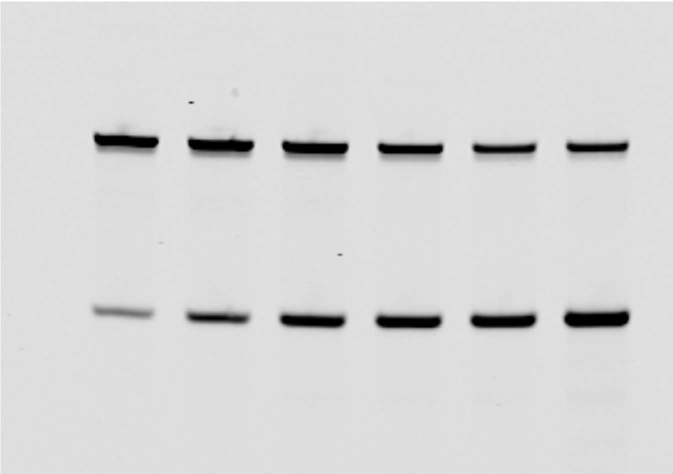

- S (60 nt)

- P (30 nt)

**AGUU**

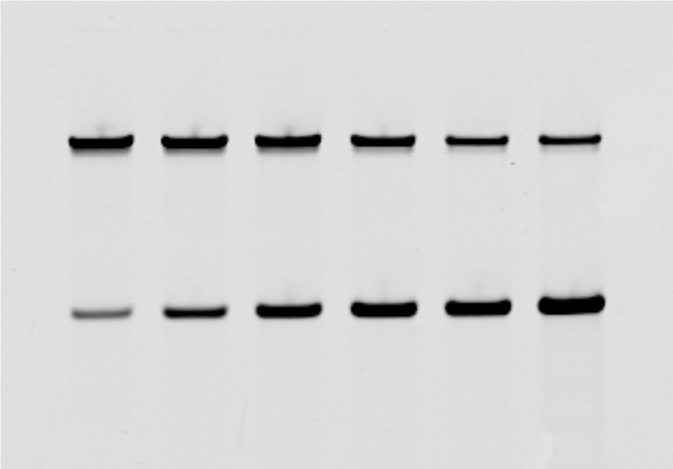

- S (60 nt)

- P (30 nt)

**Figure S20:**

**Cy5:**

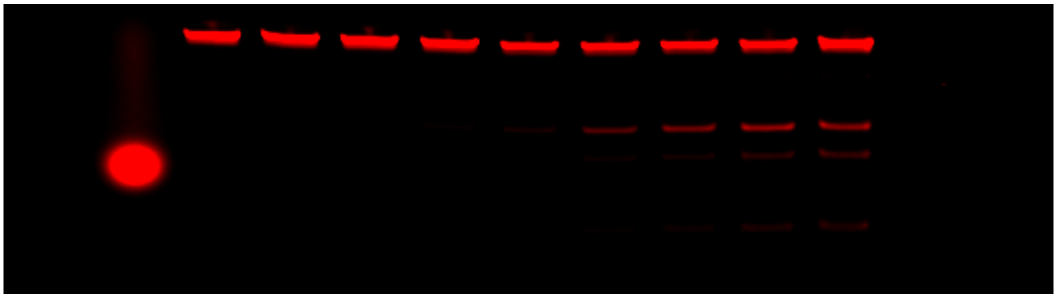

- S (60 nt)

**AF750:**

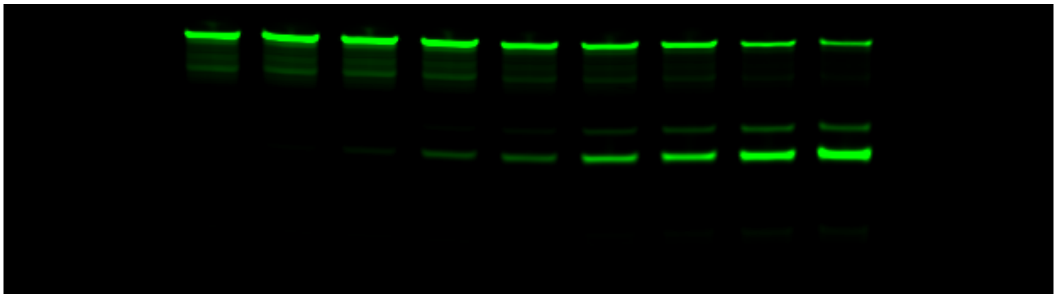

- S (60 nt)
- P (30 nt)

**Merge:**

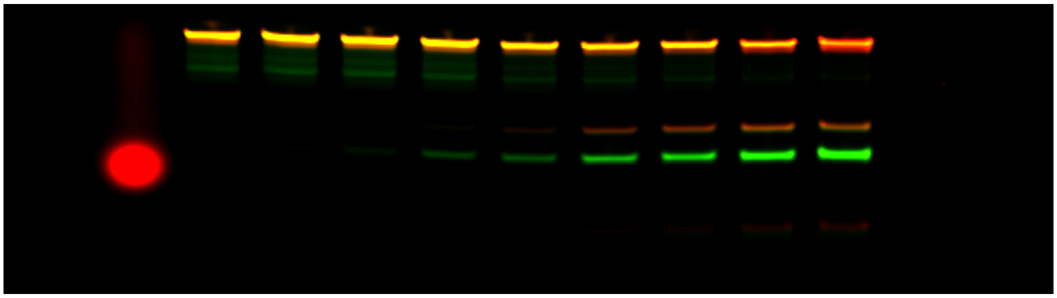

- S (60 nt)
- P (30 nt)

**Figure S21-A:**

| 6h Dz 46 | G12V | WT | STDEV-G12V | STDEV-WT |
|---|---|---|---|---|
| Untreated | 132678.52 | 61223.70 | 74003.72 | 27621.48 |
| Dz (50 nM) | 166160.69 | 83519.21 | 63080.29 | 28965.50 |
| Dz (500 nM) | 81881.27 | 95343.80 | 31410.36 | 37693.90 |
| | | | | |
| **12h Dz 46** | **G12V** | **WT** | **STDEV-G12V** | **STDEV-WT** |
| Untreated | 83703.50 | 39435.65 | 65405.15 | 22997.90 |
| Dz (50 nM) | 107470.06 | 58818.02 | 66464.59 | 27143.09 |
| Dz (500 nM) | 76520.76 | 89666.96 | 29474.92 | 32657.03 |
| | | | | |
| **24h Dz 46** | **G12V** | **WT** | **STDEV-G12V** | **STDEV-WT** |
| Untreated | 96227.76 | 41561.44 | 34874.65 | 15073.52 |
| Dz (50 nM) | 59588.71 | 41209.49 | 39940.62 | 26826.22 |
| Dz (500 nM) | 42014.73 | 42107.58 | 17637.75 | 12812.38 |

**Figure S21-B:**

**GAPDH transcript (235 nt)**

**6 h**

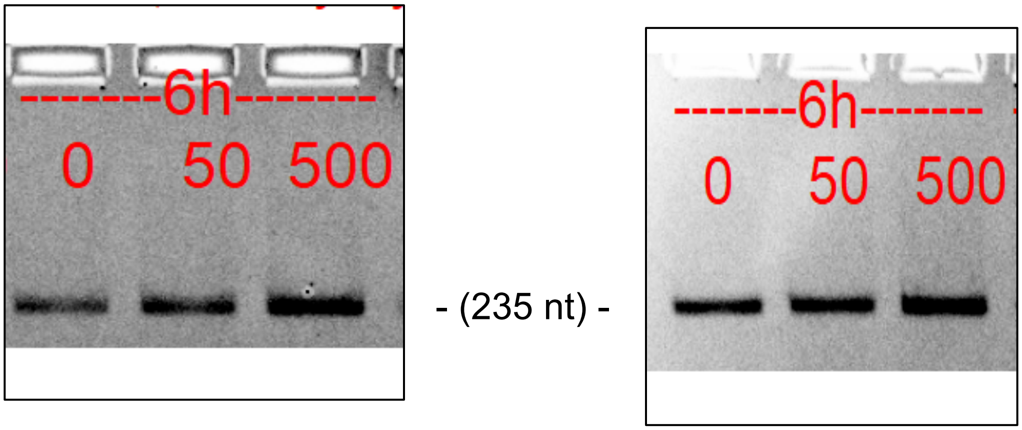

**12 h**

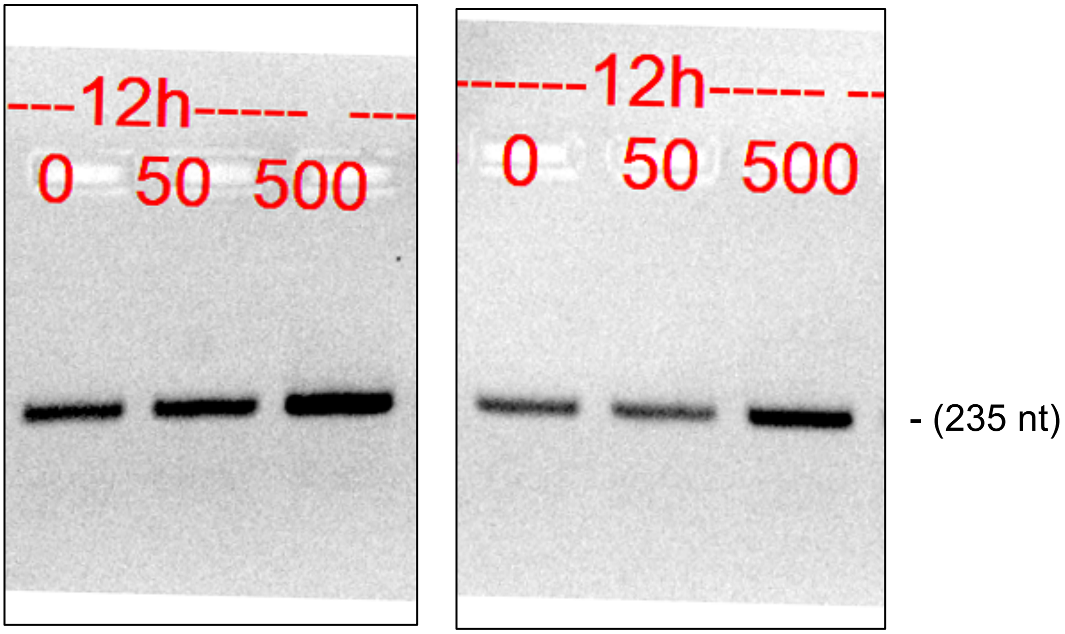


**24 h**

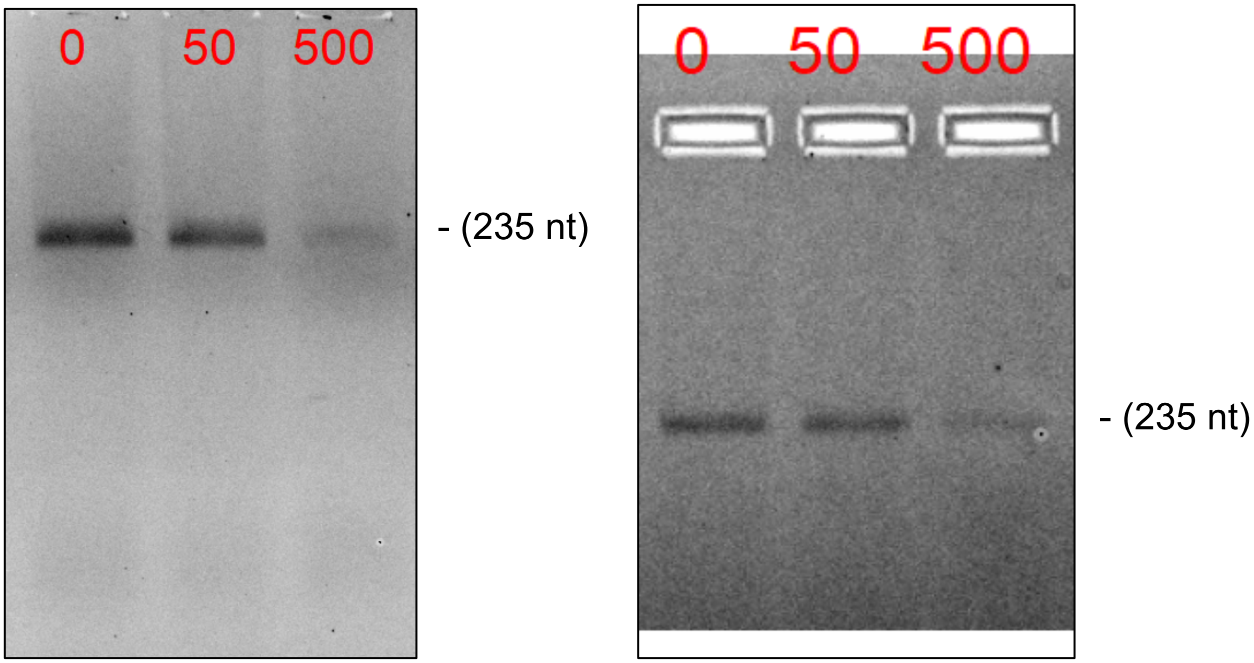

Supplement: Supplementary file 4 — Source Data [file 41467_2023_38100_MOESM4_ESM.zip › Source Data copy/Source Data_Supplementary Information.pdf]
